# Hepatocyte phosphatase DUSP22 mitigates NASH-HCC progression by targeting FAK

**DOI:** 10.1038/s41467-022-33493-5

**Published:** 2022-10-08

**Authors:** Chenxu Ge, Jun Tan, Xianling Dai, Qin Kuang, Shaoyu Zhong, Lili Lai, Chao Yi, Yan Sun, Jing Luo, Chufeng Zhang, Liancai Zhu, Bochu Wang, Minxuan Xu

**Affiliations:** 1 Chongqing Key Laboratory of Medicinal Resources in the Three Gorges Reservoir Region, School of Biological and Chemical Engineering, Chongqing University of Education, 400067 Chongqing, PR China; 2 Key Laboratory of Biorheological Science and Technology (Chongqing University), Ministry of Education, College of Bioengineering, Chongqing University, 400030 Chongqing, PR China; 3 Research Center of Brain Intellectual Promotion and Development for Children Aged 0-6 Years, Chongqing University of Education, 400067 Chongqing, PR China; 4 College of Modern Health Industry, Chongqing University of Education, 400067 Chongqing, PR China; 5 Shandong Cancer Hospital and Institute, Shandong First Medical University & Shandong Academy of Medical Sciences, 250117 Jinan, PR China

**Keywords:** Lipid signalling, Non-alcoholic steatohepatitis

## Abstract

Nonalcoholic steatohepatitis (NASH), a common clinical disease, is becoming a leading cause of hepatocellular carcinoma (HCC). Dual specificity phosphatase 22 (DUSP22, also known as JKAP or JSP-1) expressed in numerous tissues plays essential biological functions in immune responses and tumor growth. However, the effects of DUSP22 on NASH still remain unknown. Here, we find a significant decrease of DUSP22 expression in human and murine fatty liver, which is mediated by reactive oxygen species (ROS) generation. Hepatic-specific DUSP22 deletion particularly exacerbates lipid deposition, inflammatory response and fibrosis in liver, facilitating NASH and non-alcoholic fatty liver disease (NAFLD)-associated HCC progression. In contrast, transgenic over-expression, lentivirus or adeno-associated virus (AAV)-mediated DUSP22 gene therapy substantially inhibit NASH-related phenotypes and HCC development in mice. We provide mechanistic evidence that DUSP22 directly interacts with focal adhesion kinase (FAK) and restrains its phosphorylation at Tyr397 (Y397) and Y576 + Y577 residues, subsequently prohibiting downstream activation of extracellular signal-regulated kinase 1/2 (ERK1/2) and nuclear factor-κB (NF-κB) cascades. The binding of DUSP22 to FAK and the dephosphorylation of FAK are indispensable for DUSP22-meliorated NASH progression. Collectively, our findings identify DUSP22 as a key suppressor of NASH-HCC, and underscore the DUSP22-FAK axis as a promising therapeutic target for treatment of the disease.

## Abstract (web summary)

Mechanisms underlying the pathogenesis of nonalcoholic steatohepatitis are unclear. Here, the authors show that ROS-mediated DUSP22 degradation participates in the progression of fatty liver, contributing to the development of NASH and associated HCC via regulating FAK and its downstream ERK1/2 and NF-κB signaling cascade.

## Introduction

Nonalcoholic steatohepatitis (NASH) usually follows simple hepatic steatosis in nonalcoholic fatty liver disease (NAFLD) that affects up to ~40% of the adult population, and its prevalence is sharply rising in recent decades^1,2^. NASH is becoming a leading pathogenic promoter of end-stage liver diseases, such as HCC and liver failure^3,4^. The pathogenic process of NASH is characterized by hepatic steatosis, hepatocyte ballooning, lobular inflammation, and fibrosis^5^. Furthermore, NASH is commonly accompanied by metabolic abnormalities, including hyperglycemia, obesity, and type 2 diabetes (T2D)^6–8^. However, there are no approved effective pharmaceutic therapies for NASH treatment presently, which is largely attributed to an incomplete understanding of its pathogenesis^9^. Currently, gene therapies such as in vivo and ex vivo gene therapy have become a focus of attention in the regulation of hepatosteatosis. As a vector for in vivo gene delivery, AAV-mediated gene replacement, gene silencing, gene addition, and gene editing have exhibited a great deal of success in preclinical and clinical situations^10^, and AAV-mediated gene overexpression promises to be an effective treatment to mitigate hepatic steatosis and inflammation in NASH^11,12^. Ex vivo gene therapy involves the genetic modification of cells outside of the body to produce therapeutic factors and their subsequent transplantation back into patients or recipient animals^13,14^. We recently reported that ex vivo gene therapy-mediated Trim31 gain-of-function virtually alleviates severe deterioration and progression of steatohepatitis in mice with NAFLD phenotypes^15^. Therefore, a better understanding of NASH pathogenesis and identifying the key therapeutic targets are urgently required.

Several molecular events are activated under metabolic stresses that contribute to hepatic steatosis, such as nuclear factor κB (NF-κB) and mitogen-activated protein kinase (MAPK) signaling, which exhibit multiple functions in cell survival and death, glucose and lipid metabolism, and meta-inflammation^16–18^. Dual-specificity phosphatases (DUSPs), including MAPK phosphatases, mediate the activation of various downstream kinases through serine, threonine, or tyrosine dephosphorylation^19^. DUSP22 (also designated as JKAP or JSP-1), as an atypical DUSP, has a canonical protein-tyrosine phosphatase (PTP) signature motif, *HCXXGXXR*, at residues 87–94. DUSP22 can regulate MAPK signal transduction and is expressed in various types of tissues and cells, demonstrating that DUSP22 may regulate several crucial biological events, such as inflammatory response and tumor cell proliferation^20–22^. DUSP22 has been shown to specifically and positively modulate JNK activation in mammalian cells. Murine embryonic fibroblasts with DUSP22 deficiency fail to mediate JNK activation upon TNF-a or TGF treatment^23^. However, recently increasing studies have reported that DUSP22 also modulates numerous substrates in other signaling cascades^24,25^. For instance, DUSP22 functions as a tyrosine phosphatase to dephosphorylate and inactivate focal adhesion kinase (FAK), which inhibits cell motility^21^. Additionally, DUSP22 directly dephosphorylates and inactivates Lck at Y394 residue, contributing to the repression of T-cell immune response, inflammation, and autoimmune disorders^26^. Due to the wide influences of DUSP22 on MAPKs activation, inflammation- and fibrosis-associated diseases or responses, we hypothesized that there may be a potential functional involvement of DUSP22 in NASH pathogenesis and associated HCC. Nevertheless, supporting data for this hypothesis is largely unknown.

In the present study, we showed that DUSP22 was significantly decreased in fatty liver of NASH individuals and in tumor tissues from NAFLD-HCC patients compared with the normal ones. Hepatocyte-specific DUSP22 knockout markedly hastened the development of NASH and HCC in multiple mouse models by facilitating liver steatosis, inflammation, and fibrosis; however, DUSP22 overexpression in hepatocytes dramatically relieved the severe progression of NASH and related HCC in mice. Mechanistically, intracellular DUSP22 was shown to directly interact with FAK and restrain its phosphorylation at tyrosine residue Y397 and Y576 + Y577, thus suppressing hyperactivation of the ERK1/2 and NF-κB signaling pathways upon metabolic challenges. These results illustrated that DUSP22 is a promising suppressor of NASH and HCC, and maintaining hepatic DUSP22 may serve as a novel therapeutic strategy for the treatment of NASH and fatty liver-associated hepatocarcinogenesis.

## Results

### DUSP22 is downregulated in fatty liver

**Fig. 1:**
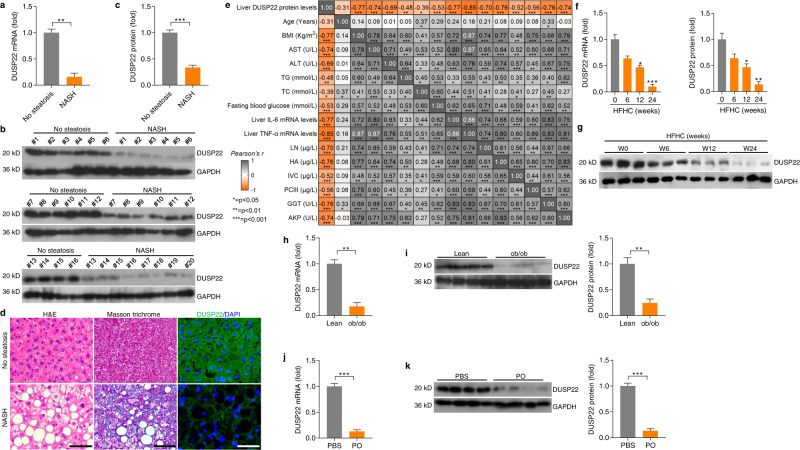
DUSP22 is downregulated in fatty liver. **a** RT-qPCR analysis for DUSP22 mRNA levels in livers of individuals without (No steatosis; *n* = 16) or with NASH (*n* = 20) (**P < 0.01). **b**, **c** Representative western blotting analysis (**b**) and quantification (**c**) of DUSP22 protein expression in livers of individuals without (No steatosis; *n* = 16) or with NASH (*n* = 20) (***P < 0.001)^.^ **d** Liver sections from individuals without (up) or with NASH (down) were stained with H&E (left), Masson trichrome (middle) and immunofluorescence (right) examination for DUSP22 expression (green) (*n* = 8 per group, with 10 images for each sample; Scale bars, 50 µm). **e** Pearson multiple correlation among all parameter indexes gathered from No steatosis and NASH individuals (*n* = 36 per parameter). Orange color shows negative correlation, gray color shows positive color correlation; strong colors tonality identifies strongest correlation. **f**, **g** Analysis of **f** RT-qPCR and **g** western blotting for DUSP22 mRNA and protein levels, respectively, in the livers of WT C57BL/6N mice fed a HFHC at the indicated time of weeks (*n* = 3 per group) (*P < 0.05 and ***P < 0.001 versus the expression at HFHC-0 week group). **h**, **i** Analysis of **h** RT-qPCR and **i** western blotting for DUSP22 mRNA and protein levels, respectively, in the livers from the lean or ob/ob mice (*n* = 4 per group) (**P < 0.01). **j**, **k** Analysis of **j** RT-qPCR and **k** western blotting for DUSP22 mRNA and protein levels, respectively, in the isolated primary hepatocytes followed by BSA or PO treatment (0.4 mM PA and 0.8 mM OA) for 24 h (*n* = 4 per group) (***P < 0.001). Data are expressed as mean ± SEM from at least three independent experiments. For statistical analysis, **a, c, h**–**k** were carried out by two-tailed Student’s *t*-test; **f** and **g** were performed by one-way ANOVA.

Several DUSPs family members have been reported to mediate the progression of NAFLD induced by high-fat diet (HFD)^27–31^. Here in our present work, high-throughput quantitative PCR (HTqPCR) analysis was firstly performed to examine the expression changes of 25 DUSPs family members during the development of pathological NASH in the liver of patients with NASH and in a mouse model of hepatic steatosis induced by high-fat plus high-cholesterol diet (HFHC)^32^. Among all these analyzed DUSPs, DUSP3, DUSP8, DUSP12, DUSP14, DUSP16, DUSP22, and DUSP26 were significantly decreased in the liver of NASH patients with obvious hepatocyte ballooning, severe inflammatory infiltrate, and pattern of liver fibrosis compared with the normal individuals. Similar expression changes of these DUSPs family members were detected in the liver of HFHC-fed mice. More western blotting and RT-qPCR results showed that among these influenced DUSPs, DUSP22 was the most severely affected in NASH patients and mouse models, as proved by its obviously lowest protein and gene expression levels (Supplementary Fig. 1a–d and Fig. 1a–d). Additionally, compared with DUSP3, DUSP8, DUSP12, DUSP14, DUSP16, and DUSP26, hepatic DUSP22 protein expression levels showed a closer negative correlation with the body mass index (BMI), serum total cholesterol (TC), γ-glutamyl Transpeptidase (GGT), fasting blood glucose, alkaline phosphatase (AKP), collagen Type IV (IVC), and laminin (LN) contents, and liver IL-6 gene expression in NASH patients (Supplementary Fig. 2a–f and Supplementary Fig. 3a–h). As expected, multiple linear regression and Pearson multiple correlation analysis confirmed the negative correlation of liver DUSP22 protein expression with NASH severity (Fig. 1e). Consistently, evidently lower DUSP22 mRNA and protein expression levels were observed in the liver of mice fed with HFHC for 24 weeks, and ob/ob mice than that of the mice from normal chow diet (NCD) and lean groups, respectively (Fig. 1f–i). These findings indicated that among all DUSPs family members, DUSP22 reduction was considerably induced in human and murine NASH models.

To establish an in vitro model of lipid accumulation, cells were challenged by PO that consists of palmitic acid (PA) and oleic acid (OA)^33^. We confirmed that mouse primary hepatocytes exhibited remarkably decreased DUSP22 expression upon PO treatment (Fig. 1j and k). To confirm that DUSP22 expression changes were associated with dyslipidemia and inflammation under metabolic stresses, we next constructed a special in vitro model using human hepatocyte L02 cells co-treated with conditional medium (CM) containing serum collected from NASH subjects (NASH Serum) or non-steatosis subjects (Non-steatosis Serum). As expected, CM-containing serum from NASH subjects significantly increased lipid deposition and intracellular triglyceride (TG) contents in L02 cells (Supplementary Fig. 3i–k). Markedly higher levels of pro-inflammatory cytokines and chemokine, including TNF-α, IL-6, IL-18, and MCP-1 were detected in NASH serum than in those from the Non-steatosis group. Importantly, we found that various pro-steatotic stimuli (including TNF-α, IL-6, and LPS) substantially restrained DUSP22 expression (Supplementary Fig. 3l and m).

Given the significant changes in DUSP22 expression in fatty liver, an in vitro model was then established using adenovirus-mediated shDUSP22 knockdown (Ad-shDUSP22) and adenovirus-mediated DUSP22 overexpression (Ad-DUSP22) vectors to initially investigate the regulatory effects of DUSP22 on hepatic steatosis in L02 cells cultured in CM-containing serum from NASH or No-steatosis individuals, and firstly DUSP22 deletion or overexpression had no significant influences on the expression alterations of other DUSPs (Supplementary Fig. 4a–g). We then found that serum from NASH subjects led to lipid and intracellular TG accumulation in L02 cells, which were, however, remarkably accelerated by Ad-shDUSP22, accompanied by aggravated gene expression of pro-inflammatory molecules (Supplementary Fig. 4h–j). Inversely, DUSP22 overexpression considerably ameliorated NASH serum-caused lipid deposition and inflammatory response in L02 cells (Supplementary Fig. 4k–m). NASH patients also exhibited higher TGF-β1 concentrations in serum than the Non-steatosis ones (Supplementary Fig. 4n). Hepatic steatosis stimuli TGF-β1 strongly downregulated DUSP22 expression in L02 cells (Supplementary Fig. 4o), indicating the potential involvement of DUSP22 during fibrosis. We subsequently found that in human hepatic stellate cell (HSC) line LX2, CM derived from NASH serum-treated L02 cells considerably increased the expression of fibrosis markers, including α-SMA, COL1A1, COL3A1, and CTGF, and these effects were markedly exacerbated by Ad-shDUSP22, whereas being dramatically mitigated by Ad-DUSP22 (Supplementary Fig. 4p and q). Furthermore, HTqPCR assay showed that NASH serum-caused expression changes of a total of 128 genes mediating inflammation (87 genes) and lipid metabolism (25 genes) in L02 cells, and fibrosis (16 genes) in LX2 cells were reversed by Ad-DUSP22 (Supplementary Fig. 4r). These in vitro findings initially demonstrated the inhibitory effects of DUSP22 on lipid deposition, inflammation, and fibrosis, thus revealing that DUSP22 may possess therapeutic potential for NASH treatment.

### DUSP22 expression is decreased by ROS production

Because ROS is a potential pro-steatotic mediator and is produced in the liver of animal models with NASH^34,35^, we thus introduced the ROS scavenger N-acetyl-cysteine (NAC) to a mouse model with HFHC-induced NASH. Consistently, a 24-week HFHC feeding caused significant increases in hepatic ROS production, MDA, and H_2_O_2_ contents, which were almost ameliorated by NAC co-administration, along with markedly rescued antioxidant enzyme SOD activities (Supplementary Fig. 5a–e). Notably, HFHC-restrained DUSP22 expression was dramatically restored by NAC (Supplementary Fig. 5f). Pearson correlation analysis further disclosed a negative correlation between DUSP22 expression and H_2_O_2_ contents in the liver of HFHC-fed mice (Supplementary Fig. 5g and h). To examine if ROS production contributes to several key NASH-associated DUSPs reduction during hepatic steatosis, mouse primary hepatocytes were subjected to H_2_O_2_ stimulation. RT-qPCR results demonstrated that DUSP22 was more sensitive to H_2_O_2_ stimulation compared with other DUSPs, exerting an obviously time-dependent manner (Supplementary Fig. 5i). We then found that DUSP22 protein expression levels were gradually decreased from 6 to 48 h of H_2_O_2_ treatment while being remarkably recovered after NAC exposure (Supplementary Fig. 5j and k). As expected, NAC incubation significantly restored DUSP22 expression in PO-incubated primary hepatocytes (Supplementary Fig. 5l). Together, the findings above suggested that ROS production during NASH pathologies was largely responsible for the downregulation of DUSP22.

### Hepatocyte-specific DUSP22 knockout exacerbates HFHC-induced NASH pathologies

**Fig. 2:**
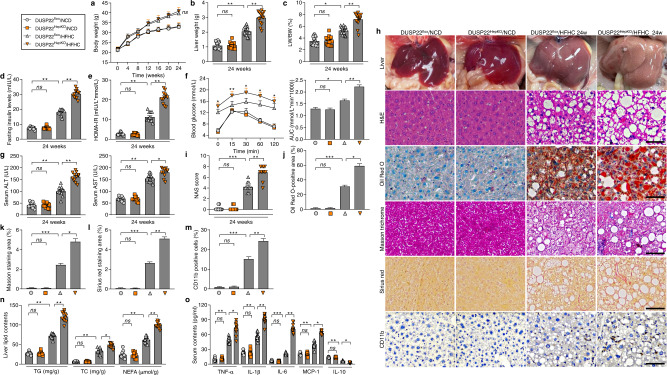
Hepatocyte-specific DUSP22 knockout exacerbates HFHC-induced NASH pathologies. **a**–**e** Measurements of **a** body weight, **b** liver weight, **c** the ratio of liver weight to body weight (LW/BW) (*n* = 15 mice per group), **d** blood fasting insulin levels, and **e** HOMA-IR in DUSP22^flox^ and hepatocyte-specific DUSP22 knockout (DUSP22^HepKO^) mice fed with NCD or a HFHC for 24 weeks (*n* = 12 mice per group) (*P < 0.05 and **P < 0.01; ns no significant difference). **f** Glucose tolerance test (GTT) in DUSP22^flox^ and DUSP22^HepKO^ mice fed with a NCD or a HFHC for 24 weeks were performed, and area under the curve (AUC) values were calculated (*n* = 12 mice per group) (*P < 0.05 and **P < 0.01; ns no significant difference). **g** Serum ALT and AST levels in DUSP22^flox^ and DUSP22^HepKO^ mice fed with a NCD or a HFHC for 24 weeks (*n* = 12 mice per group) (*P < 0.05 and **P < 0.01; ns no significant difference). **h** Representative images of liver appearance, H&E staining, Oil Red O staining, Masson trichrome staining, Sirius red staining and immunohistochemistry examination of CD11b in liver sections from DUSP22^flox^ and DUSP22^HepKO^ mice fed with a NCD or a HFHC for 24 weeks (*n* = 6, 10, or 11 mice per group, with 10 images for each mouse; Scale bars, 50 µm). **i**–**m** Results for **i** NAS score, **j** Oil Red O-positive staining, **k** Masson trichrome-positive staining, **l** Sirius red-positive staining, and **m** CD11b-positive cells were analyzed and quantified (*n* = 6, 10, or 11 mice per group) (*P < 0.05, **P < 0.01, and ***P < 0.001; ns no significant difference). **n** Examination for TG, TC, and NEFA contents in liver of DUSP22^flox^ and DUSP22^HepKO^ mice fed with a NCD or a HFHC for 24 weeks (*n* = 12 mice per group) (*P < 0.05 and **P < 0.01; ns no significant difference). **o** Measurements of serum concentrations of inflammatory factors, including TNF-α, IL-1β, IL-6, MCP-1, and IL-10, in DUSP22^flox^ and DUSP22^HepKO^ mice fed with a NCD or a HFHC for 24 weeks (*n* = 9 or 12 mice per group) (*P < 0.05, **P < 0.01, and ***P < 0.001; ns no significant difference). Data are expressed as mean ± SEM from at least three inde*p*endent experiments^.^ Statistical analysis was carried out by one-way ANOVA.

Due to the potential function of DUSP22 in fatty liver, hepatocyte-specific DUSP22-knockout (DUSP22^HepKO^) mice were then generated. Western blotting results confirmed that DUSP22 knockout did not affect the expression changes of other DUSPs (Supplementary Fig. 6a–f). Under NCD-feeding status beginning from week 0 to week 24, DUSP22^HepKO^ did not develop any NASH-related hepatic phenotype when compared with DUSP22^flox^ mice, as indicated by the similar hepatic histological structures, serum concentrations of ALT and AST, TNF-α, and IL-1β contents, and liver TG and TC levels (Supplementary Fig. 6g–j). Herein, there was no spontaneous hepatic phenotype in the DUSP22^HepKO^ mouse strains. Subsequently, DUSP22^HepKO^ mice were subjected to a 24-week HFHC feeding to investigate whether DUSP22 ablation contributed to hepatic steatosis progression. After HFHC challenge, DUSP22^HepKO^ mice exerted higher liver weight and liver-to-body weight (LW/BW) ratio than that of the DUSP22 ^flox^ group, and no significant difference was observed in the body weight changes between the two groups (Fig. 2a–c). HFHC-fed DUSP22^HepKO^ mice also showed higher fasting blood insulin levels and homeostatic model assessment of insulin resistance (HOMA-IR) values than the DUSP22^flox^ mice (Fig. 2d and e). The glucose tolerance test (GTT) confirmed the decreased glucose tolerance in DUSP22^HepKO^ mice compared with the DUSP22^flox^ ones after HFHC feeding (Fig. 2f). Liver dysfunction caused by HFHC was remarkably accelerated in DUSP22^HepKO^ mice compared to the DUSP22^flox^ group, as proved by the facilitated serum concentrations of ALT and AST (Fig. 2g). Furthermore, after HFHC feeding, DUSP22^HepKO^ mice exhibited severer hepatic steatosis, as evidenced by the intensified liver appearance changes, hepatocellular injury with ballooning and microvesicular steatosis, NAS score by H&E staining, lipid deposition with Oil Red O staining, and typical lobular and pericellular fibrosis by Masson trichrome and Sirius red staining (Fig. 2h–l and Supplementary Fig. 7a). Consistently, DUSP22^HepKO^ mice presented remarkably accelerated hepatic inflammation, as indicated by the evident infiltration of CD11b-positive inflammatory cells compared with the DUSP22^flox^ mice after HFHC feeding (Fig. 2h and m). Moreover, DUSP22^HepKO^-aggravated lipid accumulation and inflammation in HFHC-challenged mice were validated by higher hepatic concentrations of TG, total cholesterol (TC) and nonesterified fatty acids (NEFA), and higher serum contents of pro-inflammatory factors (TNF-α, IL-1β, IL-6, and MCP-1), accompanied with lower concentrations of anti-inflammatory regulator IL-10 (Fig. 2n and o). RT-qPCR results further revealed that abnormal expression of genes associated with inflammation, lipid metabolism, and fibrosis was induced by HFHC feeding in the liver of DUSP22^flox^ mice, and exacerbation of these processes was detected in HFHC-fed DUSP22^HepKO^ mice (Supplementary Fig. 7b–e). Fat accumulation of viscera is generally linked to metabolic disturbance^36^. Actually, no significant difference was detected in the changes in food intake (Supplementary Fig. 8a). Nevertheless, DUSP22^HepKO^ mice exhibited remarkably accelerated body fat weight (BFW), and the ratio of BFW/BW after HFHC feeding compared with the DUSP22^flox^ mice (Supplementary Fig. 8b and c). H&E staining showed that adipocyte size was highly enlarged by HFHC challenge compared with the NCD group; however, hepatocyte DUSP22 expression changes did not influence the adipocyte size of HFHC-fed mice (Supplementary Fig. 8d and e).

We also assayed the primary mouse hepatocytes from DUSP22^flox^ or DUSP22^HepKO^ mice (Supplementary Fig. 9a) and human hepatocytes L02 with or without DUSP22 knockdown. Consistently, DUSP22 knockout showed no significant influences on the expression of other DUSPs (Supplementary Fig. 9b). We confirmed that in response to PO stimulus, primary mouse hepatocytes and L02 cells in the absence of DUSP22 exhibited significantly higher releases of pro-inflammatory cytokines in the collected supernatants than those from the cells with DUSP22 expression (Supplementary Fig. 9c and d). RT-qPCR results verified the function of DUSP22 ablation to aggravate inflammatory response in both PO-incubated primary hepatocytes and L02 cells (Supplementary Fig. 9e and f). In line with in vivo results, DUSP22 deletion caused higher lipid deposition and TG accumulation in primary hepatocytes and L02 cells than those cells with DUSP22 expression upon PO treatment (Supplementary Fig. 9g and h), along with the significantly aberrant expression of lipid metabolism-related genes (Supplementary Fig. 9i and j). Upon TGF-β1 stimulation, human HSCs LX2 produced a higher expression of fibrosis-associated molecules after incubation in CM from PO-treated DUSP22^HepKO^ hepatocytes than those cultured in CM from DUSP22-expressing hepatocytes (Supplementary Fig. 9k). Taken together, all these findings clearly demonstrated that DUSP22 deficiency might accelerate NASH pathologies both in vivo and in vitro.

### Hepatocyte-specific DUSP22 overexpression ameliorates HFHC-caused NASH pathologies

**Fig. 3:**
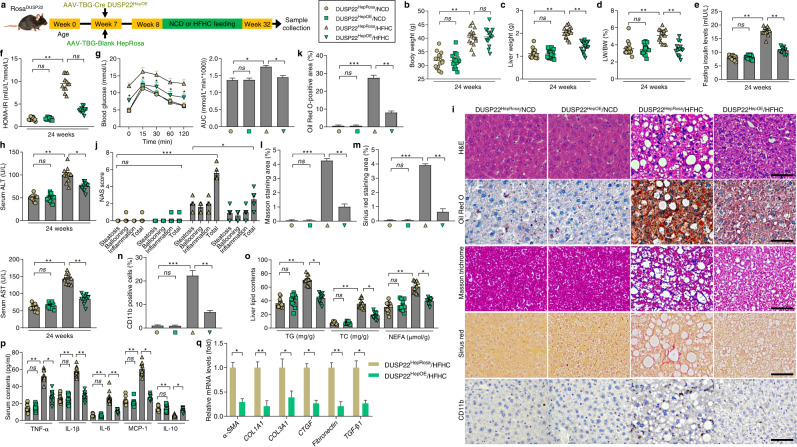
Hepatocyte-specific DUSP22 overexpression ameliorates HFHC-induced NASH pathologies. **a** Schematic diagram of adeno-associated virus (serotype 8)-TBG-Cre (AAV-TBG-Cre)-mediated DUSP22 overexpression in liver of HFHC-fed DUSP22^Rosa^ mice (DUSP22^HepOE^/HFHC). The AAV-TBG-blank was used as control (DUSP22^HepRosa^/HFHC). **b**–**h** Measurements of **b** body weight, **c** liver weight, **d** ratio of LW/BW (*n* = 15 mice per group), **e** blood fasting insulin levels, **f** HOMA-IR, **g** GTT and AUC values, and **h** serum ALT and AST concentrations in DUSP22^HepRosa^ and DUSP22^HepOE^ mice fed with a NCD or a HFHC for 24 weeks (*n* = 12 mice per group) (*P < 0.05 and **P < 0.01; *ns*^,^ no significant difference). **i** Representative images of H&E staining, Oil Red O staining, Masson trichrome staining, Sirius red staining and immunohistochemistry examination of CD11b in liver sections from DUSP22^HepRosa^ and DUSP22^HepOE^ mice fed with a NCD or a HFHC for 24 weeks (*n* = 10 mice per group, with 10 images for each mouse; Scale bars, 50 µm). **j**–**n** Results for **j** NAS score, **k** Oil Red O-positive staining, **l** Masson trichrome-positive staining, **m** Sirius red-positive staining, and **n** CD11b-positive cells were analyzed and quantified (*n* = 10 mice per group) (*P < 0.05, **P < 0.01, and ***P < 0.001; ns no significant difference). **o** Evaluation for TG, TC, and NEFA contents in liver of DUSP22^HepRosa^ and DUSP22^HepOE^ mice fed with a NCD or a HFHC for 24 weeks (*n* = 12 mice per group) (*P < 0.05 and **P < 0.01; ns no significant difference). **p** Calculation of inflammatory factors including TNF-α, IL-1β, IL-6, MCP-1, and IL-10 in serum of DUSP22^HepRosa^ and DUSP22^HepOE^ mice fed with a NCD or a HFHC for 24 weeks (*n* = 12 mice per group) (*P < 0.05 and **P < 0.01; *ns*, no significant difference). **q** RT-qPCR results for mRNA levels of fibrosis-related genes including α-SMA, COL1A1, COL3A1, CTGF, Fibronectin and TGF-β1 in liver of DUSP22^HepRosa^ and DUSP22^HepOE^ mice fed with a HFHC for 24 weeks (*n* = 4 mice per group) (*P < 0.05 and **P < 0.01). Data are expressed as mean ± SEM from at least three independent experiments^.^ For statistical analysis, **b**–**p** were performed by one-way ANOVA; **q** was carried out by two-tailed Student’s *t*-test.

Next, hepatocyte-specific DUSP22 overexpression mice (DUSP22^HepOE^) were created using DUSP22 conditional knock in at the locus of Rosa26 in mice (DUSP22^Rosa^) based on Rosa26 conditional and/or inducible transgenesis to further confirm the regulatory function of DUSP22 in NASH. DUSP22^Rosa^ mice were then injected with adeno-associated virus-serotype 8-thyroxine-binding globulin promoter-Cre recombinase vector (AAV8-TBG-Cre) to induce DUSP22 overexpression only in hepatocytes, and DUSP22^HepRosa^ mice with AAV8-TBG Blank vector injection were used as the control (Supplementary Fig. 10a–d). Also, DUSP22^HepOE^ did not affect the protein expression changes of any other DUSPs (Supplementary Fig. 10e). Fluorescence staining for EGFP, the hepatocyte marker Albumin and HNF4a, the biliary epithelial cell marker CK19, endothelial marker PECAM and smooth muscle cell marker α-SMA on the constructed DUSP22^HepOE^ liver sections confirmed that DUSP22 was efficiently over-expressed in hepatocytes (Supplementary Fig. 10f and g). Examination by H&E staining and biochemical analysis confirmed that DUSP22^HepOE^ mice did not develop a spontaneous hepatic phenotype associated with NASH compared with DUSP22^HepRosa^ mice that were fed a NCD beginning from week 0 to week 24 (Supplementary Fig. 10h–k). After 24-week of HFHC feeding, DUSP22^HepOE^ mice displayed remarkably lower liver weight and LW/BW ratio than those DUSP22^HepRosa^ mice; however, no significant difference was observed in the body weight changes between DUSP22^HepRosa^/HFHC and DUSP22^HepOE^/HFHC groups of mice (Fig. 3a–d). Furthermore, the abnormally enhanced fasting insulin levels, HOMA-IR values, and damaged glucose tolerance caused by HFHC were dramatically attenuated in mice with DUSP22 overexpression (Fig. 3e–g). DUSP22^HepOE^ also meliorated hepatic dysfunction in HFHC-challenged mice, proved by the decreased serum ALT and AST contents (Fig. 3h). Compared with DUSP22^HepRosa^ mice, DUSP22^HepOE^ mice exhibited markedly decreased hepatocyte ballooning, NAS score, lipid deposition, fibrosis and CD11b-positive inflammatory cell infiltration in liver sections following HFHC feeding (Fig. 3i–n). DUSP22^HepOE^ mice also presented considerably decreased hepatic steatosis after HFHC challenge, as evidenced by the decreased liver TC, TG, and NEFA contents, accompanied with amendatory expression levels of genes related to lipid metabolism (Fig. 3o and Supplementary Fig. 11a). Serum and liver inflammatory markers were considerably reversed in DUSP22^HepOE^ mice following HFHC treatment compared with DUSP22^HepRosa^ group (Fig. 3p and Supplementary Fig. 11b). Hepatocyte-specific DUSP22 overexpression remarkably decreased the expression of fibrosis-related genes in liver of HFHC-fed mice (Fig. 3q). HTqPCR results confirmed that DUSP22^HepOE^ alleviated hepatic inflammation, dyslipidemia and fibrosis in response to HFHC feeding (Supplementary Fig. 11c). What’s more, body fat weight, ratio of BFW/BW and adipocytes size were significantly dilated in HFHC-fed mice; DUSP22^HepOE^ markedly decreased the body fat weight and ratio of BFW/BW, but did not influence the changes of adipocytes size in HFHC-challenged mice, as well as the food intake (Supplementary Fig. 12a–e).

To validate the protective function of DUSP22, primary hepatocytes isolated from DUSP22^HepRosa^ and DUSP22^HepOE^ mice and L02 cells transfected with Ad-DUSP22 or Ad-GFP were then used, and DUSP22 overexpression did not affect the expression changes of other DUSPs members (Supplementary Fig. 13a and b). Consistent with in vivo results, PO-triggered lipid deposition and TG accumulation were significantly abolished in murine and human hepatocytes with DUSP22 overexpression, accompanied by meliorative expression levels of lipid metabolism-associated genes (Supplementary Fig. 13c–f). Inflammatory factors released from PO-treated hepatocytes were dramatically abrogated upon DUSP22 overexpression (Supplementary Fig. 13g and h). RT-qPCR results verified the anti-inflammatory effects of DUSP22 in primary hepatocytes and L02 cells with PO stimulation (Supplementary Fig. 13i and j). LX2 cells displayed lower expression of fibrosis-related genes after culture in CM from PO-incubated DUSP22^HepOE^ hepatocytes than those incubated in CM derived from DUSP22^HepRosa^ hepatocytes (Supplementary Fig. 13k). Together, these findings clearly revealed that DUSP22 could protect against steatohepatitis.

### DUSP22 exerts protective effects against HFMCD-induced NASH phenotype

Due to the different mechanisms and pathogenesis of NASH progression caused by a high-fat diet plus methionine- and choline-deficient diet (HFMCD) and other high-energy diets (i.e., HFHC), we subsequently investigated if DUSP22 also exerted protective effects against an 8-week HFMCD-triggered NASH in DUSP22^HepKO^ and DUSP22^HepOE^ mice (Supplementary Fig. 14a and b). HFMCD challenge caused significant decreases in the body weights of DUSP22^flox^ mice, and remarkable hepatic steatosis, as proved by the elevated hepatocyte ballooning, NAS score, lipid deposition, and collagen accumulation. Notably, DUSP22^HepKO^ markedly accelerated these histological alterations in HFMCD-fed mice but had no influence on the body weights (Supplementary Fig. 14c–h). In contrast, DUSP22^HepOE^ clearly ameliorated such hepatic steatosis, as evidenced by the decreased cellular ballooning, NAS score, lipid accumulation, and fibrosis without any influences on the body weight changes (Supplementary Fig. 14i–n). Serum ALT and AST levels, and liver TG, TC, and NEFA contents were markedly accelerated in DUSP^HepKO^ mice fed with HFMCD, but were mitigated in DUSP22^HepOE^ mice (Supplementary Fig. 14o–r). Moreover, HFMCD-increased expression of fibrotic markers was strongly aggravated by DUSP22^HepKO^, whereas DUSP22^HepOE^ mice showed an ameliorated phenotype of these molecules (Supplementary Fig. 14s–v). Furthermore, DUSP22^HepKO^ significantly facilitated inflammatory response in the liver of HFMCD-treated mice, while DUSP22^HepOE^ remarkably alleviated hepatic inflammation (Supplementary Fig. 14w and x). These findings further demonstrated that DUSP22 could blunt HFMCD-induced NASH progression.

### DUSP22 regulates the hepatocyte activation of NF-κB and FAK signaling in HFHC-fed mice

**Fig. 4:**
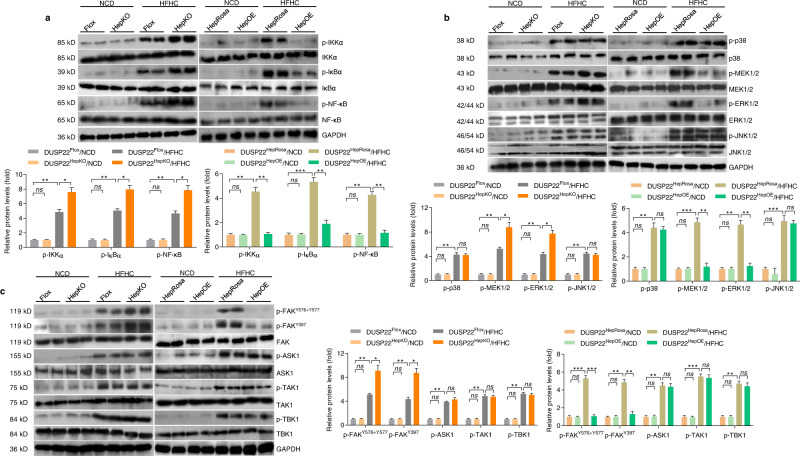
DUSP22 regulates the hepatic activation of NF-κB and FAK signaling in HFHC-fed mice. **a** Representative western blotting and quantification of the protein expression of total and phosphorylated IKKα, IκBα, and NF-κB in liver of DUSP22^flox^, DUSP22^HepKO^, DUSP22^HepRosa^, and DUSP22^HepOE^ mice fed with a NCD or a HFHC for 24 weeks (*n* = 4 mice per group) (*P < 0.05, **P < 0.01, and ***P < 0.001; ns no significant difference). **b** Representative western blotting and quantification of the protein expression of total and phosphorylated p38, MEK1/2, ERK1/2, and JNK1/2 in liver of DUSP22^flox^, DUSP22^HepKO^, DUSP22^HepRosa^, and DUSP22^HepOE^ mice fed with a NCD or a HFHC for 24 weeks (*n* = 4 mice per group) (*P < 0.05, **P < 0.01, and ***P < 0.001; ns no significant difference). **c** Representative western blotting and quantification of the protein expression of total and phosphorylated FAK^Y576+Y577^, FAK^Y397^, ASK1, TAK1, and TBK1 in liver of DUSP22^flox^, DUSP22^HepKO^, DUSP22^HepRosa^, and DUSP22^HepOE^ mice fed with a NCD or a HFHC for 24 weeks (*n* = 4 mice per group) (*P < 0.05, **P < 0.01, and ***P < 0.001; ns no significant difference). Data are expressed as mean ± SEM from at least three independent experiments. Statistical analysis was carried out by two-tailed Student’s *t*-test.

To gain insight into the molecular mechanisms underlying the effects of DUSP22, we examined the downstream pathways involved in the DUSP22-mediated inflammatory response. Given the capacity of DUSP22 to regulate MAPKs activation and the participation of MAPKs signaling in pathological NASH^14,16–19,23,25,37^, we first evaluated the potential involvement of pro-inflammatory NF-κB signaling cascade and MAPKs signaling in fatty liver of DUSP22^HepKO^ and DUSP22^HepOE^ mice after HFHC feeding. Consistent with the changes in inflammatory response observed above, we found that DUSP22^HepKO^ remarkably elevated the HFHC-induced over-activation of the transcription factor NF-κB signaling pathway by facilitating the expression of phosphorylated IKKα, IκBα, and NF-κB/p65, while DUSP22^HepOE^ mice considerably restrained the activation of these proteins (Fig. 4a). The function of DUSP22 to negatively mediate the activation of NF-κB signaling pathway was validated in PO-incubated primary hepatocytes (Supplementary Fig. 15a). We then examined the potential participation of MAPKs pathway. As shown in Fig. 4b and Supplementary Fig. 15b, HFHC feeding or PO exposure markedly activated MAPKs signaling, as indicated by the upregulated phosphorylation of p38, MEK1/2, ERK1/2, and JNK1/2 in liver tissues, and primary hepatocytes. However, DUSP22 deletion further aggravated p-MEK1/2 and p-ERK1/2, but not p-p38 and p-JNK1/2, and meanwhile, DUSP22 overexpression remarkably abolished their activation. The activation of FAK, ASK1, TAK1, and TBK1 plays an essential role in mediating NF-κB and MAPKs signaling pathways in numerous physiological events^12,38–40^ and was then investigated to reveal the possible upstream kinase through which DUSP22 functioned to modulate NF-κB and ERK1/2 signaling pathways. We found that HFHC feeding led to significant increases in the hepatic phosphorylation of FAK^Y576+Y577^, FAK^Y397^, ASK1, TAK1, and TBK1. Notably, DUSP22^HepKO^ only exacerbated the activation of FAK at Y576 + Y577 and Y397, but not affected ASK1, TAK1, and TBK1 activity. These effects were also confirmed in PO-treated primary hepatocytes with DUSP22 ablation. On the contrary, liver tissues and primary hepatocytes from DUSP22^HepOE^ mice exhibited the opposite phenotype after metabolic stresses stimulation (Fig. 4c and Supplementary Fig. 15c). Collectively, these observations illustrated that DUSP22 protected against hepatic steatosis through mediating NF-κB, ERK1/2, and FAK signaling pathways.

### DUSP22 directly interacts with FAK to regulate NASH

**Fig. 5:**
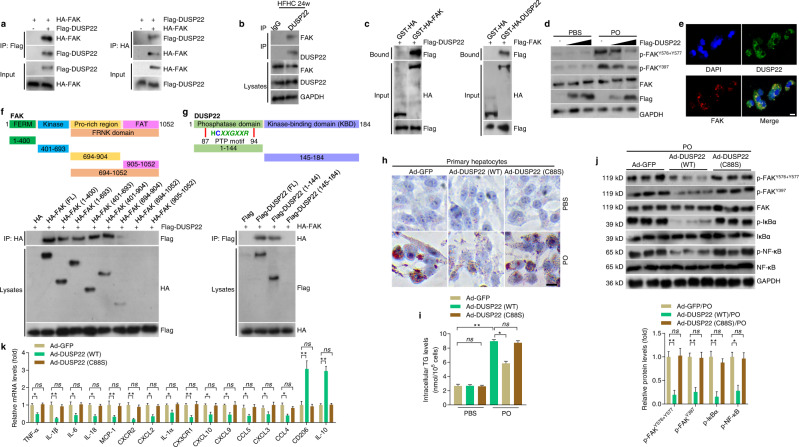
DUSP22 directly interacts with FAK to regulate NASH. **a** Results by Co-IP assays in L02 cells transfected with Flag-tagged DUSP22 and HA-tagged FAK. Anti-Flag and anti-HA antibodies were used as immunoblotting probes. **b** Immunoprecipitation and western blotting analysis indicating the binding of DUSP22 and FAK in liver WT mice after a 24-week HFHC, and IgG was served as a control. **c** GST precipitation analysis presenting the direct DUSP22-FAK binding. Purified GST was used as a control. **d** Representative western blotting of total FAK, p-FAK^Y576+577^, and p-FAK^Y397^ in L02 cells transfected with different amounts of Flag-tagged DUSP22 with PO or BSA treatment for 24 h. **e** Representative immunofluorescent staining of L02 cells co-transfected with Flag-tagged DUSP22 (green) and HA-tagged FAK (red) after transfection for 24 h (*n* = 10 images in total; Scale bar, 10 µm). **f**, **g** Schematic showing full-length (FL) and truncated **f** FAK (top) and **g** DUSP22 (top) with representative Co-IP analysis (bottom) for the mapping analysis of the domains responsible for the DUSP22-FAK interaction in L02 cells. **h**, **i** Representative Oil red O staining images (**h**) and intracellular TG contents (**i**) indicating the lipid deposition in PO-treated Ad-DUSP22 (WT) or Ad-DUSP22 (C88S)-transfected primary hepatocytes for 24 h. primary hepatocytes transfected with Ad-GFP were used as a control (*n* = 5 per group, with 10 images per group; Scale bar, 25 µm) (*P < 0.05 and **P < 0.01; ns no significant difference). **j** Representative western blotting of phosphorylated and total total FAK, IκBα, and NF-κB in primary hepatocytes transfected with GFP, WT DUSP22 or the C88S DUSP22 variant at 24 h after PO treatment (*n* = 3 per group) (*P < 0.05 and **P < 0.01; ns no significant difference). **k** RT-qPCR analysis showing inflammation-related crucial genes expression changes in PO-treated Ad-DUSP22 (WT) or Ad-DUSP22 (C88S)-transfected primary hepatocytes for 24 h (*n* = 6 per group) (*P < 0.05 and **P < 0.01; ns no significant difference). Data are expressed as mean ± SEM from at least three independent experiments. For statistical analysis, **i**–**k** were performed by two-tailed Student’s *t*-test.

Accordingly, the molecular link between DUSP22 and FAK was mainly explored. Co-immunoprecipitation (co-IP) results indicated the interaction between DUSP22 and FAK (Fig. 5a), which was confirmed in liver tissues of mice with a 24-week HFHC feeding (Fig. 5b). Glutathione S-transferase (GST) precipitation analysis confirmed a direct interaction between DUSP22 and FAK (Fig. 5c), and the suppressive effects of DUSP22 on FAK activation at Y576 + Y577 and Y397 sites were dose-dependent (Fig. 5d). Furthermore, double immunofluorescence staining indicated a considerable degree of the colocalization of DUSP22 and FAK in L02 cells (Fig. 5e). Molecular mapping with the truncated FAK and DUSP22 suggested that the FERM (band 4.1, ezrin, radixin, moesin homology; residues 1–400) and Kinase (residues 401–693) domains were the main fragments of FAK responsible for binding to DUSP22, and the FAK-binding fragment in DUSP22 was mainly located in the phosphatase domain (residues 1–144) (Fig. 5f and g).

Additionally, we found that NASH patients exhibited significantly elevated activation of FAK, indicated by the upregulated expression of p-FAK^Y576+Y577^ and p-FAK^Y397^ in liver specimens compared with the Non-steatosis participants (Supplementary Fig. 16a). Gradually increased phosphorylation of FAK^Y576+Y577^ and FAK^Y397^ was further detected in the liver of HFHC-challenged mice (Supplementary Fig. 16b). A negative correlation between DUSP22 expression and the activation of FAK was confirmed in NASH subjects (Supplementary Fig. 16c and d). Double immunofluorescence validated the colocalization of DUSP22 and the activated FAK in liver sections of NASH patients (Supplementary Fig. 16e and f). Possible correlation between DUSP22 and FAK for both human and mouse, in theory, was predicted by GeneMANIA and String (Supplementary Fig. 16g and Supplementary Fig. 17a). The interaction between DUSP22 and FAK, and their co-expression in mouse primary hepatocytes were validated by Co-IP and immunofluorescence staining (Supplementary Fig. 17b and c). We further found that introduction of FAK-FERM and FAK-Kinase variants but not FAK-related non-kinase (FRNK) variant in FAK failed to restore the activation of FAK and the down-streaming ERK1/2 and NF-κB/p65 signal, accompanied with the regained pro-inflammatory response, lipid deposition and intracellular TG accumulation with dysregulated gene expression related to lipid metabolism upon PO exposure in FAK-knockout L02 cells (Supplementary Fig. 18a–e). Together, these findings demonstrated that DUSP22 directly interacted with FAK and inhibited FAK activation mainly at Y576 + Y577 and Y397, which was essential for the anti-NASH effects of DUSP22 both in vivo and in vitro.

It is known that C88S mutant (DUSP22-C88S) is a dominant-negative mutant without the capacity to dephosphorylate substrates^22,26,41^. Hepatocytes were infected with an adenoviral vector expressing a full-length DUSP22, or a mutant DUSP22 with cysteine 88 substituted by serine (C88S) to further determine whether the interaction of DUSP22 and FAK was indispensable for DUSP22 to perform its biological functions in PO-treated cells. We noted that C88S mutant remarkably abrogated the protective effects of DUSP22 to restrain lipid deposition and TG accumulation in mouse primary hepatocytes and L02 cells upon PO stimulation (Fig. 5h and i, and Supplementary Fig. 19a–c). Moreover, after PO incubation, the inhibitory role of DUSP22 on the phosphorylation of FAK^Y576+Y577^, FAK^Y397^, IκBα, and NF-κB/p65 was considerably eliminated in primary hepatocytes and L02 cells with C88S mutant, accompanied by the regained pro-inflammatory response (Fig. 5j and k, and Supplementary Fig. 19d and e). The expression of DUSP22 considerably ameliorated PO-stimulated dyslipidemia in hepatocytes, but this effect was completely abolished when DUSP22-C88S was mutated (Supplementary Fig. 19f and g). As expected, LX2 cells exhibited markedly restored expression of fibrosis-associated genes after culture in CM from PO-incubated mouse hepatocytes with DUSP22-C88S mutation (Supplementary Fig. 19 h).

Given the close correlation between FAK blockage with the activity of PTP-motif domain containing C88S in DUSP22, an AAV8-TBG-loaded WT full-length DUSP22 sequence vector (AAV-TBG-DUSP22) and AAV8-TBG-loaded DUSP22 with PTP-motif domain deletion vector (AAV-TBG-DUSP22 (ΔPTP motif)) were produced and introduced to further examine the effects of DUSP22 with PTP-motif mutants on HFHC-caused NASH pathologies in vivo (Supplementary Fig. 20a). The DUSP22^HepKO^ mice were injected with AAV-TBG-DUSP22 (Hep^DUSP22^GOF) or AAV-TBG-DUSP22 (ΔPTP motif) (Hep^DUSP22-ΔPTP motif^GOF) to induce corresponding DUSP22 gain-of-function (GOF) expression, followed by HFHC feeding for 24 weeks. The DUSP22^HepKO^ mice injected with AAV-TBG-Blank vectors were used as controls (HepControl). As expected, mice injected with AAV-TBG-DUSP22 (ΔPTP motif) failed to reduce HFHC-increased liver weight, the ratio of LW/BW, fasting insulin levels, HOMA-IR values, and serum concentrations of ALT and AST (Supplementary Fig. 20b–f). Meanwhile, ΔPTP-motif ablation remarkably abolished the protective function of DUSP22 on NASH in mice, as proved by the restrengthened hepatocyte ballooning, NAS score, lipid deposition, collagen accumulation, and CD11b-positive inflammatory cell infiltration in the liver of HFHC-fed mice (Supplementary Fig. 20g–l). Consistently, DUSP22-ΔPTP-motif mutations failed to reduce hepatic lipid accumulation and serum concentrations of pro-inflammatory factors, along with the intensively dysregulated gene expression levels associated with inflammation via aggravating FAK activation and its downstream cascades (p-ERK1/2, p-IκBα, and p-NF-κB), lipid metabolism and fibrosis (Supplementary Fig. 20m–r). These results revealed that DUSP22 directly interacted with FAK to subsequently mitigate NASH pathologies via the activation of PTP motif.

### FAK inactivation is essential for DUSP22 function

**Fig. 6:**
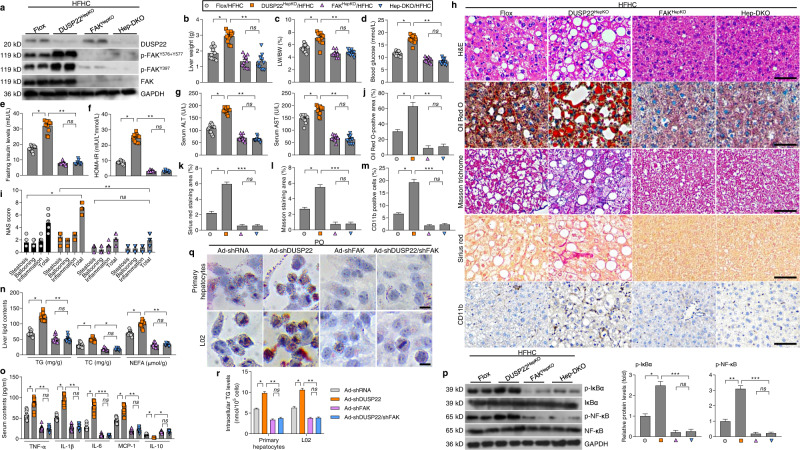
FAK inactivation id essential for DUSP22 function. **a** Representative western blotting for the expression of DUSP22, p-FAK^Y576+Y577^, p-FAK^Y397^, and FAK in the livers from the DUSP22^HepKO^ mice, hepatocyte-specific FAK-knockout (FAK^HepKO^) mice, hepatocyte-specific DUSP22 and FAK double knockout (Hep-DKO) mice and Flox mice after a 24-week HFHC feeding (*n* = 4 mice per group). **b**–**g** Measurements of **b** liver weight, **c** ratio of LW/BW, **d** blood glucose levels, **e** fasting insulin levels, **f** HOMA-IR, and **g** serum concentrations of ALT and AST from the indicated groups of mice fed with HFHC for 24 weeks (*n* = 12 mice per group) (*P < 0.05 and **P < 0.01; ns no significant difference). **h** Representative images of H&E staining, Oil Red O staining, Masson trichrome staining, Sirius red staining and immunohistochemistry examination of CD11b in liver sections from the shown groups of mice fed with a HFHC for 24 weeks (*n* = 6 or 12 mice per group, with 10 images for each mouse; Scale bars, 50 µm). **i**–**m** Results for **i** NAS score, **j** Oil Red O-positive staining, **k** Sirius red-positive staining, **l** Masson trichrome-positive staining, and **m** CD11b-positive cells were analyzed and quantified (*n* = 6 or 12 mice per group) (*P < 0.05, **P < 0.01, and ***P < 0.001; *ns*, no significant difference). **n** Assessments for TG, TC, and NEFA contents in liver of the shown groups of mice fed a HFHC for 24 weeks (*n* = 12 mice per group) (*P < 0.05 and **P < 0.01; ns no significant difference). **o** Calculation of serum levels of inflammatory factors, including TNF-α, IL-1β, IL-6, MCP-1, and IL-10, in mice from the indicated groups after a 24-week HFHC feeding (*n* = 12 mice per group) (*P < 0.05, **P < 0.01, and ***P < 0.001; ns no significant difference). **p** Representative western blotting and quantification of the protein expression of total and phosphorylated IκBα and NF-κB in liver from the shown groups of mice fed with a HFHC for 24 weeks (*n* = 4 mice per group) (*P < 0.05, **P < 0.01, and ***P < 0.001; ns no significant difference). **q**, **r** Representative Oil red O staining images (**q**) and intracellular TG contents (**r**) in 24 h PO (0.4 mM PA and 0.8 mM OA)-treated primary hepatocytes or L02 cells transfected or co-transfected with Ad-shDUSP22, Ad-shFAK, and Ad-shDUSP22/shFAK. Cells transfected with Ad-shRNA were used as a control (*n* = 5 per group, with 10 images per group; Scale bar, 25 µm) (*P < 0.05 and **P < 0.01; ns no significant difference). Data are expressed as mean ± SEM from at least three independent experiments. Statistical analysis were performed by two-tailed Student’s *t*-test.

Given the crucial of FAK activation in NASH progression and to further confirm the potential suppressive effects of DUSP22 on FAK signaling in vivo, hepatocyte-specific FAK knockout (FAK^HepKO^) (Supplementary Fig. 21a–c), and hepatocyte-specific DUSP22 and FAK double deletion (Hep-DKO) mice were thereafter generated (Supplementary Fig. 21d). Depletion of DUSP22 and FAK in the liver in these mouse lines was confirmed through western blot analysis (Fig. 6a), and FAK deficiency abrogated the potentiating effect of DUSP22 knockout on HFHC-induced phosphorylation of FAK^Y576+Y577^ and FAK^Y397^. Intriguingly, DUSP22^HepKO^-accelerated liver weight, LW/BW ratio, blood glucose levels, fasting insulin contents, HOMA-IR values, and serum concentrations of ALT and AST were considerably meliorated in mice with FAK deletion after HFHC challenge (Fig. 6b–g). FAK knockout also diminished the potentiating effects of DUSP22^HepKO^ on HFHC-enhanced NAS score, lipid deposition, fibrosis formation, and CD11b-positive inflammatory cell infiltration (Fig. 6h–m), accompanied by the dramatically reduced hepatic TG, TC, and NEFA levels and serum pro-inflammatory factors with lower phosphorylation of IκBα and NF-κB/p65 in the liver (Fig. 6n–p). Consistently, FAK knockdown abolished the function of Ad-shDUSP22 to exacerbate PO-stimulated activation of FAK^Y576+Y577^ and FAK^Y397^ (Supplementary Fig. 21e), lipid deposition, intracellular TG generation, and p-IκBα and p-NF-κB/p65 in both primary hepatocytes and L02 cells (Fig. 6q and r, and Supplementary Fig. 21f).

To drastically confirm the function of DUSP22/FAK axis on NASH pathologies, we further established another special NASH model in vivo. FAK^HepKO^ mice and FAK^flox^ mice were injected with/without LV-shDUSP22 vector through the portal vein to construct hepatocyte-specific DUSP22-FAK loss-of-function mice or hepatocyte-specific FAK loss-of-function mice before 24 weeks of NASH diet feeding (FAK^HepKO^/HFHC) or halfway by NASH diet feeding (FAK^HepKO^/LV-shDUSP22/HFHC, FAK^flox^/LV-shDUSP22/HFHC) (Supplementary Fig. 22a). Phosphorylation of FAK^Y576+Y577^ and FAK^Y397^ was obviously detected in the liver of FAK^flox^/LV-shDUSP22/HFHC mice, but not in FAK^HepKO^/HFHC and FAK^HepKO^/LV-shDUSP22/HFHC mice (Supplementary Fig. 22b). We found that compared with HFHC-challenged FAK^flox^/LV-shDUSP22 mice, the phenotypes indicating NASH progression including liver weight, the ratio of LW/BW, blood glucose levels, fasting insulin contents, HOMA-IR index, and serum concentrations of ALT and AST were considerably mitigated in LV-shDUSP22 mice with FAK^HepKO^ although no significant difference was detected in the body weight changes (Supplementary Fig. 22c–i). As expected, FAK^HepKO^/LV-shDUSP22 mice exhibited meliorated hepatocyte ballooning, NAS score, lipid deposition, fibrosis, CD11b-positive inflammatory cell infiltration in the liver than that of FAK^flox^/LV-shDUSP22 mice after HFHC feeding (Supplementary Fig. 22j–o). Furthermore, HFHC-caused abnormal liver TG, TC, and NEFA contents, as well as the mRNA expression levels of genes associated with lipid metabolism and fibrosis, were effectively prohibited in FAK^HepKO^/LV-shDUSP22 mice compared with the FAK^flox^/LV-shDUSP22 mice (Supplementary Fig. 22p and q). Additionally, FAK^HepKO^/LV-shDUSP22 mice also showed markedly decreased expression of p-IκBα and p-NF-κB/p65 in the liver, accompanied by the ameliorated pro-inflammatory factors in serum and/or liver following HFHC challenge (Supplementary Fig. 22r–t). These results demonstrated that FAK inactivation was essential for DUSP22 to perform its protective function against NASH progression.

### DUSP22 protects against HFHC-induced NASH pathologies

**Fig. 7:**
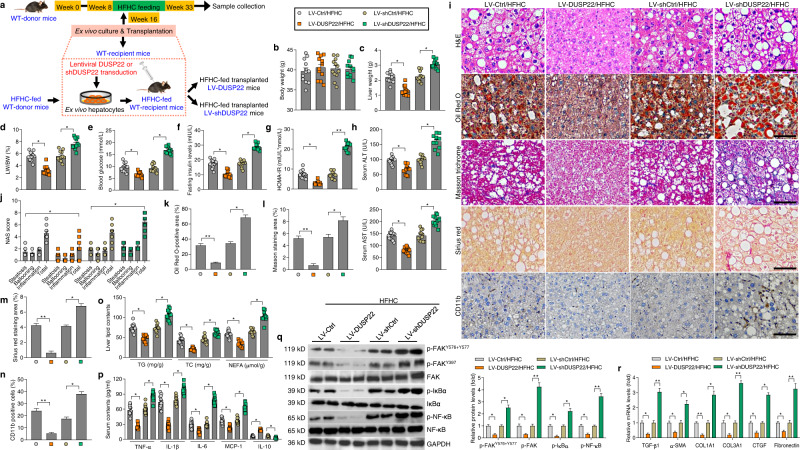
DUSP22 protects against HFHC-induced NASH pathologies. **a** Scheme showing the ex vivo-regulated gene therapy. Primary hepatocytes from the preconditioned WT mice fed with an 8-week HFHC as donor were isolated and ex vivo cultured. The cultured hepatocytes were then transduced with lentivirus-loaded DUSP22 or shDUSP22 sequences. The corresponding blank vector were used as controls. Subsequently, the additional HFHC-fed littermates mice as recipient were injected with the transduced hepatocytes through portal vein. The HFHC-fed transplanted mice (HFHC LV-DUSP22 or HFHC LV-shDUSP22) were thereafter fed with a HFHC diet for an additional 16 weeks. **b**–**h** Measurements of **b** body weight, **c** liver weight, **d** ratio of LW/BW, **e** blood glucose levels, **f** fasting insulin levels, **g** HOMA-IR index, and **h** serum ALT and AST (*n* = 12 mice per group) (*P < 0.05 and **P < 0.01). **i** Representative images of H&E staining, Oil Red O staining, Masson trichrome staining, Sirius red staining and immunohistochemistry examination of CD11b in liver sections from the shown groups of mice (*n* = 6 or 12 mice per group, with 10 images for each mouse; Scale bars, 50 µm). **j**–**n** Results for **j** NAS score, **k** Oil Red O-positive staining, **l** Masson trichrome-positive staining, **m** Sirius red-positive staining, and **n** CD11b-positive cells were analyzed and quantified (*n* = 6 or 12 mice per group) (*P < 0.05 and **P < 0.01). **o** Examination of TG, TC, and NEFA in hepatic tissues from the indicated groups of mice (*n* = 12 mice per group) (*P < 0.05). **p** Assessments of serum TNF-α, IL-1β, IL-6, MCP-1, and IL-10 contents from the shown groups of mice (*n* = 11 mice per group) (*P < 0.05). **q** Representative western blotting and quantification of the protein expression of total and phosphorylated FAK^Y576+Y577^, FAK^Y397^, IκBα, and NF-κB in liver from the shown groups of mice (*n* = 4 mice per group) (*P < 0.05 and **P < 0.01). **r** RT-qPCR results for mRNA levels of fibrosis-related genes including TGF-β1, α-SMA, COL1A1, COL3A1, CTGF, and Fibronectin in liver of the indicated groups of mice (*n* = 8 mice per group) (*P < 0.05 and **P < 0.01). Data are expressed as mean ± SEM from at least three independent ex*p*eriments. Statistical analysis were performed by two-tailed Student’s *t*-test.

The ex vivo gene therapy method using lentivirus-loaded full-length DUSP22 sequences (LV-DUSP22) or shRNA targeting DUSP22 (LV-shDUSP22) was subsequently utilized to disclose the therapeutic potential of DUSP22 for NASH management (Fig. 7a). DUSP22 overexpression or knockdown in liver tissues was validated by western blotting (Supplementary Fig. 23a and b). In the ex vivo transplanted mouse model, hepatocyte-specific DUSP22 overexpression (LV-DUSP22) transduction dramatically reduced liver weight, LW/BW ratio, blood glucose concentrations, fasting insulin levels, HOMA-IR values, and serum concentrations of ALT and AST in mice with a 24-week HFHC challenge; however, hepatocyte-specific DUSP22 knockdown (LV-shDUSP22) mice exhibited remarkably accelerated NASH phenotypes caused by HFHC, while no significant difference was observed in the body weight changes of all groups of mice (Fig. 7b–h). LV-DUSP22/HFHC mice also exerted prominent decreases in hepatocyte ballooning, NAS score, lipid deposition, fibrosis, and inflammatory cell infiltration in liver tissues, accompanied by reduced hepatic TG, TC and NEFA contents, and serum pro-inflammatory mediators (Fig. 7i–o). Moreover, the enhanced activation of FAK, IκBα, and NF-κB/p65, and abnormal expression of fibrosis-, inflammation- and lipid metabolism-related genes were also considerably diminished in LV-DUSP22/HFHC mice compared with the LV-Ctrl/HFHC mice. Nevertheless, LV-shDUSP22 mice exhibited more severe liver steatosis than that those phenotypes in LV-shCtrl mice after HFHC feeding (Fig. 7p–r and Supplementary Fig. 23c and d). These results illustrated that HFHC-triggered NASH progression can be efficaciously relieved by DUSP22-regulated ex vivo gene therapy.

### DUSP22 ameliorates NASH-associated HCC

**Fig. 8:**
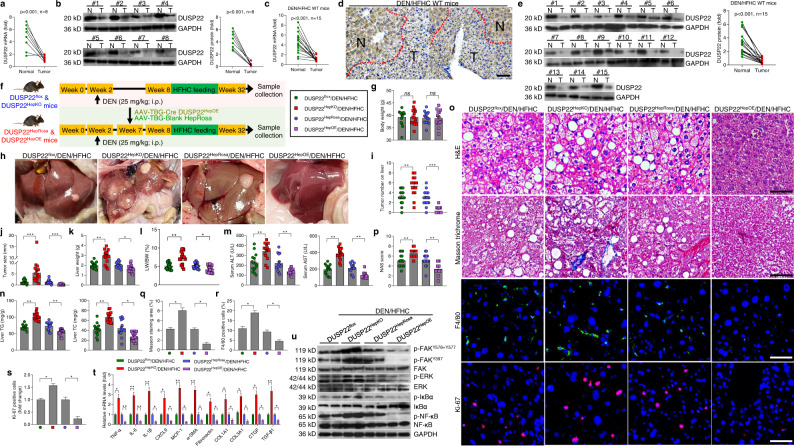
DUSP22 ameliorates NASH-associated HCC. **a**, **b** Results for **a** RT-qPCR and **b** western blotting analysis of DUSP22 mRNA and protein expression levels, respectively, in 8 individual paired NAFLD-HCC (T) and adjacent normal samples (N). **c**–**e** Results for **c** RT-qPCR, **d** immunohistochemistry examination, and **e** western blotting analysis of DUSP22 expression in dietary NASH-HCC mouse models: DEN-injected and HFHC-treated WT mice (*n* = 15 mice per group). **f** Scheme for the experimental design on DEN-injected and HFHC-induced NASH-HCC mouse model with hepatocyte-specific DUSP22 knockout (top) or hepatocyte-specific DUSP22 overexpressin (bottom). At the age of 14 days, DUSP22^flox^, DUSP22^HepKO^, DUSP22^HepRosa^, and DUSP22^HepOE^ mice were injected with a single dose of DEN. Starting at 8 weeks of age, mice were fed with HFHC diet for an additional 24-week. **g** Records of body weight of mice from the shown groups of mice (*n* = 15 mice per group) (ns no significant difference). **h** Representative images of liver appearances (*n* = 8 mice per group). **i**, **j** Surface tumor number (**i**) and tumor size (**j**) in each shown group were quantified (*n* = 15 mice per group) (**P < 0.01 and ***P < 0.001)^.^ **k**–**n** Measurements of **k** liver weight, **l** ratio of LW/BW, **m** serum concentrations of ALT and AST, and **n** liver TG and TC levels from the indicated groups of mice (*n* = 15 mice per group) (*P < 0.05 and **P < 0.01). **o** Representative images of H&E staining (Scale bars, 50 µm), Masson trichrome staining (Scale bars, 50 µm), immunofluorescence examination of F4/80 (Scale bars, 20 µm) and Ki-67 (Scale bars, 20 µm) in liver sections from the shown groups of mice (*n* = 6 or 15 mice per group, with 10 images for each mouse). **p**–**s** Measurements of **p** NAS score, **q** Masson trichrome-positive staining, **r** F4/80-positive cells, and **s** Ki-67-positive cells were analyzed and quantified (*n* = 6 or 15 mice per group) (*P < 0.05 and **P < 0.01). **t** RT-qPCR results for mRNA levels of inflammation- and fibrosis-related genes as shown in liver of the indicated groups of mice (*n* = 8 mice per group) (*P < 0.05 and **P < 0.01). **u** Representative western blotting and quantification of the protein expression of total and phosphorylated FAK^Y576+Y577^, FAK^Y397^, ERK1/2, IκBα, and NF-κB in liver of the indicated groups of mice (*n* = 4 per group). Data are expressed as mean ± SEM from at least three independent experiments. Statistical analysis were performed by two-tailed Student’s *t*-test.

DUSP22 has been reported to function as a tumor suppressor^21,22^. To probe whether DUSP22 was involved in NAFLD-HCC progression, DUSP22 expression levels were subsequently examined in 8-paired human NAFLD-HCC tumor samples and the paired adjacent normal tissues. DUSP22 mRNA and protein expression levels were also significantly downregulated in NAFLD-HCC specimens compared to the adjacent normal ones (Fig. 8a and b). We then examined the expression of DUSP22 in two obesity-associated NAFLD-HCC mouse models. Similarly, DUSP22 was remarkably decreased in all HCC tumors from *N,N*-diethylnitrosamine (DEN), and HFHC-treated mice (Fig. 8c–e). Consistently, markedly reduced DUSP22 expression was observed in HCC tumors from DEN-treated ob/ob mice (Supplementary Fig. 24a and b). Additionally, HCC cell lines (Hep3B, HepG2, and SMMC-7721) also exerted significantly decreased DUSP22 expression compared to the non-tumor cell line (Supplementary Fig. 24c and d). These results elucidated that DUSP22 downregulation might contribute to NAFLD-associated HCC development.

We subsequently investigated the regulatory role of DUSP22 on the pathological progression of NASH to HCC in DUSP22^HepKO^ and DUSP22^HepOE^ mice treated with DEN (25 mg/kg; at 2 weeks old) and a 24-week HFHC diet, and DUSP22^flox^ and DUSP22^HepRosa^ mice were served as corresponding controls, respectively (Fig. 8f). No significant difference was detected in the body weight changes among all groups of mice (Fig. 8g). As shown in Fig. 8h–j, DUSP22^HepKO^ mice developed more number and larger size of tumors on the liver surface compared with the DUSP22^flox^ mice after DEN/HFHC treatment. DUSP22^HepKO^ mice also exerted higher liver weight, LW/BW ratio, serum concentrations of ALT and AST, and liver TG and TC levels than that of the DUSP22^flox^ mice in response to DEN/HFHC challenge (Fig. 8k–n). On the contrary, hepatic-specific DUSP22 overexpression considerably restrained NASH-associated HCC tumorigenesis in DEN/HFHC-treated mice with ameliorated NASH phenotypes (Fig. 8h–n). Notably, besides its regulation of tumorigenesis, DUSP22^HepOE^ dramatically repressed the pathological features of NASH, including hepatic steatosis, fibrosis, F4/80-mediated inflammatory cell infiltration, and inhibited HCC cell proliferation proved by the decreased Ki-67-positive staining compared to the DUSP22^HepRosa^ mice post DEN/HFHC treatment, accompanied with the reduced expression of inflammation- and fibrosis-related genes via the blockage of its downstream FAK, ERK1/2, and NF-κB signaling pathways. However, these DEN/HFHC-induced histological and mechanistic hallmarks for NASH-related HCC were significantly accelerated in DUSP22^HepKO^ mice compared with DUSP22^flox^/DEN/HFHC mice (Fig. 8o–u). Additionally, in vitro results using HepG2 and SMMC-7721 cells transfected with Ad-shDUSP22 or Ad-DUSP22 showed that DUSP22 knockdown significantly facilitated the cell proliferation, while its overexpression markedly suppressed HCC cell growth compared with corresponding control groups by CCK-8 and EdU staining (Supplementary Fig. 24e–i). TUNEL staining confirmed that promoting DUSP22 led to apoptosis in HCC cells (Supplementary Fig. 24j and k). Consistently, SMMC-7721 cells with Ad-shDUSP22 showed clearly elevated phosphorylation of FAK^Y576+Y577^, FAK^Y397^, ERK1/2, IκBα, and NF-κB, which were, however, diminished in HepG2 cells transfected with Ad-DUSP22 (Supplementary Fig. 24l). All these results illustrated that DUSP22 suppresses NAFLD-associated HCC both in vivo and in vitro.

FAK has been reported to function as an oncogene, including HCC^42,43^. To further explore whether FAK blockage was involved in DUSP22-restrained NASH-HCC progression, its activation was then measured in the liver of NAFLD-HCC patients. Western blotting results showed that NAFLD-HCC patients exhibited significantly elevated expression of p-FAK^Y576+Y577^ and p-FAK^Y397^ compared with the matched adjacent normal samples (Supplementary Fig. 25a). Subsequently, NASH-HCC murine model was established using DUSP22^HepKO^, FAK^HepKO^, and Hep-DKO mice to investigate the effects of FAK activation on DUSP22-mediated NASH-HCC progression. As displayed in Supplementary Fig. 25b–d, DUSP22^HepKO^-accelerated NASH-HCC was considerably mitigated in DEN/HFHC-fed mice from FAK^HepKO^ and Hep-DKO groups, as proved by the decreased tumor number and size. The number of cells with Ki-67 positive staining aggravated by DUSP22^HepKO^ was also significantly abolished upon FAK knockout (Supplementary Fig. 25e and f). H&E staining indicated that after DEN/HFHC challenge, DUSP22^HepKO^ mice developed severer cytological features of cancerous cells distributed in parenchyma showing an abnormal hepatic architecture, while being ameliorated in FAK^HepKO^ mice or Hep-DKO mice, along with markedly decreased serum concentrations of ALT and AST (Supplementary Fig. 25 g and h), indicating the improved hepatic functions. Findings in this regard illustrated that DUSP22-inhibited progression of NASH-HCC was partially attributed to the blockage of FAK signaling.

## Discussion

Currently, there are no well-recognized and approved pharmacological therapies for NASH treatment except lifestyle approaches, and the mechanisms that contribute to HCC development in NASH still remain unclear. Developing effective strategies for NASH management largely relies on the target of the key regulators in its pathogenic pathways. Recent studies on the interaction between genetic and environmental factors on fatty liver show that metabolic disorders can significantly amplify the effects of gene variants on NASH, from steatosis to hepatic inflammation and cirrhosis^11,12,14,15^. Preliminary clinical trials have indicated that NASH patients with specific gene variants respond differently to lifestyle and drug intervention^44^. Herein, more studies on the treatment of NASH in specific gene variants are urgently required, which may be helpful to guide personalized treatment in the near future. Emerging studies have reported that gene therapy approaches are of potential for the treatments of hepatic steatosis and its related HCC^10–15,45,46^.

**Fig. 9:**
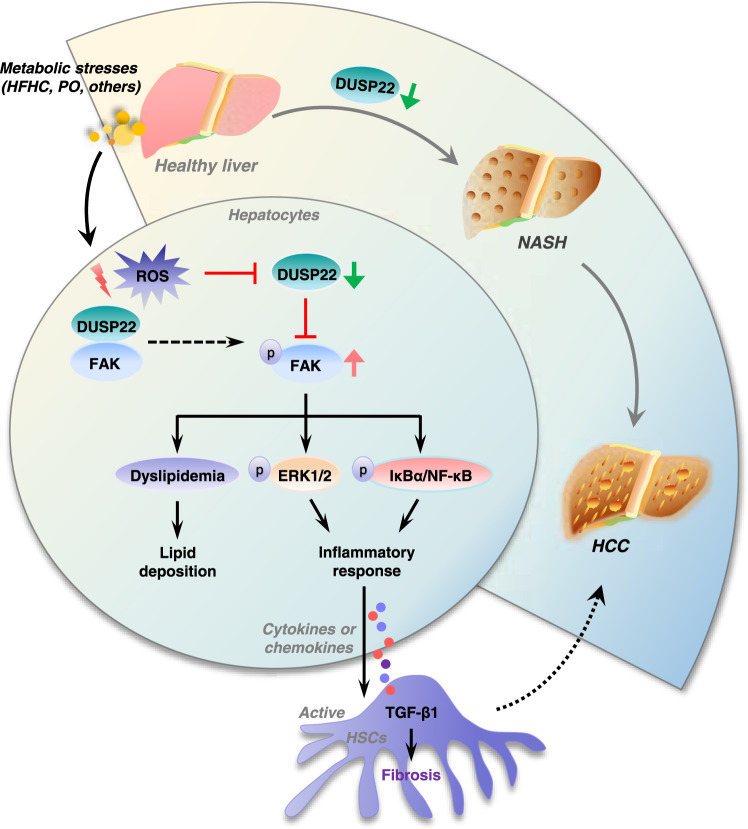
Schematic diagram showing the mechanism of action of DUSP22 in NASH-HCC. Excessive metabolic stimuli, such as HFHC and PO, promotes ROS generation, which leads to DUSP22 downregulation, thereafter contributing to the activation of FAK signaling through phosphorylating FAK at Y397 and Y576 + Y577 residues. FAK activation results in lipid deposition and inflammatory response via activating ERK1/2 and NF-κB signaling pathways. Inflammatory factors released from hepatocytes facilitates hepatic fibrosis. All these effects mediated by DUSP22/FAK axis contribute to the progression of NASH and NASH-associated HCC.

In the present study, we at first identified DUSP22 as a critical suppressor of steatohepatitis and NAFLD-associated HCC (Fig. 9). Using multiple mouse models with NASH induced by long-term HFHC or HFMCD feeding, we found that hepatocyte-specific DUSP22 ablation (DUSP22^HepKO^) using CRISPR/Cas9 system prominently exacerbated hepatic steatosis, inflammation, and fibrosis; however, AAV8-mediated DUSP22 overexpression (DUSP22^HepOE^) considerably conferred protection against NASH progression after HFHC or HFMCD challenge, which was confirmed by lentivirus-mediated DUSP22 (LV-DUSP22) ex vivo gene therapy. Meanwhile, DUSP22 deletion or promotion did not influence the expression of other DUSPs, and mice with DUSP22^HepKO^ or DUSP22^HepOE^ did not develop any NASH-associated spontaneous hepatic phenotype under normal conditions. In-depth mechanistic investigations indicated that DUSP22 directly interacted with FAK and restrained its activation through dephosphorylating FAK at Y397 and Y576 + Y577 residues, subsequently restraining its downstream NF-κB and ERK1/2 signaling cascades. We further discovered that the PTP motif containing the functional site (C88S) of the DUSP22 protein was required for FAK blockage. Therefore, DUSP22 functioning independently with other DUSPs may be a promising therapeutic target for the treatments of NASH and its related HCC via restraining FAK signaling (Fig. 9).

The pathogenesis of NASH is complicated that includes a complex reprogrammed molecular network^3,5–7^. ROS is an essential pro-steatotic stimulus and a potential therapeutic target for NASH management, and a number of antioxidants have been used in clinical trials^47,48^. Unfortunately, the outcome for the clinical application of antioxidants is not upbeat with controversial issues^49^. Therefore, targeting more specific regulators for NASH is necessary for effective treatment. In the study, we, for the first time, discovered that ROS modulated DUSP22 degradation and substantially facilitated NASH development, while NAC treatment upregulated DUSP22 expression levels, protecting hepatocytes and liver tissues from damage under oxidative or metabolic stresses. The potent effects of ROS on the expression of DUSP22 revealed in our present study were similar to previous reports that oxidation of catalytic cysteine within the active site of DUSPs restrains DUSP phosphatase activities and triggers their proteasomal degradation^50^.

DUSP22 is widely expressed in various different types of tissues and cells, thus mediating diverse pathophysiological processes, such as cell motility, tumor progression, and inflammation^19–22,26,51^. DUSP22 mainly regulates MAPKs signaling pathway, in which it especially activates JNK signal but not ERK or p38 MAPK pathway in mammalian cells^23^. On the contrary, DUSP22 was also reported to dephosphorylate and inactivate JNK and p38, but not ERK, in the transfected COS-1 cells^52^. Furthermore, DUSP22 expression was discovered to restrain T-cell antigen receptor-triggered ERK2 activation in Jurkat T cells^53^. Therefore, the impacts of DUSP22 on MAPKs are controversial, which might be associated with the types of cells or tissues under different stimuli. Besides, DUSP22 could also depress IL-6-provoked STAT3 activation^24^. Aged mice with DUSP22 deficiency spontaneously exhibited elevated serum contents of pro-inflammatory cytokines (IL-6, TNF-α, IFN-γ, and IL-17A)^26,54^. Additionally, DUSP22-absent mice exerted aggravated inflammation and autoimmunity compared to wide-type mice and were more likely to develop experimental autoimmune encephalomyelitis (EAE). Mechanistically, DUSP22 can activate T-cell receptor (TCR) signaling through dephosphorylating and inactivating Lck at Y397, and DUSP22-knockout T cells presented abundant expression of inflammation cytokines^26^. Inflammation is considered a pivotal player in the pathogenesis of metabolic disorders. Inflammatory signals, such as ERK1/2, JNK, and NF-κB pathways, are essential complex links that connect inflammation with metabolic regulation, liver damage, and dysfunction, contributing to NASH progression^55^. Our findings demonstrated that DUSP22 expression both in vivo and in vitro was significantly decreased in response to a metabolic stimulus. Consistently, we also showed that DUSP22 exerted anti-inflammatory function upon HFHC or PO challenge, which was mainly through inactivating ERK1/2 and NF-κB signaling pathways but had no significant influence on JNK and p38 MAPK. Thus, we supposed that DUSP22 functions are cell type and/or stimuli dependent. The fatty liver creates a pro-inflammatory circumstance, which in turn causes HSCs activation, leading to excessive collagen accumulation in NASH or fatty liver-related HCC patients^56^. In addition to anti-inflammatory role, the effects of DUSP22 on lipid metabolism and fibrosis remain elusive. Here we newly verified the capability of hepatic DUSP22 to improve glucose tolerance, lipid metabolism, and collagen deposition, subsequently minimizing the detrimental effects on NASH development. Moreover, our results that the beneficial effects of DUSP22 overexpression on NASH were independent of weight gain, strongly illuminating that DUSP22 could directly impact hepatocytes without altering body weight.

DUSP22 was previously found to dephosphorylate FAK and suppress cell motility^21^. FAK consists of N-terminal FERM, Kinase, and C-terminal focal adhesion targeting (FAT) domains^57,58^, and modulates diverse cellular processes ranging from metabolic disorders to inflammatory response and cell survival, which depend on the particular stimulus or context^59,60^. FAK activation is carried out first through autophosphorylation at Y397 residue and subsequently through phosphorylation at Y576 + Y577^61^. FAK activation has been implicated in pro-inflammatory gene expression upon stimuli^62^. Moreover, FAK inhibitor remarkably abolished the NF-κB transcriptional activity and inflammatory cytokine gene expression in *Trypanosoma cruzi* (*Tc*)-infected macrophages^63^. MAPKs are known as down-streaming regulators of FAK signaling, and FAK-dependent MAPK activation participates in not only inflammation but also fibrosis^38,57^. Furthermore, the role of FAK in the regulation of glycogen synthesis in HepG2 cells and hepatic insulin signaling in vitro has also been reported^64^. Additionally, siRNA-mediated FAK knockdown promotes lipid oxidation, whereas damnifies glycogen synthesis in skeletal muscle^59^, suggesting that FAK suppression may elevate metabolic flexibility. FAK activation is also associated with cancer progression by enhancing cell survival, proliferation, and migration^65^. Recently, FAK phosphorylation was found to be upregulated in HCC tissues compared with normal liver tissues and was involved in NAFLD-related HCC progression partly via promoting HCC cell proliferation^66^. Thereafter, FAK activation is critically involved in metabolism disorders and tumor growth. Here in our study, we also showed that FAK was a major downstream target regulating the function of DUSP22 in NASH. We provided a better understanding of FAK activation in NASH induced by metabolic stimuli. Notably, a negative correlation between DUSP22 protein expression and FAK activation was detected in the liver of NASH patients. More importantly, we identified that the region of the DUSP22 protein from 1 to 144 aa domain containing C88S could bind to FAK, and directly interact with FAK via its FERM and Kinase domains, thereafter removing its activation through dephosphorylating FAK at Y397 and Y576 + Y577 residues. Finally, we surprisingly found that DUSP22 reduced tumor size and number in DEN/HFHC-induced mouse model with NASH-related HCC, which was validated by the decreased HCC cell proliferation in vitro, indicating that DUSP22 may act as a tumor suppressor to further restrain NASH development into HCC. Meanwhile, higher FAK activation was also detected in the liver of NASH-HCC patients. Notably, DUSP22^HepKO^-accelerated NASH-HCC progression was considerably abolished in DEN/HFHC-treated mice with FAK^HepKO^, indicating that the capacity of DUSP22 to restrain NASH-HCC was largely attributed to its inhibitory role on FAK activation and its downstream signals. Taken together, all these findings illustrated that DUSP22 could alleviate lipid accumulation, inflammation, hepatic fibrosis, and NASH-HCC progression, primarily relying on the FAK inactivation.

The DUSP family can dephosphorylate DUSPs’ substrates at threonine/serine residues and/or tyrosine residues. All members of the DUSP family contain a common phosphatase domain^50,54^. For example, DUSP3 deletion contributes to higher ERK and p38 phosphorylation, thereby promoting obesity and NASH in HFD-fed mice^27^. DUSP9 and DUSP12 retards ASK1 phosphorylation, thereby suppressing MAPKs phosphorylation and subsequent inflammation inhibition, consequently ameliorating hepatic steatosis progression^28,29^. Similarly, DUSP14 and DUSP26 target and restrain the phosphorylation of TAK1, an upstream regulator of MAPKs signaling, in hepatocytes, leading to MAPKs inactivation and inflammation inhibition, which contributes to NASH treatment^30,31^. DUSP22 has a canonical PTP signature motif, *HCXXGXXR*, at residues from 87 to 94^21,23^. Our in vitro study further illustrated that upon PO stimulation, DUSP22 phosphatase-dead mutant (DUSP22-C88S) lost its ability to dephosphorylate and inactivate FAK, and thereafter failed to reduce lipid deposition, inflammatory response via its downstream ERK1/2 and NF-κB cascades in hepatocytes, and fibrosis in human HSCs. Consistently, mutation of PTP motif containing C88S residue in DUSP22 also disabled to prohibit FAK phosphorylation at Y397 and Y576 + Y577, contributing to dyslipidemia, inflammatory response, and collagen accumulation. These results further elaborated that dephosphorylation activity of DUSP22 mediated by PTP motif was indispensable for DUSP22-regulated suppression of FAK signaling, thereby exerting protective effects against steatohepatitis. Similarly, catalytic domain mutations abrogated the dephosphorylating capacity of DUSP14 on TAK1, accelerating hepatic injury^31^. According to our current findings, previous studies, and the characteristic of DUSPs family, we demonstrated that the dephosphorylating capacity of DUSPs family members (DUSP22, DUSP3, DUSP9, DUSP12, DUSP14, DUSP26, etc) might be indispensable for its protective role against hepatic steatosis. In addition to these already reported DUSPs family members, DUSP22 is a newfound and worthy target for the development of promising therapeutic strategies in NASH-HCC. However, given that numerous cellular events are involved in HCC development^67^, there are still limitations in our present work. First, considering that the pathogenesis of NASH is a complex process involving metabolic disorder and uncontrolled chronic inflammation and fibrosis, it is interesting to investigate whether other DUSPs roads will exert a similar protective effect in the progression of individuals or mouse models with simple fatty liver, severe hepatic steatosis, and even NASH-HCC. Second, the downstream molecules of FAK signaling, such as cell cycle and apoptosis that may be involved in DUSP22-suppressed NASH-HCC have not been fully understood. Meanwhile, besides FAK, whether other targets participated in DUSP22-regulated NASH-HCC, continued research and discovery programs are desperately required and eagerly awaited.

Taken together, our present study provided further evidence that supported the protective effect of DUSPs family against hepatic steatosis. In brief, we, for the first time, found that ROS-mediated DUSP22 degradation participated in the setting and progression of fatty liver, contributing to the etiopathogenesis of NASH and associated HCC via promoting the phosphorylation of FAK at Y397 and Y576 + Y577 residues and thereafter aggravating its downstream ERK1/2 and NF-κB signaling cascade. Therefore, targeting DUSP22 for its upregulation and/or its interaction with FAK may be a useful therapy for the treatment of NASH-HCC.

## Methods

### Antibodies and reagents

The primary antibodies against anti-GAPDH (#2118), anti-p-JNK (#4668), anti-JNK (#9258), anti-p38 (#8690), anti-p-p38 (#4511), anti-p-IκBα (#2859), anti-p-TBK1 (#5483), anti-p-NF-κB (#3033) and anti-IκBα (#4814) were obtained from Cell Signaling Technology Inc (CST, Beverly, USA). Antibodies against anti-IKKα (#ab32041), anti-MEK1/2 (#ab178876), anti-p-MEK1/2 (#ab278564), anti-ERK1/2 (#ab184699), anti-p-ERK1/2 (#ab201015), anti-NF-κB (#ab16502), anti-TGF-β1 (#ab179695), anti-TAK1 (#ab50431), anti-TBK1 (#ab40676), anti-HA (#ab18181), anti-Flag (#ab205606), anti-CD11b (#ab133357), anti-DUSP22 (#ab70124), anti-DUSP3 (#ab248113), anti-EGFP (#ab184601), anti-HNF4a (#ab201460), anti-CK19 (#ab52625) and anti-α-SMA (#ab124964) were purchased from Abcam (Cambridge, MA, USA). Antibodies against anti-p-IKKα (#PA5-36652), anti-p-FAK^Y397^ (#44-624 G), anti-p-FAK^Y576+Y577^ (#PA5-37706), anti-FAK (#PA5-88093), anti-p-TAK1 (#PA5-99340), anti-HA (#PA1-985), anti-Flag (#MA1-91878), anti-F4/80 (#41-4801-82), anti-Ki-67 (#PA5-19462) anti-p-ASK1 (#PA5-105027), anti-ASK1 (#PA5-20200), anti-DUSP8 (#PA5-18007), anti-DUSP9 (#PA5-106527), anti-DUSP12 (#PA5-89113), anti-DUSP14 (#PA5-15565), anti-DUSP16 (#PA5-23140), anti-DUSP26 (#PA5-22013), anti-Albumin (#PA5-89332), and anti-PECAM (#PA5-32321) were purchased from Thermo Fisher Scientific, Inc., Waltham, USA). Antibodies against anti-DUSP22 (#H00056940-B01P and #NBP1-83078) were obtained from Novus Biologicals (USA). Diethylnitrosamine (DEN; Cat#55-18-5), palmitate (PA; Cat#P9767), oleic acid (OA; Cat#O1008), ROS scavenger N-acetyl-cysteine (NAC; Cat#A7250), and lipopolysaccharide (LPS; derived from *Escherichia coli* (055:B5); Cat#L2880) were purchased from Sigma–Aldrich (St. Louis, USA). Recombinant human IL-6 (Cat#206-IL-050/CF), TNF-α (Cat#210-TA-020/CF), and TGF-β1 (Cat#240-B-002) proteins were purchased from R&D system (Minneapolis, USA).

### Human samples

Human liver tissue samples were collected from adult patients with nonalcoholic fatty liver disease who underwent liver transplantation or liver biopsy under anesthesia. The corresponding control liver tissue was harvested from the donor who could not be used for liver transplantation because of non-hepatic reasons. Non-steatosis (n = 16), and NASH liver (*n* = 20) samples were collected and included in this study. Briefly, specimens with a NASH activity score (NAS) of 0 were classified as non-steatotic. Samples with a NAS ≥ 5 or a NAS of 3–4 but showing fibrosis were included in the NASH group. Steatotic liver samples from patients meeting any of the following criteria were excluded from the study: excessive alcohol consumption (>140 g for male or >70 g for female, per week), drug abuse, or viral infection (including infection with hepatitis B virus or hepatitis C virus). Human NAFLD-associated HCC tumor tissues and adjacent normal tissues were obtained from patients with biopsy-proven NAFLD-HCC (*n* = 8). Informed and written consent was obtained from all subjects or their family members before participating in this study. The characteristics, liver injury-associated serology, and NAS for NASH patients were listed in Supplementary Tables S1 and S2, and clinical information of NAFLD-HCC subjects was displayed in Supplementary Table S3. All procedures involving human subjects used in this study were conformed to the principles outlined in the Declaration of Helsinki, and completely approved by the Academic Research Ethics Committee in Chongqing Key Laboratory of Medicinal Resources in the Three Gorges Reservoir Region and other participating units.

### Animal experiments

All procedures and protocols for animal experiments were approved by the Animal Care and Use Committee of all participating Units. For this investigation, we focused on male mice based on the fact that in humans, NAFLD/NASH is more prevalent in males compared with females^68^. Mice were allowed to adapt to their living environment for one week before all experiments properly started. All animals were housed in a constant temperature, humidity (controlled by GREE central air-conditioner, #GMV-Pd250W/NaB-N1, China), and pathogen-free-controlled environment (23–25 °C, 50–60%) cage with a standard 12 h light/12 h dark cycle, plenty of water and food (pathogen-free) in their cages.

### Animal strains

All the male, normal wild-type (WT) C57BL/6 N mice (6–8 week old; 22–25 g body weight) used in the current study were purchased from Beijing Vital River Laboratory Animal Technology Co., Ltd. (Beijing, China). The age-matched (6–8 week old) male ob/ob mice (#N000103) were purchased from Nanjing Biomedical Research Institute of Nanjing University).

To generate mice with a conditional knockout allele of DUSP22, the DUSP22^flox/flox^ mice with C57BL/6 N background were constructed using CRISPR/Cas9-regulated genome engineering system. Exon 3 of DUSP22 was selected as a conditional knockout region (cKO). Briefly, the selected exon of DUSP22 was flanked by two loxP sites, and therefore two single guide RNAs (gRNA1# and gRNA2#) targeting DUSP22 introns were designed. The targeting vector containing DUSP22 exon 3 flanked by two loxP sites and the two homology arms was used as the template. The targeting vector, gRNA1#, and gRNA2#, together with Cas9 were co-injected into fertilized eggs for cKO mouse production. The obtained mice, which had exon 3 flanked by two loxP sites on one allele, were used to establish DUSP22^flox/flox^ mice. Hepatocyte-specific DUSP22 deletion (DUSP22^HepKO^) mice were produced by mating DUSP22^flox/flox^ mice with albumin-Cre (Alb-Cre) mice (Jackson Laboratory, Bar Harbor, Maine, USA). A simple schematic diagram has been indicated in Supplementary Fig. S6A. DUSP22^flox/flox^ (Flox) mice littermates were used in work as controls for the obtained DUSP22^HepKO^ mice.

To obtain mice with a conditional knock in of DUSP22, the RosaDUSP22 mice with C57BL/6 N background were constructed using DUSP22 conditional knock in at the locus of Rosa26 in mice by CRISPR/Cas-regulated genome engineering system. In brief, the Rosa26-pCAG-loxp-STOP-loxp-mDUSP22-pA cassette was cloned into intron 1 of Rosa26. Also, to engineer the targeting vector, homology arms were then constructed by PCR using BAC clone as a template. Thereafter, the targeting vector, gRNA, and Cas9 were co-injected into fertilized eggs for RosaDUSP22 mouse production. In indicated experiments, the conditional overexpression of DUSP22 in hepatocyte (DUSP22^HepOE^) was induced through injection of adeno-associated virus-serotype 8 (AAV8)-thyroxine-binding globulin (TBG) promoter-Cre recombinase vector (AAV8-TBG-Cre) via intravenous injection and then determined using immunoblotting analysis. A simple schematic diagram has been indicated in Supplementary Fig. S10A. RosaDUSP22 mice littermates without AAV injection were used as controls for the obtained DUSP22^HepOE^ mice.

The hepatocyte-specific FAK-knockout (FAK^HepKO^) mice with a deletion in the exon 4 of FAK were produced using protocols similar to the one described for the establishment of the DUSP22^flox/flox^ mice and DUSP22^HepKO^ mice. Briefly, FAK^flox/flox^ mice were designed and constructed by CRISPR/Cas-mediated genome engineering system. The exon 4 of FAK gene was selected as a conditional knockout region. To engineer the targeting vector, homology arms and cKO region were generated by PCR using BAC clone from the C57BL/6 N library as a template. Then Cas9 and gRNA were co-injected into fertilized eggs with a targeting vector for mice production. Then, the FAK^flox/flox^ mice were crossed with Alb-Cre mice to produce hepatocyte-specific FAK deficiency mice (FAK^HepKO^). A simple schematic diagram has been indicated in Supplementary Fig. S21A. FAK-floxed mice littermates were used as controls for the obtained FAK^HepKO^ mice.

The hepatocyte-specific DUSP22 (DUSP22^HepKO^) and FAK (FAK^HepKO^) double deletion (Hep-DKO) mice were generated by crossing DUSP22^flox/flox^ mice with FAK^HepKO^ mice. The obtained offspring without DUSP22 and FAK protein expression were identified, confirmed, and selected using western blotting analysis, and were used for further in vivo experiments. A simple schematic diagram has been indicated in Supplementary Fig. S21D.

### Establishment of NASH mouse model

Two different NASH models in mice were constructed and used in the study for the corresponding experiments. First, a high-fat plus high-cholesterol diet (HFHC)-induced NASH mouse model was established by feeding the male mice with a HFHC fodder (containing 42% saturated fat, 14% protein, 44% carbohydrates, and 0.2% cholesterol w/w) for 24 weeks^69^. The mice fed with a normal chow diet (20% protein, 10% fat, and 70% carbohydrate, #D12450H; Research Diets, New Brunswick, NJ, USA) for 24 weeks were defined as controls (NCD). The second NASH model was performed using a high-fat diet plus methionine- and choline-deficient diet (HFMCD). Briefly, the male mice were fed with a HFMCD diet (HFMCD, A06071301B, Research Diets) for 8 weeks^17,70^. Control mice received a corresponding normal chow diet (NCD, Specialty Feeds).

### Animal study design 1#

The 6–8-week-old WT male mice were fed with a HFHC diet for 24 weeks to induce NASH phenotype, and WT mice fed with a standard NCD for 24 weeks were defined as the control group (NCD). The age-matched (6–8 week old) ob/ob mice were served as another fatty liver model and fed with a NCD. At the end of the experimental periods, the liver samples were collected from all mice for further analysis.

### Animal study design 2#

To examine the role of ROS in DUSP22 expression during NASH progression, 6–8-week-old WT male mice were fed with a HFHC diet for 24 weeks. NAC (150 mg/kg) was subjected to mice via intraperitoneal injection twice a week to eliminate ROS in HFHC-challenged mice^17^. Vehicle groups of mice received the same volume of saline.

### Animal study design 3#

Hepatocyte-specific DUSP22 deletion (DUSP22^HepKO^) male mice were fed with a HFHC diet for 24 weeks to induce NASH phenotype. To obtain the conditional DUSP22 gain-of-function (GOF) mice, HFHC diet-fed RosaDUSP22 mice were injected with a 1.5 × 10^12^ genome copies (gc) dose of an AAV8-TBG-Cre vectors through the tail vein to induce hepatocyte-specific DUSP22 overexpression (DUSP22^HepOE^/HFHC). RosaDUSP22 mice given an equal dose of AAV empty vector (EV) were served as a control (DUSP22^HepRosa^/HFHC). A simple schematic diagram has been indicated in Fig. 3a.

### Animal study design 4#

To explore the function of DUSP22 on HFHC diet-induced NASH pathologies, the ex vivo-mediated DUSP22 gene therapy interventions through lentivirus-packaged full-length DUSP22 sequences (LV-DUSP22) or shRNA targeting DUSP22 (LV-shDUSP22) transduction and transplantation were performed in 8-week HFHC-fed preconditioned WT male mice. The detailed protocols of the ex vivo therapeutic experiments were established according to our previous report^71^. The brief diagram of the experimental design was presented in Fig. 7a. At the end of the ex vivo therapy interventions, the mice fasted for 8 h, and then the eye blood and liver samples were harvested, weighed, and stored at −80 °C for further animal studies. The other parts of liver tissues were subjected to histological analysis and biochemical analysis.

### Animal study design 5#

To obtain another NASH diet-induced steatohepatitis, DUSP22^HepKO^ and DUSP22^HepOE^ male mice were fed a HFMCD diet for 8 weeks to create NASH phenotype. DUSP22^HepKO^ and DUSP22^HepOE^ mice were generated as above described. DUSP22^flox^ and DUSP22^Rosa^ mice were used as control, respectively. All mice were subjected to a 8-week HFMCD or NCD. A simple schematic diagram has been indicated in Supplementary Fig. S14A and B.

### Animal study design 6#

To further explore the effects of DUSP22 in NASH progression, full-length mouse DUSP22 sequences or mouse DUSP22 sequences with PTP-motif domain deletion were loaded in AAV8 vector to create AAV-TBG-DUSP22 or AAV-TBG-DUSP22 (ΔPTP motif) vectors. The DUSP22^HepKO^ mice were subsequently injected with a 1.5 × 10^12^ genome copies (gc) dose of corresponding vectors to produce DUSP22 gain-of-function (GOF) male mice. These mice were then fed with a 24-week HFHC diet to trigger NASH phenotype (Hep^DUSP22^GOF/HFHC, Hep^DUSP22-ΔPTP motif^ GOF/HFHC). The corresponding mice injected with blank vectors were served as controls. A simple schematic diagram has been indicated in Supplementary Fig. S20A.

### Animal study design 7#

The male DUSP22^HepKO^ mice, FAK^HepKO^ mice, Hep-DKO mice, and Flox mice were fed with a HFHC diet for 24 weeks to generate NASH phenotype and explore the pathological alterations.

### Animal study design 8#

To further explore the protective effects of DUSP22 against NASH progression. The male FAK^HepKO^ mice and FAK^flox^ mice were injected with/without LV-shDUSP22 vector through a portal vein to establish hepatocyte-specific DUSP22-FAK loss-of-function mice or hepatocyte-specific DUSP22 loss-of-function mice before a 24-week of HFHC diet feeding (FAK^HepKO^/HFHC) or halfway by HFHC feeding (FAK^HepKO^/LV-shDUSP22/HFHC, FAK^flox^/LV-shDUSP22/HFHC). A simple schematic diagram has been indicated in Supplementary Fig. S22A.

### Animal study design 9#

To investigate the function of DUSP22 expression and FAK activation on NASH-associated HCC, a NASH-HCC animal model was then established. The male WT C57BL/6 N mice, DUSP22^flox^, DUSP22^HepKO^, DUSP22^HepRosa^, DUSP22^HepOE^, FAK^HepKO^, and hepatocyte-specific double knockout of DUSP22 and FAK (Hep-DKO) mice that were created as mentioned above were i.p. injected with a single dose of DEN (25 mg/kg) at the age of 2 weeks and fed a HFHC diet beginning at the age of 8 weeks. NASH-HCC establishment in ob/ob mice was performed by i.p. injection with a single dose of DEN (25 mg/kg) at the age of 2 weeks and was then subjected to NCD diet. After 24 weeks of HFHC diet feeding, the mice were sacrificed, and their serum and liver tissue samples were collected. The number of tumors on the liver surface and the tumor size was measured and analyzed.

During the experiments, body weight, fasting blood glucose, fasting serum insulin concentration, and food intake were recorded and measured at different time points as indicated in each figure legend.

### Cell culture

All resuscitated cell lines used in our laboratory were passaged no more than 30 times. Cell lines involved in experiments need to be tested for mycoplasma contamination by PCR analysis. Human normal hepatocyte cell line L02 was purchased from the Type Culture Collection of the Chinese Academy of Sciences (Shanghai, China), and was cultured in Dulbecco’s Modified Eagle Medium (DMEM) (#22320030, Gibco®) supplemented with 10% fetal bovine serum (FBS; #10100147, Gibco®) and 1% penicillin-streptomycin (#SV30010; Hyclone) in a humidified incubator (Thermo Fisher Scientific) with 5% CO_2_ at 37 °C. Human hepatic stellate cell (HSC) line LX2 and human HCC cell lines (Hep3B and HepG2) were obtained from Merck Millipore (Shanghai, China) and American Type Culture Collection (ATCC; Manassas, VA, USA), respectively. Human HCC cell line SMMC-7721 was obtained from the Shanghai Cell Bank Type Culture Collection Committee (CBTCCC, Shanghai, China). All cells were maintained in DMEM medium with 10% FBS and 1% penicillin-streptomycin in a 5% CO_2_ incubator at 37 °C. Mouse primary hepatocytes used in the study were isolated from corresponding indicated groups of mice using the liver perfusion method as described previously^69,71^. Briefly, mice’s abdominal cavity was opened under a painless anesthesia condition. Thereafter, the liver tissue was carefully perfused with 1× liver perfusion medium (#17701-038, Gibco™) and 1× liver digest medium (#17703-034, Gibco™) via the portal vein. Subsequently, 100 µm steel mesh was used to grind and filter the digested liver samples. The mice’s primary hepatocytes were then collected by centrifuging the filter liquor at 800 rpm, 4 °C for 5 min, and were further purified using 50% percoll solution (#17-0891-01, GE Healthcare Life Sciences). The obtained hepatocytes were maintained in DMEM medium containing 10% FBS and 1% penicillin-streptomycin and cultured at 37 °C in a cell incubator with 5% CO_2_. To imitate the in vivo liver lipid deposition and steatosis, cells were incubated with the indicated dose of PO (0.4 mM PA and 0.8 mM OA), TNF-α, IL-6, LPS, or TGF-β1 as demonstrated in figure legends to investigate the function of DUSP22 in vitro experiments.

### Construction of the knockout cell lines

The generation and protocol of FAK-deficient L02 cell line were performed as described previously^72^. Briefly, L02 cell lines with FAK ablation was produced by CRISPR/Cas9 gene editing system. The small guide RNA (sgRNA) targeting the human FAK gene was produced and cloned into lentiCRISPRV2 vectors to generate the Cas9-sgRNA lentivirus. The oligo sequences used for the generation of sgRNA expression vector are shown as: sgFAK-1#: 5’-ACTGGTATGGAACGTTCTCC-3’; sgFAK-2#: 5’-TGAGTCTTAGTACTCGAATT-3’. Subsequently, the packaging vectors pMD2.G and psPAX2, together with the corresponding sgRNA expression vector, were then transfected separately into HEK293T cells using Lipofectamine™ CRISPRMAX™ Cas9 or FuGENE® 6 Transfection Reagent for 42 h. Then, the L02 cells were transduced with the obtained supernatant containing lentivirus to construct the targeted gene deficiency cell lines. The cell clones with target gene knockout were selected using immunoblotting analysis.

### Vectors establishment and transfection

To over-express DUSP22, human full-length DUSP22 expression vectors were generated by PCR-based amplification of cDNA, and then cloned into the 3×Flag-tagged pcDNA3.1 vector or 3× HA-tagged pcDNA3.1 vector (Invitrogen), respectively. Truncated human DUSP22 and FAK fragments expression plasmid including FAK-HA, DUSP22-Flag, DUSP22 (1–144)-Flag, DUSP22 (145–184)-Flag, and FAK (1–400)-HA, FAK (1–693)-HA, FAK (401–693)-HA, FAK (401–904)-HA, FAK (694–904)-HA, FAK (694–1052)-HA, and FAK (905–1052)-HA as indicated in the Fig. 5f and Fig. 5g and figure legends, were produced using standard PCR methods and were then cloned into corresponding vectors. The Flag-FAK WT expression plasmid was constructed based on pcDNA3.1 vector. Moreover, FAK and corresponding derivatives, including FAK in which the only complete amino acid residue was FAK-FERM, FAK-Kinase, and FAK-FRNK, were then packed into the Flag-tagged pcDNA3.1 vector (Thermo Fisher Scientific). The obtained vectors were carefully transfected into L02 or FAK-knockout L02 cells using Lipofectamine™ 3000 Transfection Reagent (Invitrogen™) according to the manufacturer’s protocols. Furthermore, to explore the effects of DUSP22 in vitro experiments, we constructed an adenovirus-packaged targeted gene expression vector. Human or mouse full-length DUSP22 sequences and specific short hairpin RNA oligonucleotides sequences targeting human or mouse DUSP22 (shDUSP22) (shRNA sequences RNAi#1: 5’-TACCTGTGCATCCCAGCAG-3’; #2: 5’-ACACTGGTGATCGCATACA-3’ for human; RNAi#1: CAGGAGTTTGAGAAACATGAA; RNAi#2: 5’-CCCATGTTGGAGGGAGTTAAA-3’ for mouse), human WT DUSP22 sequences with C88S mutation [DUSP22 (C88S), the Cys → Ser substitution]^21^, and targeting human or mouse MISSION® shRNA Plasmid DNA FAK (shFAK) obtained from Sigma–Aldrich (SHCLND-NM_153831, SHCLND-NM_007982) were respectively packaged into adenovirus (Ad-DUSP22, Ad-DUSP22 (C88S), Ad-shDUSP22, and Ad-shFAK) by Easy Adenoviral Vector System Kit (#240009, Agilent Technologies). The Ad-GFP and Ad-shRNA (scrambled RNA) were served as controls, respectively. The recombinant adenovirus was purified and titrated to 5 × 10^10^ plaque-forming units (PFU). The verification of the virus is based on DNA analysis of the virus, which is a plaque virus purified by restriction enzymes. To generate the LV-DUSP22 or LV-shDUSP22 vectors, the mouse DUSP22 cDNA sequences or shRNA targeting mouse DUSP22 sequences were packaged into pLenti-RFP-Puro-CMV or pLenti-U6-EGFP to down-regulate or up-regulate DUSP22 expression for in vivo experiments.

### Liver function indicators and lipid contents determination

The concentration of serum alanine transaminase (ALT) (#MAK052, Sigma–Aldrich), aspartate aminotransferase (AST) (#MAK055, Sigma–Aldrich), alkaline phosphatase (AKP) (#ab83369, Abcam), serum insulin (#ab277390, Abcam), and hepatic triglyceride (TG) (#MAK266, Sigma–Aldrich), total cholesterol (TC) (#ab65359, Abcam) and nonesterified free fatty acids (NEFA) (#E-BC-K014, Elabscience, Inc., Houston, USA) were detected using commercially available detection kits according to the manufacturer s’ protocols.

### Co-Immunoprecipitation (Co-IP) analysis

The immunoprecipitation detection was conducted in the study as previously described^69,71^. Briefly, the cells or liver tissues were homogenized into IP-specific lysis solution (#87787, Pierce™ IP Lysis Buffer, Thermo Scientific Pierce) at 4 °C, followed by centrifugation at 13,000 rpm for 20 min. The cell lysates were then collected and incubated with Protein A/G Magnetic Agarose Beads (#78609, Thermo Scientific Pierce) at room temperature with mixing for 1–2 h, and were subsequently co-incubated with the indicated antibodies at 4 °C overnight. After rinsing with Kit-containing immunoprecipitation wash buffer, the immune complex was collected and subjected to immunoblotting analysis with the shown primary antibodies and the corresponding secondary antibodies.

### Glutathione S-transferase (GST) pull-down assay

Direct protein interaction between DUSP22 and FAK was conducted using the GST pull-down assay as previously reported^14^. The Quick Pierce™ GST Protein Interaction Pull-Down Kit (#21516, Thermo Fisher Scientific) was used to examine the protein binding. In brief, the Rosetta (DE3) *Escherichia coli* (*E. coli*) cells were transformed with the plasmid pGEX-4T-1-GST-DUSP22 or pGEX-4T-1-GST-FAK and then induced expression through incubation with 0.5 mM isopropyl β-D-thiogalactopyranoside (IPTG) (#I5502, Sigma–Aldrich). The extractions from lytic *E. coli* were mixed with corresponding GST beads at 4 °C for 1 h. Then, the GST beads were co-incubated with Flag-tagged DUSP22 or Flag-tagged FAK, which were prepared by IP for another 4 h. Proteins that had interacted were eluted in elution buffer and were then subjected to immunoblotting using anti-Flag antibodies. The *E. coli* expressing only a GST-tag was served as the negative control.

### RNA extraction, quality control, and high-throughput quantitative PCR (HTqPCR)

Total RNA in liver samples or cells was extracted using TRIzol™ reagent (#15596-018, Thermo Fisher Scientific) according to the manufacturer’s instructions. Then, 1 µg of total RNA extraction was reverse transcribed using the M-MLV-RT system (Invitrogen), which was performed at 42 °C for 1 h and terminated by deactivation of the enzyme at 70 °C for 10 min. Subsequently, PCR was carried out with SYBR Green (Bio-Rad) on an ABI PRISM 7900HT system (Applied Biosystems, USA). The specific primer sequences were produced by Invitrogen or Generay Biotech (Shanghai, China), and listed in Supplementary Table S4. Fold induction values were calculated according the 2^(−ΔΔCt)^ expression. ΔCt represents the differences in cycle threshold number between the target gene and GAPDH, and ΔΔCt represents the relative change in the differences between the control and treatment groups.

For the high-throughput quantitative PCR (HTqPCR), the TaqMan® Low Density Array (P/N 4342259, TLDA cards, ABI, Hilden, Germany) with 384-well microfluidic cards was used for comparative analysis of gene expression according to the manufacturer’s instructions. The TLDA cards used in the present study are customized by ABI company following the requirements of our present experiment. The obtained cards are based on TaqMan chemistry in which gene expression of a panel of 128 genes was analyzed in 1 run. Also, the TLDA cards were pre-loaded with targeted TaqMan gene expression assays of importance for 87 inflammation-related genes, 25 lipid metabolism-related genes, and 16 pro-fibrosis-related genes, and 6 controls were designed for normalization. The obtained gene expression alterations were normalized to the mean of 5 out of 6 controls (GAPDH, β-actin, β-tubulin, B2M, HPRT1) of the same sample (ΔCT), chosen in accordance with their stability. Data from any one randomly selected group were defined as an etalon (ΔΔCT), and data from all other groups were normalized to it. Gene expression levels were finally indicated as relative expression (presenting as fold change (2^-ΔΔCT^) method for calculation). TaqMan gene expression Master Mix (P/N 4369510, Applied Biosystems) was used when running the HTqPCR. The ABI Prism SDS 2.1 software attached to ABI PRISM 7900HT system was used for data analysis and quantification. The obtained final data and results were presented as a heatmap image.

### Western blotting

Cells or liver samples were homogenized into RIPA Lysis and Extraction Buffer (#89900, Thermo Fisher Scientific) to yield a homogenate. Then, the final liquid supernatants were centrifuged at 13,500 rpm, 4 °C for 30 min. Protein concentration was examined using PierceTM Rapid Gold BCA Protein Assay Kit with BSA as a standard following the supplier’s protocols. Total protein extraction samples were then subjected to western blotting analysis. Equal amounts of the obtained total protein (20-50 μg) were subjected to 10 or 12% sodium dodecyl sulfate polyacrylamide gel electrophoresis (SDS-PAGE) system and then transferred to a 0.45 µM PVDF membrane (#10600023, Amersham Hybond, GE Healthcare Life Science, Germany). Subsequently, the PVDF membranes were blocked with 5% skim milk (DifcoTM Skim Milk, BD, USA) in 1× TBS buffer (#T1080, Solarbio, Beijing, China) containing 0.1% Tween-20 (#abs9152, Absin, China) (TBST) for 1 h and mixed with the primary antibodies (diluted at 1:1000 or 1:500) at 4 °C overnight. Thereafter, the PVDF membranes were washed with 1× TBST for three times and subsequently co-treated with horseradish peroxidase (HRP)-conjugated anti-rabbit (#ab6721) or anti-mouse (#ab6789) secondary antibodies (Abcam, dilution 1:8000) for 1.5 h at room temperature. Western blotting bands were visualized using a PierceTM ECL Plus Western Blotting Substrate (#32134, Thermo Fisher Scientific) and exposed to Kodak (Eastman Kodak Company, USA) Xray film. Corresponding protein expression levels were subsequently determined as gray values (Version 1.52 v, Image J, National Institutes of Health, USA) and standardized to the housekeeping gene (GAPDH) and expressed as a fold of control.

### Metabolic indicators and serum cytokines parameters assessment

GTT was performed on mice that had been fasted for 8 h. Mice were intraperitoneally injected with glucose (2 g/kg body weight) (#158968, Sigma–Aldrich). Then, the concentration of blood glucose in tail venous blood at 0, 15, 30, 60, and 120 min after glucose injection was measured with the commercial blood glucose test strips (ACCU-CHEK®, Roche Diabetes Care GmbH, Shanghai, China). Finally, the fasting insulin and fasting glucose levels were used to calculate the homeostatic model assessment of insulin resistance (HOMA-IR). The following equation was used to calculate HOMA-IR values: Blood (Glucose (mg/dL) × (Serum Insulin (μU/mL)/405^73^. For mice, the cytokines or chemokines contents in serum were examined using corresponding commercial enzyme-linked immuno sorbent assay (ELISA) kits, including the mouse TNF-α (#MTA00B), IL-1β (#MLB00C), IL-6 (#M6000B), IL-10 (#M1000B) and MCP-1 (#MJE00B) ELISA kits purchased from R&D system according to the manufacturer’s protocols. The corresponding serum was carefully stored at −80 °C the fridge until used. For human, the serum contents of TNF-α (#DTA00D), IL-18 (#DY318-05), IL-6 (#D6050), MCP-1 (#DCP00) and TGF-β1 (#DY240) were measured using corresponding human ELISA kits (R&D system) according to the manufacturer’s instructions.

### Intracellular TG and inflammatory cytokines assay

Intracellular TG contents were measured using commercial Triglyceride Assay Kit Quantification (#ab65336, Abcam) according to the manufacturer’s protocols. For mouse primary hepatocytes, the cytokines in the medium were calculated using corresponding commercial ELISA kits, including the TNF-α (#MTA00B), IL-1β (#MLB00C) and IL-6 (#M6000B) that were all obtained from R&D system following the manufacturer’s instructions. For L02 cells, the contents of TNF-α (#DTA00D), IL-1β (#QLB00B), and IL-6 (#D6050) in the collected medium were assessed using corresponding ELISA kits (R&D system) in accordance with the procedures recommended by the manufacturer.

### Histopathologic and immunohistochemistry staining

To explore histopathologic and immunohistochemical changes, the liver tissues were consequently fixed with 4% formaldehyde-histological tissue fixative (#R37814, Image-iT™, Invitrogen™), embedded in paraffin (#YA0010, Solarbio Life Sciences, Beijing, China), and then sectioned transversely (5 µm thick). The thin liver tissue sections were stained with hematoxylin and eosin (H&E) (#ab245880, Hematoxylin and Eosin Staining Kit, Abcam) to visualize the pattern of lipid accumulation and inflammation of livers. NAS score following H&E analysis is the sum of the scores of three components, including steatosis (0–3), lobular inflammation (0–3), and hepatocyte ballooning (0-2). To further indicate lipid accumulation in livers, the sections were frozen in Tissue-Tek optimum cutting temperature (O.C.T.) (#4583, Tissue-Tek, Sakura Finetek, USA) and then stained with Oil Red O Stain Kit (#ab150678, Abcam) for 10 min. After being washed with 60% isopropyl alcohol (#I9030, Sigma–Aldrich), the liver sections were re-stained with hematoxylin. Additionally, to examine collagen accumulation in liver tissues, sections were stained with Masson trichrome stain (#G1346, Masson’s Trichrome Stain Kit, Solarbio Life Sciences) and Sirius red stain (#ab150681, Picro Sirius Red Stain Kit, Abcam). To perform the immunohistochemistry assay, embedded sections were dewaxed, and antigens were retrieved via sodium citrate heating. Endogenous peroxidase was removed by adding 30% H_2_O_2_, and an immunohistochemical pen was used to draw a circle around the tissue. Then, 5% goat serum (#C0265, Beyotime, Shanghai, China) was added to block the liver tissues. The sections were then incubated with primary antibody anti-CD11b (#ab133357, Abcam, dilution 1:200) and DUSP22 (#NBP1-83078, Novus Biologicals, USA; dilution 1:200) at 4 °C overnight in the indicated groups. Sections were then washed three times with PBS for 3 min, followed by incubation with anti-rabbit IgG (HRP) secondary antibody (#ab6721, Abcam, dilution 1:200) for 1 h at room temperature. Immunohistochemical staining was observed using 3,3’-diaminobenzidine (DAB) substrate kit (#ab64238, Abcam) and was counterstained with hematoxylin. All images were captured using a light microscope (Olympus, Japan) for tissue observation. The positive-staining area was analyzed and quantified with Image-Pro Plus software (Version 6.0), and the results were expressed as a percentage (%) of the total area of a high-power filed (HPF).

### Immunofluorescence staining

For immunofluorescence microscopy, the frozen liver sections (5 µm thick) were placed for 20 min at room temperature and then washed with PBS for three times. Liver sections were blocked in 10% goat serum (#C0265, Beyotime) containing 0.3% Triton X-100 (#ST797, Beyotime) for 1 h at room temperature and incubated with primary antibodies against DUSP22 (#H00056940-B01P, dilution 1:200), phospho-FAK (Tyr576 + 577) (#PA5-37706, dilution 1:100), phospho-FAK (Tyr397) (#44-624 G, dilution 1:100), F4/80 (#41-4801-82, dilution 1:200), Ki-67 (#PA5-19462, dilution 1:200), EGFP (#ab184601, dilution 1:50), Albumin (#PA5-89332, dilution 1:100), HNF4a (#ab201460, dilution 1:100), CK19 (#ab52625, dilution 1:50), PECAM (#PA5-32321, dilution 1:100), and α-SMA (#ab124964, dilution 1:50) at 4 °C overnight. Sections were then washed three times with PBS. Anti-rabbit IgG H&L (Alexa Fluor® 594) (#ab150080), anti-mouse IgG H&L (Alexa Fluor® 488) (#ab150113) or anti-rat IgG H&L (Alexa Fluor® 488) (#ab150165) secondary fluorescent antibodies (Abcam, dilution 1:300) were prepared for liver slides incubation at room temperature in the dark for 1 h. After washing with PBS, 2-(4-Amidinophenyl)−6-indolecarbamidine dihydrochloride solution (DAPI; #C1006, Beyotime) was added to the section for nuclei staining for 5 min followed by PBS washes. Images were visualized and captured under fluorescence microscopy (Olympus, Japan). For colocalization examination, L02 cells and mouse hepatocytes were co-transfected with plasmids encoding Flag-tagged DUSP22 (green) and HA-tagged FAK (red) for 24 h. Subsequently, the cells were double-stained with anti-Flag and anti-HA antibodies, followed by the corresponding fluorophore-conjugated secondary antibodies (Abcam). Images were captured under fluorescence microscopy (Olympus, Japan).

### Measurement of oxidative stress-related parameters

Hepatic tissues were washed with cold PBS solution and then homogenized on ice. After centrifugation at 4 °C and 1000 × *g* for 15 min, the supernatants were harvested. The SOD activities were assessed to evaluate the antioxidases using a commercial kit (#A001-3-2), and the results are presented as the units of SOD per milligram (mg) of protein. The lipid peroxidation state of the liver was measured by testing the MDA level (#A003-1-2), which is presented as nmol/mg protein. The procedures were conducted according to the kit instructions provided by the manufacturer (Nanjing Jiancheng Bioengineering Institute, Nanjing, China). Liver tissues were lysed using a lysis buffer and were then centrifuged at 12000 × *g* for 5 min at 4 °C. The liquid supernatant was collected for the calculation of liver H_2_O_2_ levels as the commercial kit (#S0038, Beyotime) recommended provided by the manufacturer.

### Reactive oxygen species (ROS) in liver

The ROS contents in the liver were measured using dihydroethidium (DHE) staining (10 μM; #S0063, Beyotime) on fresh frozen liver sections (5 μm thick) at 37 °C for 30 min in a humidified and dark chamber according to the manufacturer’s instructions. Images were captured using a fluorescence microscope (Olympus, Japan), and the percentage of the DHE-stained area was analyzed and quantified with Image J software (Version 1.52 v, National Institutes of Health, USA).

### Cell viability

Cell viability was tested using a Cell Counting Kit-8 assay kit according to the manufacturer’s instructions. Briefly, stably transfected HepG2 and SMMC-7721 cells were seeded in 96-well plates (5000 cells/well). After incubation for the indicated times, 10 μL of Cell Counting Kit-8 reagent (#C0039, Beyotime) was added to each well. After incubation for 4 h at 37 °C. The absorbance of each well at 450 nm was measured by a microplate reader (SpectraMax iD3, Molecular Devices, USA) to examine the number of viable cells.

### Nile Red staining

At 24 h post-transfection, the cells were washed twice with PBS and fixed with 4% paraformaldehyde for 10 min at room temperature. After another PBS rinse, the cells were stained for 15 min with Nile Red (#7385-67-3, Solarbio) at room temperature. After washing with PBS, nuclei staining was performed using DAPI (#C1006, Beyotime) for an additional 5 min. Fluorescent imaging was captured by using fluorescence microscopy (Olympus, Japan).

### EdU staining assay

The stably transfected HepG2 and SMMC-7721 cells (5 × 10^4^) were seeded in a 24-well plate. The assay was performed using the BeyoClick™ EdU Cell Proliferation Kit with Alexa Fluor 594 imaging Kit (#C0078S, Beyotime). EdU staining was performed following the manufacturer’s protocols. Then, DAPI (#C1006, Beyotime) solution was incubated with cells for 5 min at room temperature. Images were visualized and captured under fluorescence microscopy (Olympus, Japan). The EdU-positive cell rate was based on the ratio of EdU-positive nuclei (red)/fluorescent nuclei (blue).

### Terminal deoxynucleotidyl transferase dUTP nick-end labeling (TUNEL) staining in vitro

One Step TUNEL Apoptosis Assay Kit (#C1090, Beyotime) was used to examine the apoptosis in the stably transfected HepG2 and SMMC-7721 cells according to the manufacturer’s protocols. After washing with PBS twice and fixing with 4% paraformaldehyde for 30 min, the cells were permeabilized in 0.3% Triton X-100 (#ST797, Beyotime) for 5 min at room temperature. Then, the cells were washed with PBS twice and incubated with TUNEL detection solution at 37 °C for 1 h in the dark, followed by nuclei staining with DAPI (#C1006, Beyotime) for an additional 5 min. After rinsing with PBS three times, the red fluorescence of apoptotic cells was captured using a fluorescent microscope (Olympus, Japan). The apoptosis was calculated as TUNEL-positive cells (red)/DAPI (blue).

### Statistical analysis

Quantitative values of data were expressed as mean ± standard error of the mean (SEM). Student’s two-tailed *t*-test was performed to compare the means of two-group samples, and one-way analysis of variance (ANOVA) was used for comparison of multiple groups, followed by Bonferroni’s post hoc test (for data showing homogeneity of variance) or Tamhane’s T2 (M) post hoc test (for data showing heteroscedasticity). GraphPad Prism Software (Version 9.2.0; Graph Pad Software, Inc., San Diego, CA) or SPSS Statistics Software (Version 26.0.0.2; IBM, Inc., New York, USA) was used for the final data analysis. *P*-value <0.05 was considered as significant. Randomization and blinding manner were used whenever possible.

### Reporting summary

Further information on research design is available in the Nature Research Reporting Summary linked to this article.

## Supplementary information

Supplementary Information

Reporting Summary

## Data Availability

The data that support the findings of this work are available from the corresponding author on request. There are no restrictions on data availability in the current work. Source data are provided with this paper.

## Source data

Source Data
