# Supplementary material for: Hepatocyte phosphatase DUSP22 mitigates NASH-HCC progression by targeting FAK
Source: Nat Commun. 2022 Oct 8;13:5945. doi: 10.1038/s41467-022-33493-5 (PMC9547917; doi:10.1038/s41467-022-33493-5)

SUPPLEMENTARY INFORMATION for:

Hepatocyte phosphatase DUSP22 mitigates NASH-HCC progression by targeting FAK

Ge et al.

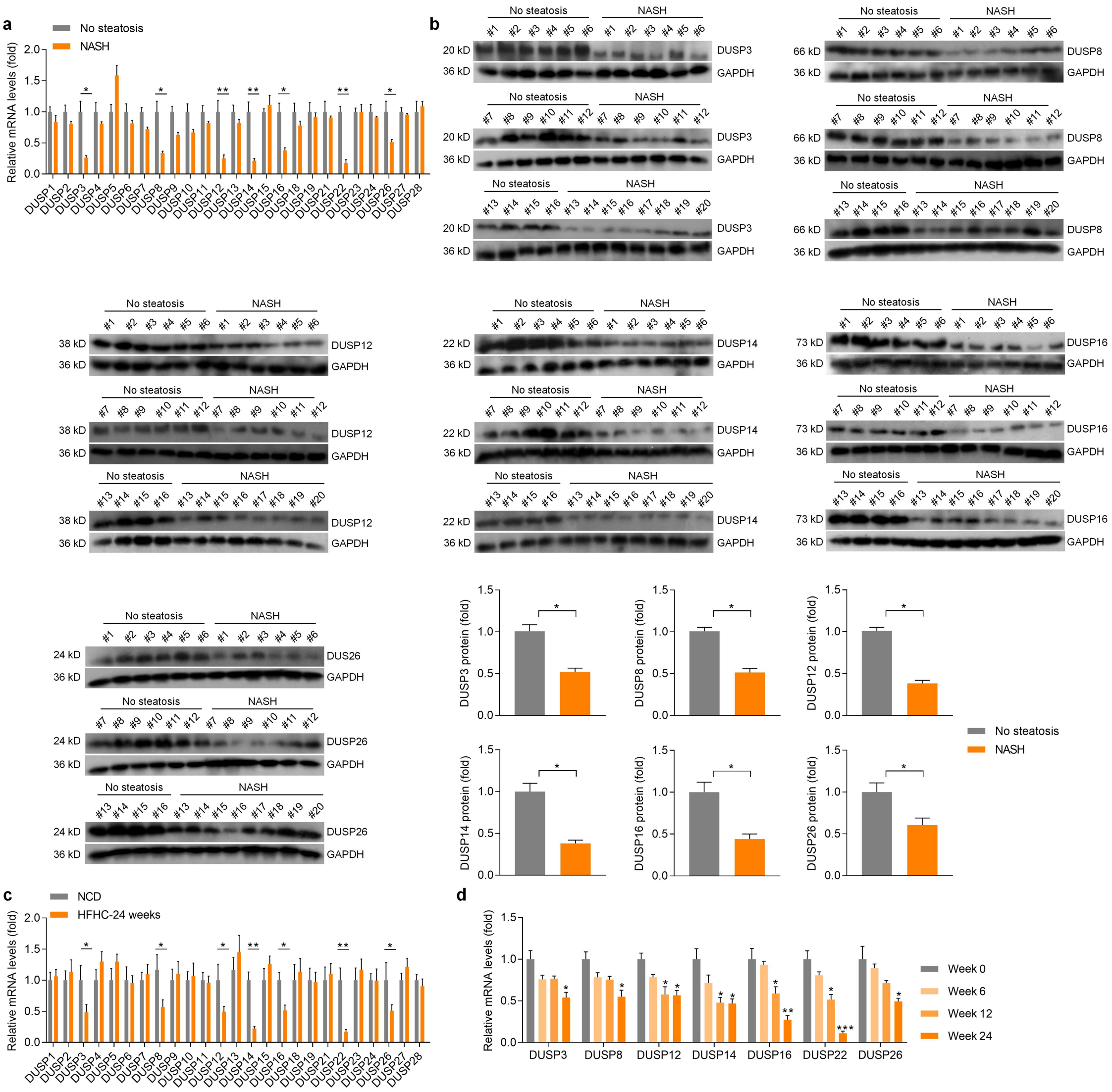
a
No steatosis
NASH
Relative mRNA levels (fold)
DUSP1 DUSP2 DUSP3 DUSP4 DUSP5 DUSP6 DUSP7 DUSP8 DUSP9 DUSP10 DUSP11 DUSP12 DUSP13 DUSP14 DUSP15 DUSP16 DUSP18 DUSP19 DUSP21 DUSP22 DUSP23 DUSP24 DUSP26 DUSP27 DUSP28
b
No steatosis
NASH
20 kD
36 kD
DUSP3
GAPDH
66 kD
DUSP8
38 kD
DUSP12
22 kD
DUSP14
73 kD
DUSP16
24 kD
DUS26
DUSP26
DUSP3 protein (fold)
DUSP8 protein (fold)
DUSP12 protein (fold)
DUSP14 protein (fold)
DUSP16 protein (fold)
DUSP26 protein (fold)
c
NCD
HFHC-24 weeks
Relative mRNA levels (fold)
d
Relative mRNA levels (fold)
Week 0
Week 6
Week 12
Week 24
DUSP3 DUSP8 DUSP12 DUSP14 DUSP16 DUSP22 DUSP26

Supplementary Figure 1. DUSPs expression changes in clinical NASH patients and HFHC-induced mouse models. a RT-qPCR analysis for the mRNA expression levels of DUSPs family members in livers from individuals without (No steatosis; n=16) or with NASH (n=20) ($^{*}P$<0.05 and $^{**}P$<0.01). **b** Western blotting results for DUSP3, DUSP8, DUSP12, DUSP14, DUSP16 and DUSP26 protein expression liver in livers from individuals without (No steatosis; n=16) or with NASH (n=20) ($^{*}P$<0.05). **c** RT-qPCR analysis for the mRNA expression levels of DUSPs family members in livers isolated from mice fed with NCD or HFHC for 24 weeks (n=6 per group) ($^{*}P$<0.05 and $^{**}P$<0.01). **d** RT-qPCR analysis for hepatic DUSP3, DUSP8, DUSP12, DUSP14, DUSP16, UDSP22 and DUSP26 gene expression levels of HFHC-treated mice at the shown time points (n=8 per group) ($^{*}P$<0.05, $^{**}P$<0.01 and $^{***}P$<0.001). Data are expressed as mean ± SEM from at least three independent experiments. For statistical analysis, **a-c** were conducted by two-tailed Student's *t*-test; **d** was performed by one-way ANOVA.

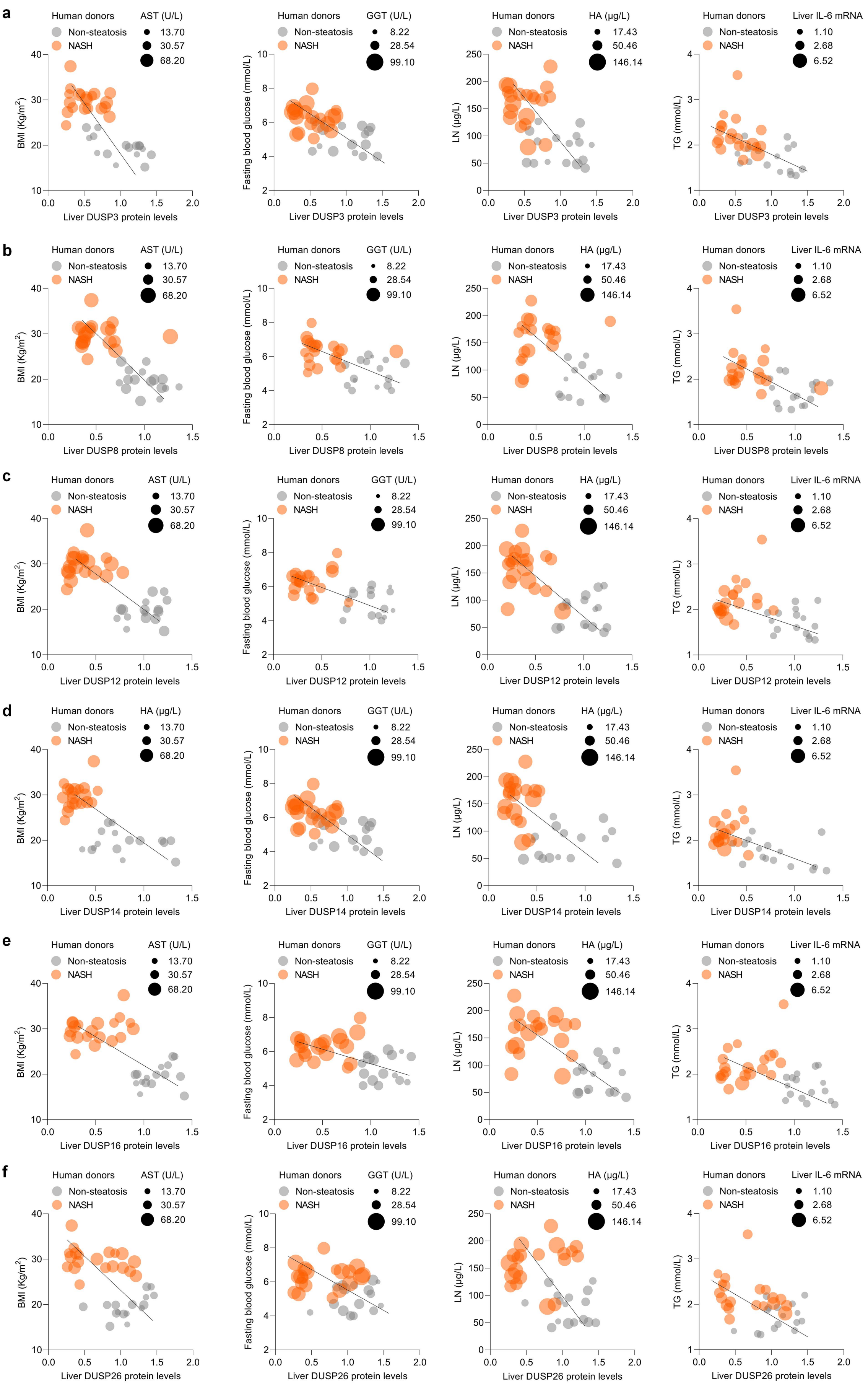

a
Human donors
Non-steatosis
NASH
AST (U/L)
13.70
30.57
68.20
BMI (Kg/m2)
Liver DUSP3 protein levels
GGT (U/L)
8.22
28.54
99.10
Fasting blood glucose (mmol/L)
HA (μg/L)
17.43
50.46
146.14
LN (μg/L)
Liver IL-6 mRNA
1.10
2.68
6.52
TG (mmol/L)
b
Liver DUSP8 protein levels
c
Liver DUSP12 protein levels
d
HA (μg/L)
13.70
30.57
68.20
Liver DUSP14 protein levels
e
Liver DUSP16 protein levels
f
Liver DUSP26 protein levels

Supplementary Figure 2. Correlation analysis between the expression of several DUSPs family members with clinical NASH severity. a-f Pearson correlation analysis showing the correlations between human liver (**a**) DUSP3, (**b**) DUSP8, (**c**) DUSP12, (**d**) DUSP14, (**e**) DUSP16 and (**f**) DUSP26 protein expression levels and body mass index (BMI), fasting blood glucose levels, serum laminin (LN) and serum TG levels. n=36 subjects per parameter. Correlations were performed using Spearman's rank correlation coefficient analysis.

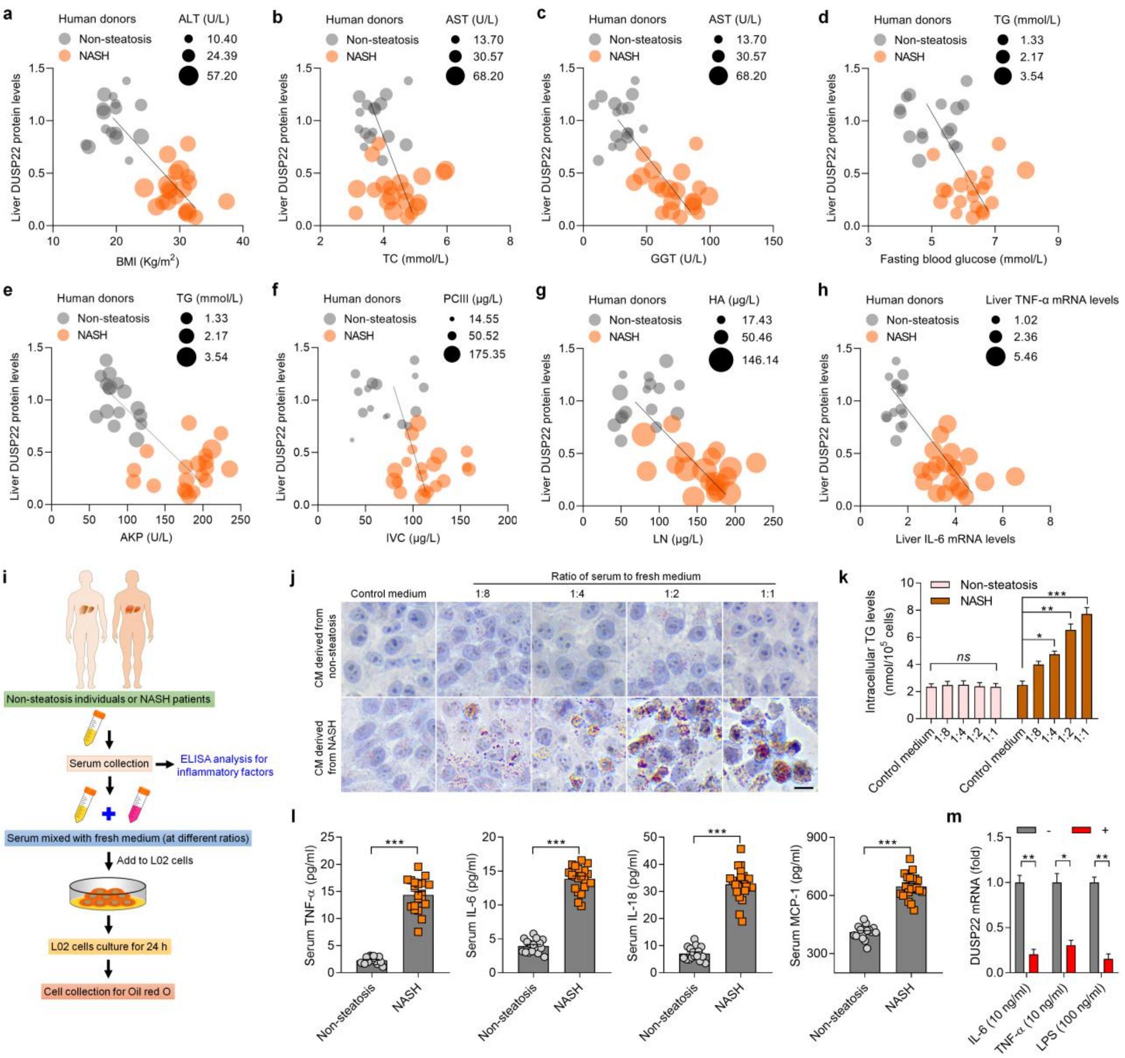

Human donors
Non-steatosis
NASH
Liver DUSP22 protein levels
BMI (Kg/m2)
TC (mmol/L)
GGT (U/L)
Fasting blood glucose (mmol/L)
AKP (U/L)
IVC (μg/L)
LN (μg/L)
Liver IL-6 mRNA levels
Non-steatosis individuals or NASH patients
Serum collection
ELISA analysis for inflammatory factors
Serum mixed with fresh medium (at different ratios)
Add to L02 cells
L02 cells culture for 24 h
Cell collection for Oil red O
Ratio of serum to fresh medium
Control medium
CM derived from non-steatosis
CM derived from NASH
Intracellular TG levels (nmol/10^5 cells)
Serum TNF-α (pg/ml)
Serum IL-6 (pg/ml)
Serum IL-18 (pg/ml)
Serum MCP-1 (pg/ml)
DUSP22 mRNA (fold)
IL-6 (10 ng/ml)
TNF-α (10 ng/ml)
LPS (100 ng/ml)

Supplementary Figure 3. DUSP22 is negatively correlated with NASH severity in human patients. a-h Pearson correlation analysis showing the correlations between human liver DUSP22 protein expression levels and (**a**) BMI, (**b**) serum TC levels, (**c**) serum γ-glutamyl transpeptidase (GGT) contents, (**d**) fasting blood glucose concentrations, (**e**) serum alkline phosphatase (AKP) activities, (**f**) collagen Type IV (IVC), (**g**) serum LN, and (**h**) liver IL-6 mRNA expression levels. n=36 subjects per parameter. **i** Scheme showing the L02 cell culture in serum collected from NASH patients or Non-steatosis individuals. **j** Representative Oil red O staining images and **k** intracellular TG contents in L02 cells after 24 h culture in the conditional medium mixed with different ratios of serum from NASH or Non-steatosis individuals and fresh medium as shown. Fresh medium only was served as control (n=5 per group, with 10 images per group; Scale bar, 25 μm) ($^{*}P$<0.05, $^{**}P$<0.01 and $^{***}P$<0.001; *ns*, no significant difference). **l** Examination of inflammatory factors including TNF-α, IL-6, IL-18 and MCP-1 in serum collected from NASH or Non-steatosis individuals (n=36 subjects in total) ($^{***}P$<0.001). **m** RT-qPCR results for DUSP22 mRNA expression levels in L02 cells exposed to IL-6 (10 ng/ml), TNF-α (10 ng/ml) or LPS (100 ng/ml) for 24 h (n=4 per group) ($^{*}P$<0.05 and $^{**}P$<0.01). Data are expressed as mean ± SEM from at least three independent experiments. For statistical analysis, correlations in **a-h** were performed using Spearman's rank correlation coefficient analysis; **k-m** were conducted by two-tailed Student's *t*-test.

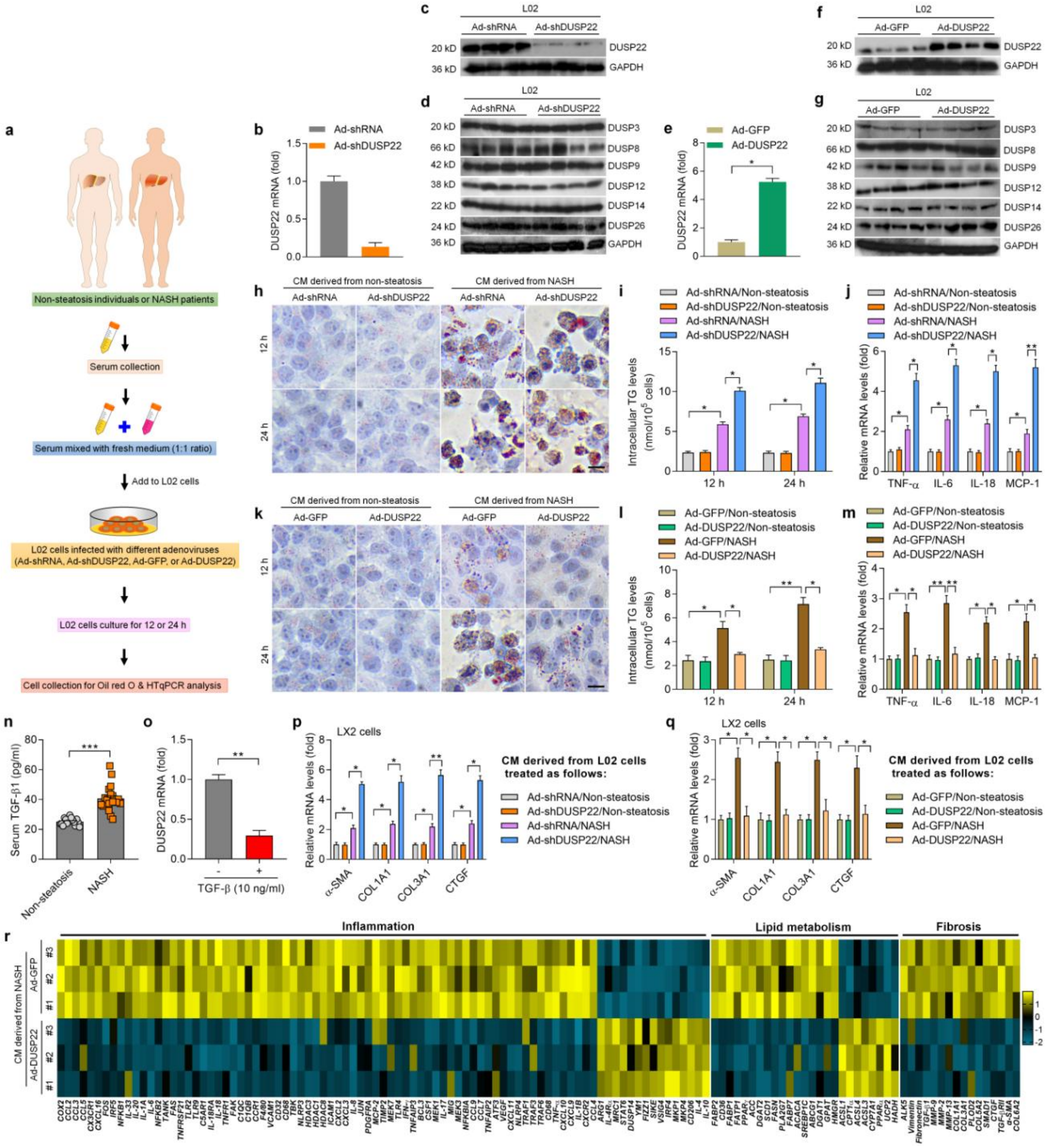

a
Non-steatosis individuals or NASH patients
Serum collection
Serum mixed with fresh medium (1:1 ratio)
Add to L02 cells
L02 cells infected with different adenoviruses (Ad-shRNA, Ad-shDUSP22, Ad-GFP, or Ad-DUSP22)
L02 cells culture for 12 or 24 h
Cell collection for Oil red O & HTqPCR analysis
b
Ad-shRNA
Ad-shDUSP22
DUSP22 mRNA (fold)
c
L02
Ad-shRNA
Ad-shDUSP22
20 kD
36 kD
DUSP22
GAPDH
d
L02
Ad-shRNA
Ad-shDUSP22
DUSP3
DUSP8
DUSP9
DUSP12
DUSP14
DUSP26
GAPDH
e
Ad-GFP
Ad-DUSP22
DUSP22 mRNA (fold)
f
L02
Ad-GFP
Ad-DUSP22
DUSP22
GAPDH
g
L02
Ad-GFP
Ad-DUSP22
h
CM derived from non-steatosis
CM derived from NASH
12 h
24 h
i
Ad-shRNA/Non-steatosis
Ad-shDUSP22/Non-steatosis
Ad-shRNA/NASH
Ad-shDUSP22/NASH
Intracellular TG levels (nmol/10^5 cells)
j
Relative mRNA levels (fold)
TNF-α
IL-6
IL-18
MCP-1
k
CM derived from non-steatosis
CM derived from NASH
Ad-GFP
Ad-DUSP22
l
Ad-GFP/Non-steatosis
Ad-DUSP22/Non-steatosis
Ad-GFP/NASH
Ad-DUSP22/NASH
m
n
Serum TGF-β1 (pg/ml)
Non-steatosis
NASH
o
DUSP22 mRNA (fold)
TGF-β (10 ng/ml)
p
LX2 cells
Relative mRNA levels (fold)
α-SMA
COL1A1
COL3A1
CTGF
CM derived from L02 cells treated as follows:
Ad-shRNA/Non-steatosis
Ad-shDUSP22/Non-steatosis
Ad-shRNA/NASH
Ad-shDUSP22/NASH
q
LX2 cells
CM derived from L02 cells treated as follows:
Ad-GFP/Non-steatosis
Ad-DUSP22/Non-steatosis
Ad-GFP/NASH
Ad-DUSP22/NASH
r
Inflammation
Lipid metabolism
Fibrosis
CM derived from NASH
Ad-GFP
Ad-DUSP22

Supplementary Figure 4. Hepatocyte DUSP22 expression mitigates lipid deposition, inflammation and fibrosis *in vitro*. a Scheme showing the L02 cell culture in serum collected from NASH patients or Non-steatosis individuals after transfection with adenovirus-loaded short hairpin RNA targeting DUSP22 (Ad-shDUSP22) or adenovirus-loaded full-length DUSP22 sequences (Ad-DUSP22) for 24 h. Ad-shRNA and Ad-GFP were used as the corresponding controls. **b-d** (**b**) RT-qPCR and (**c**) western blotting analysis for mRNA and/or protein expression levels of DUSP22, (**d**) DUSP3, DUSP8, DUSP9, DUSP12, DUSP14 and DUSP26 in L02 cells transfected with Ad-shDUSP22 or Ad-shRNA for 24 h (n=4 per group). **e-g** (**e**) RT-qPCR and (**f**) western blotting analysis for mRNA and/or protein expression levels DUSP22, (**g**) DUSP3, DUSP8, DUSP9, DUSP12, DUSP14 and DUSP26 in L02 cells transfected with Ad-DUSP22 or Ad-GFP for 24 h (n=4 per group). **h,i** (**h**) Representative Oil red O staining images, and (**i**) intracellular TG contents in L02 cells that were transfected with Ad-shDUSP22 or Ad-shRNA after culture in the conditional medium (CM) mixed with NASH or Non-steatosis serum and fresh medium (1:1 ratio) for 12 or 24 h (n=5 per group, with 10 images per group; Scale bar, 25 μm) ($^{*}P<0.05$). **j** RT-qPCR results for inflammatory factors TNF-α, IL-6, IL-18 and MCP-1 in L02 cells transfected with Ad-shDUSP22 or Ad-shRNA after culture in the CM mixed with NASH or Non-steatosis serum and fresh medium (1:1 ratio) for 24 h (n=4 per group) ($^{*}P<0.05$ and $^{**}P<0.01$). **k,l** (**k**) Representative Oil red O staining images, and (**l**) intracellular TG contents in L02 cells that were transfected with Ad-DUSP22 or Ad-GFP post culture in the CM mixed with NASH or Non-steatosis serum and fresh medium (1:1 ratio) for 12 or 24 h (n=5 per group, with 10 images per group; Scale bar, 25 μm) ($^{*}P<0.05$ and $^{**}P<0.01$). **m** RT-qPCR results for inflammatory factors TNF-α, IL-6, IL-18 and MCP-1 in L02 cells transfected with Ad-DUSP22 or Ad-GFP after culture in the CM mixed with NASH or Non-steatosis serum and fresh medium (1:1 ratio) for 24 h (n=4 per group) ($^{*}P<0.05$ and $^{**}P<0.01$). **n** Assessment of TGF-β1 in serum collected from NASH or Non-steatosis individuals (n=36 subjects in total) ($^{***}P<0.001$). **o** RT-qPCR results for DUSP22 mRNA expression levels in L02 cells exposed to TGF-β1 (10 ng/ml) for 24 h (n=4 per group) ($^{**}P<0.01$). **p,q** CM was collected from L02 cells that were transfected with (**p**) Ad-shDUSP22, Ad-shRNA, (**q**) Ad-DUSP22 or Ad-GFP as shown after culture in the medium mixed with NASH or Non-steatosis serum and fresh medium (1:1 ratio) for 24 h (n=4 per group), and was then used for LX2 cell culture for another 24 h through mixing with fresh medium (1:1 ratio) (n=4 per group) ($^{*}P<0.05$ and $^{**}P<0.01$). **r** HTqPCR analysis showing the alteration of inflammation (left) and lipid metabolism (middle) related genes expression in L02 cells treated as explained in (**m**), and the expression changes of fibrosis-related genes (right) in LX2 cells treated as described in (**q**) (n=3 per group). Data are expressed as mean ± SEM from at least three

independent experiments. Statistical analysis were performed by two-tailed Student's *t*-test.

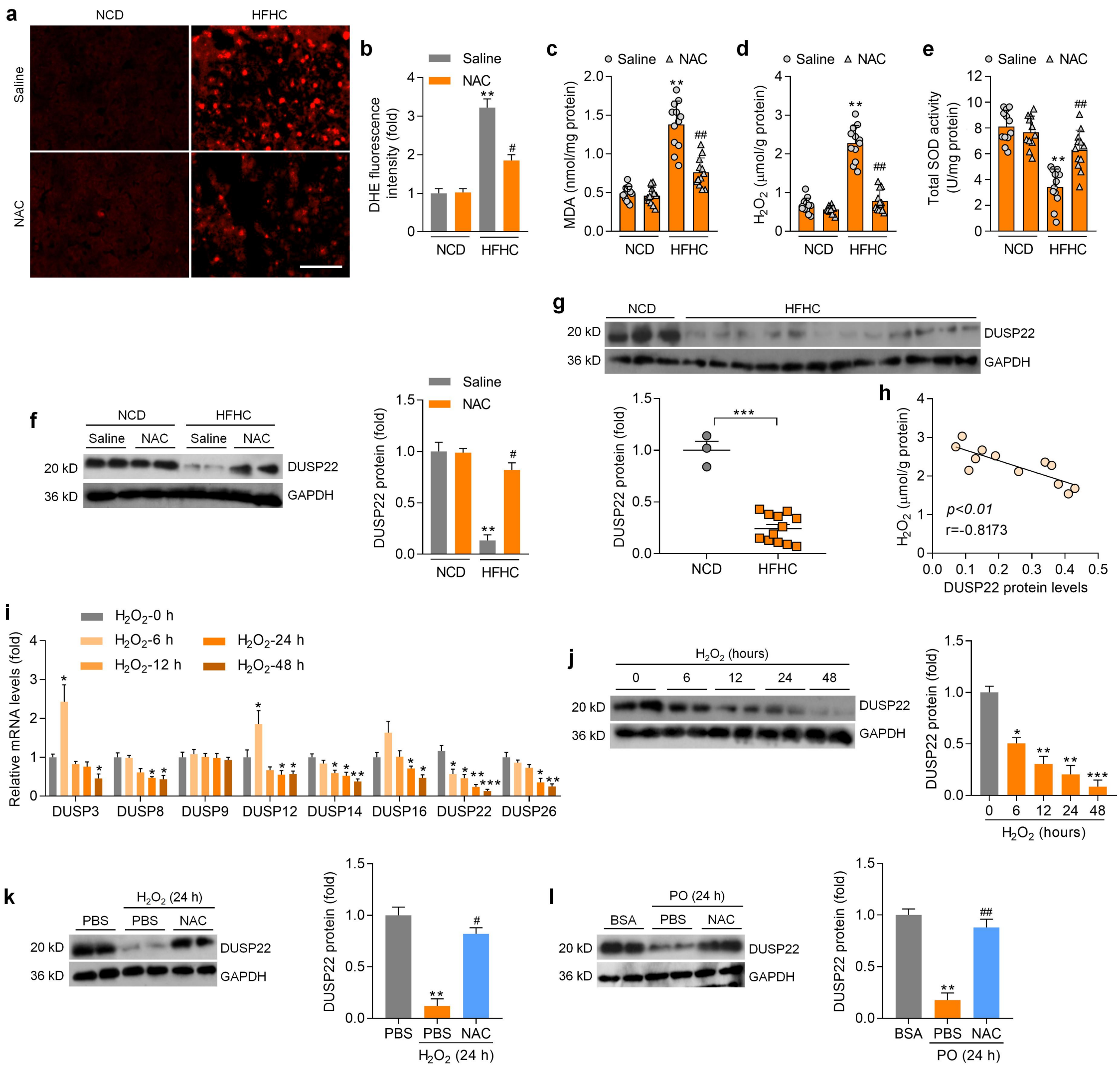

a
NCD
HFHC
Saline
NAC
b
Saline
NAC
DHE fluorescence intensity (fold)
NCD
HFHC
c
Saline
NAC
MDA (nmol/mg protein)
d
H_2O_2 (μmol/g protein)
e
Total SOD activity (U/mg protein)
f
20 kD
36 kD
DUSP22
GAPDH
DUSP22 protein (fold)
g
h
H_2O_2 (μmol/g protein)
DUSP22 protein levels
p<0.01
r=-0.8173
i
H_2O_2-0 h
H_2O_2-6 h
H_2O_2-12 h
H_2O_2-24 h
H_2O_2-48 h
Relative mRNA levels (fold)
DUSP3
DUSP8
DUSP9
DUSP12
DUSP14
DUSP16
DUSP22
DUSP26
j
H_2O_2 (hours)
k
H_2O_2 (24 h)
PBS
NAC
l
PO (24 h)
BSA

Supplementary Figure 5. ROS production negatively mediates DUSP22 expression during HFHC. a Representative image of DHE staining on the fresh frozen liver sections of WT mice after a 24-week HFHC feeding with or without NAC (N-acetyl-cysteine) administration (n=6 mice/group; Scale bar, 50 μm). **b** Quantification for DHE fluorescent intensity in the liver samples of mice from the shown groups of mice (n=6 mice per group) ($^{**}P$<0.01 versus the NCD/Saline group; $^{\#}P$<0.05 versus the HFHC/Saline group). **c-e** Examination for (**c**) MDA levels, (**d**) H_2O_2 contents, and (**e**) total SOD activity in liver tissues from the indicated groups of mice (n=11 or 12 mice per group) ($^{**}P$<0.01 versus the NCD/Saline group; $^{\#\#}P$<0.01 versus the HFHC/Saline group). **f** Representative western blotting analysis and quantification of DUSP22 protein expression in livers of the shown groups of mice (n=4 mice per group) ($^{**}P$<0.01 versus the NCD/Saline group; $^{\#}P$<0.05 versus the HFHC/Saline group). **g** Representative western blotting analysis for DUSP22 in liver samples from NCD-fed mice (n=3) or 24-week HFHC-treated mice (n=12) ($^{***}P$<0.001). **h** Pearson correlation analysis showing the correlation between hepatic DUSP22 protein expression levels and H_2O_2 contents from HFHC group of mice. **i** The mouse primary hepatocytes were incubated with H_2O_2 (100 μM) for 6, 12, 24 or 48 h, and were then collected for RT-qPCR analysis of DUSP3, DUSP8, DUSP9, DUSP12, DUSP14, DUSP16, UDSP22 and DUSP26 gene expression levels (n=6 per group) ($^{*}P$<0.05, $^{**}P$<0.01 and $^{***}P$<0.001 versus the H_2O_2-0 h group). **j** Protein expression of DUSP22 by western blotting analysis in mouse primary hepatocytes treated with H_2O_2 (100 μM) for 6, 12, 24 or 48 h (n=4 per group) ($^{*}P$<0.05, $^{**}P$<0.01 and $^{***}P$<0.001 versus the H_2O_2-0 h group). **k,l** Western blotting analysis for protein expression of DUSP22 in mouse primary hepatocytes treated with (**k**) H_2O_2 (100 μM) or (**l**) PO (0.4 mM PA and 0.8 mM OA) for 24 h in the presence or absence of NAC (5 mM). PBS was used as control (n=4 per group) ($^{**}P$<0.01 versus the PBS or BSA group; $^{\#}P$<0.05 and $^{\#\#}P$<0.01 versus the H_2O_2/PBS or PO/PBS group). Data are expressed as mean ± SEM from at least three independent experiments. For statistical analysis, **b-g, k** and **l** were conducted by two-tailed Student's *t*-test; **i** and **j** were performed by one-way ANOVA.

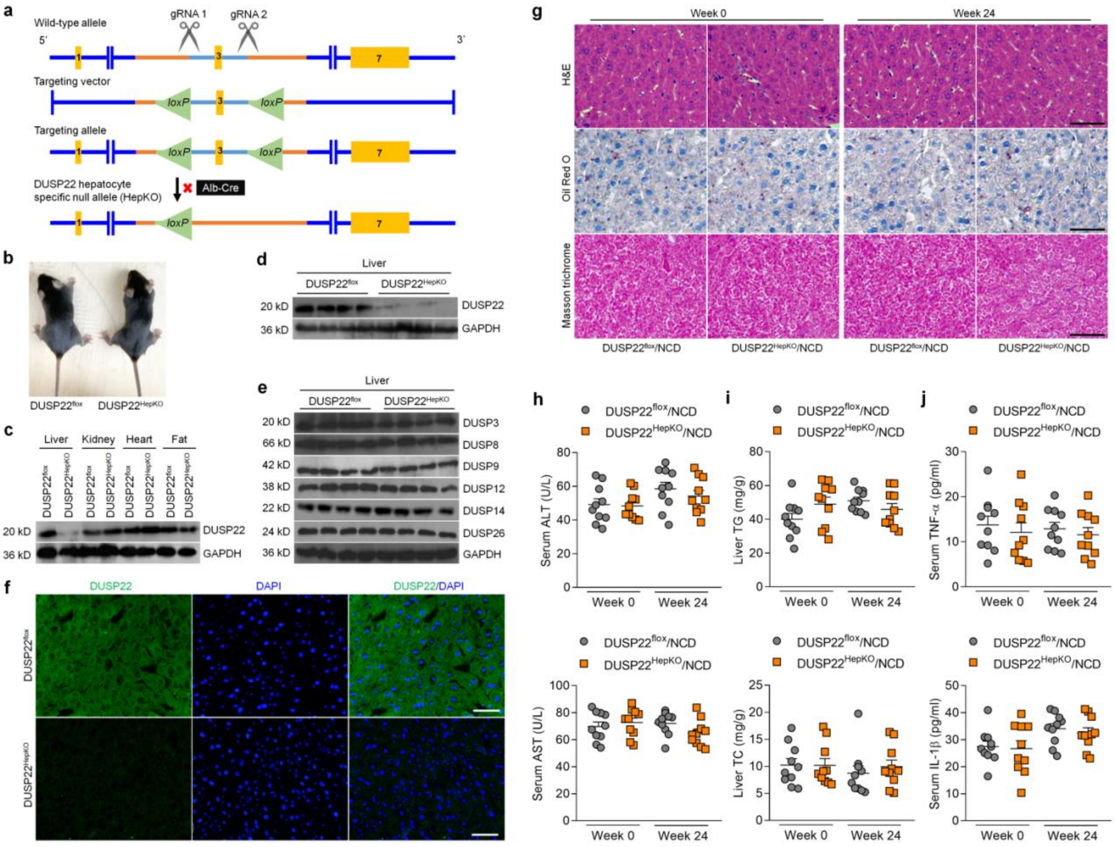

a
Wild-type allele
gRNA 1
gRNA 2
5'
3'
Targeting vector
loxP
Targeting allele
DUSP22 hepatocyte specific null allele (HepKO)
Alb-Cre
b
DUSP22flox
DUSP22HepKO
c
Liver
Kidney
Heart
Fat
20 kD
36 kD
DUSP22
GAPDH
d
Liver
DUSP22flox
DUSP22HepKO
e
DUSP3
DUSP8
DUSP9
DUSP12
DUSP14
DUSP26
f
DUSP22
DAPI
DUSP22/DAPI
g
Week 0
Week 24
H&E
Oil Red O
Masson trichrome
DUSP22flox/NCD
DUSP22HepKO/NCD
h
Serum ALT (U/L)
Serum AST (U/L)
i
Liver TG (mg/g)
Liver TC (mg/g)
j
Serum TNF-α (pg/ml)
Serum IL-1β (pg/ml)

Supplementary Figure 6. Construction of hepatocyte-specific DUSP22 knockout mice. **a** Schematic workflow showing the establishment of the hepatocyte-specific DUSP22-knockout (DUSP22^{HepKO}) mouse strain. **b** Images of 8-week-old male DUSP22^{flox} and DUSP22^{HepKO} mice. **c** Western blot showing DUSP22 expression in liver, kidney, heart, and fat of the indicated mice (n=3 mice per group). **d,e** Western blot showing (**d**) DUSP22, (**e**) DUSP3, DUSP8, DUSP9, DUSP12, DUSP14 and DUSP26 expression in liver from DUSP22^{flox} and DUSP22^{HepKO} mice (n=4 mice per group). **f** Representative immunofluorescence images of DUSP22 (green) in the livers of mice from the DUSP22^{flox} and DUSP22^{HepKO} mice, and nuclei were stained with DAPI (blue) (n=3 mice per group, with 10 images for each mouse; Scale bars, 40 μm). **g** H&E staining of liver sections from DUSP22^{flox} and DUSP22^{HepKO} mice at 0 and 24 weeks of NCD feeding (n=8 mice per group, with 10 images for each mouse; Scale bars, 50 μm). **h-j** Quantification for (**h**) serum ALT and AST contents, (**i**) liver TG and TC levels, and (**j**) serum TNF-α and IL-1β concentrations in DUSP22^{flox} and DUSP22^{HepKO} mice at 0 and 24 weeks of NCD feeding (n=10 per group). Data are expressed as mean ± SEM from at least three independent experiments. For statistical analysis, **h-j** were performed by one-way ANOVA.

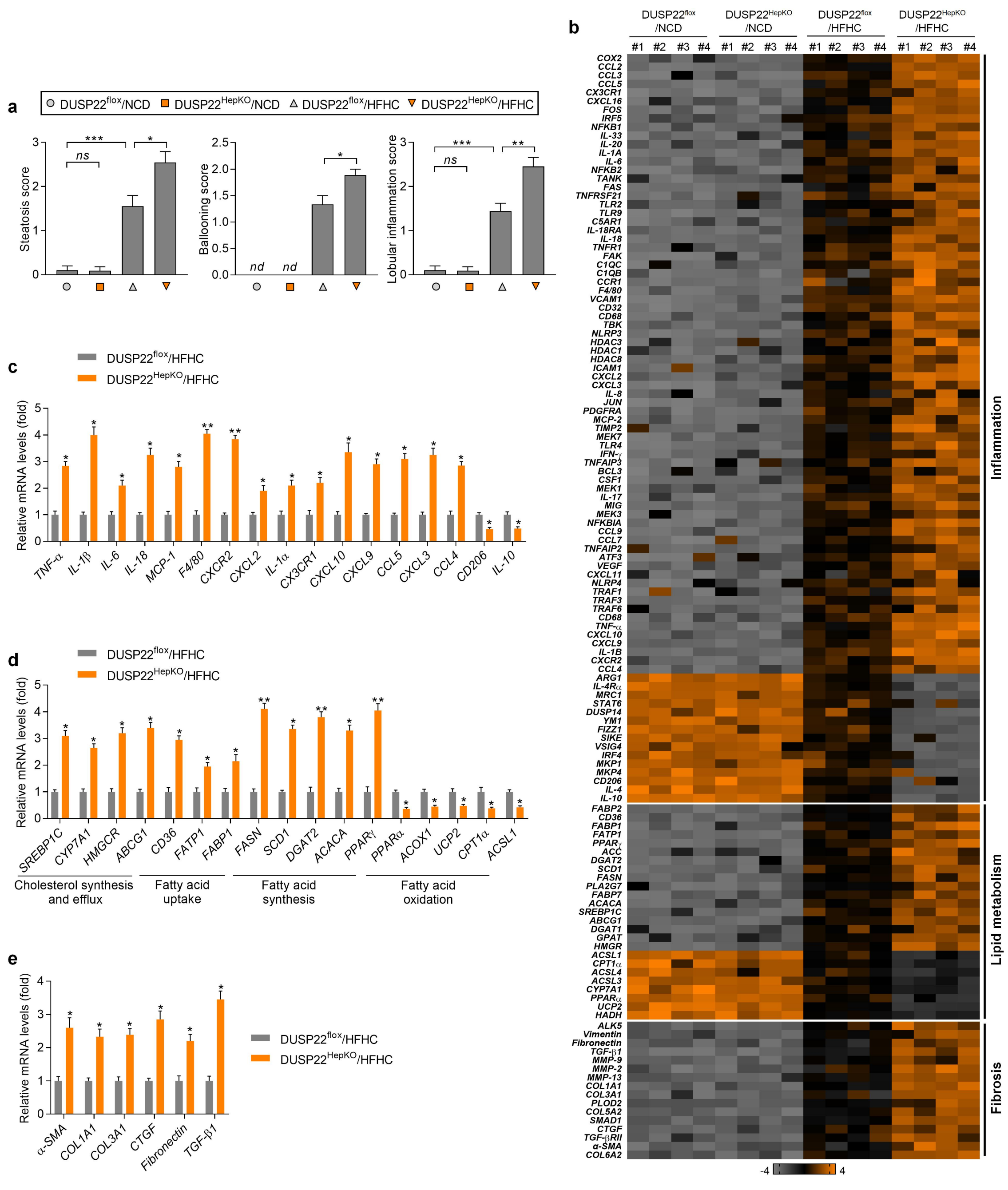
a
DUSP22flox/NCD
DUSP22HepKO/NCD
DUSP22flox/HFHC
DUSP22HepKO/HFHC
Steatosis score
Ballooning score
Lobular inflammation score
ns
***
*
**
nd
b
DUSP22flox /NCD
DUSP22HepKO /NCD
DUSP22flox /HFHC
DUSP22HepKO /HFHC
#1 #2 #3 #4
COX2
CCL2
CCL3
CCL5
CX3CR1
CXCL16
FOS
IRF5
NFKB1
IL-33
IL-20
IL-1A
IL-6
NFKB2
TANK
FAS
TNFRSF21
TLR2
TLR9
C5AR1
IL-18RA
IL-18
TNFR1
FAK
C1QC
C1QB
CCR1
F4/80
VCAM1
CD32
CD68
TBK
NLRP3
HDAC3
HDAC1
HDAC8
ICAM1
CXCL2
CXCL3
IL-8
JUN
PDGFRA
MCP-2
TIMP2
MEK7
TLR4
IFN-γ
TNFAIP3
BCL3
CSF1
MEK1
IL-17
MIG
MEK3
NFKBIA
CCL9
CCL7
TNFAIP2
ATF3
VEGF
CXCL11
NLRP4
TRAF1
TRAF3
TRAF6
CD68
TNF-α
CXCL10
CXCL9
IL-1B
CXCR2
CCL4
ARG1
IL-4Rα
MRC1
STAT6
DUSP14
YM1
FIZZ1
SIKE
VSIG4
IRF4
MKP1
MKP4
CD206
IL-4
IL-10
FABP2
CD36
FABP1
FATP1
PPARγ
ACC
DGAT2
SCD1
FASN
PLA2G7
FABP7
ACACA
SREBP1C
ABCG1
DGAT1
GPAT
HMGR
ACSL1
CPT1α
ACSL4
ACSL3
CYP7A1
PPARα
UCP2
HADH
ALK5
Vimentin
Fibronectin
TGF-β1
MMP-9
MMP-2
MMP-13
COL1A1
COL3A1
PLOD2
COL5A2
SMAD1
CTGF
TGF-βRII
α-SMA
COL6A2
Inflammation
Lipid metabolism
Fibrosis
-4
4
c
DUSP22flox/HFHC
DUSP22HepKO/HFHC
Relative mRNA levels (fold)
TNF-α
IL-1β
IL-6
IL-18
MCP-1
F4/80
CXCR2
CXCL2
IL-1α
CX3CR1
CXCL10
CXCL9
CCL5
CXCL3
CCL4
CD206
IL-10
d
DUSP22flox/HFHC
DUSP22HepKO/HFHC
Relative mRNA levels (fold)
SREBP1C
CYP7A1
HMGCR
ABCG1
CD36
FATP1
FABP1
FASN
SCD1
DGAT2
ACACA
PPARγ
PPARα
ACOX1
UCP2
CPT1α
ACSL1
Cholesterol synthesis and efflux
Fatty acid uptake
Fatty acid synthesis
Fatty acid oxidation
e
Relative mRNA levels (fold)
α-SMA
COL1A1
COL3A1
CTGF
Fibronectin
TGF-β1
DUSP22flox/HFHC
DUSP22HepKO/HFHC

Supplementary Figure 7. Hepatocyte-specific DUSP22 deficiency exacerbates HFHC-induced steatohepatitis. a Quantification for steatosis score, ballooning score and lobular inflammation score of each group of mice as shown (n=10 or 11 mice per group) ($^{*}P$<0.05, $^{**}P$<0.01 and $^{***}P$<0.001; *ns*, no significant difference). **b** High-throughput real-time PCR (HTqPCR) analysis indicating the inflammation-, lipid metabolism- and fibrosis-related genes expression alteration in liver of DUSP22^{flox} and DUSP22^{HepKO} mice fed with a NCD or a HFHC for 24 weeks (n=4 mice per group). **c** RT-qPCR analysis for the mRNA expression levels of inflammation-related genes including pro-inflammatory factors (TNF-α, IL-1β, IL-6, IL-18, MCP-1, F4/80, CXCR2, CXCL2, IL-1α, CX3CR1, CXCL10, CXCL9, CCL5, CXCL3 and CCL4) and anti-inflammatory molecules (CD206 and IL-10) in liver tissues from DUSP22^{flox} and DUSP22^{HepKO} groups of mice after a 24-week HFHC feeding (n=4 mice per group) ($^{*}P$<0.05 and $^{**}P$<0.01 versus the DUSP22^{flox}/HFHC group). **d** RT-qPCR analysis of mRNA levels for lipid metabolism-related genes in the livers of HFHC-fed DUSP22^{flox} and DUSP22^{HepKO} mice, including genes associated with cholesterol synthesis and efflux (SREBP1C, CYP7A1, HMGCR and ABCG1), fatty acid uptake (CD36, FATP1 and FABP1), fatty acid synthesis (FASN, SCD1, DGAT2, ACACA and PPARγ) and fatty acid oxidation (PPARα, ACOX1, UCP2, CPT1α and ACSL1) (n=4 mice per group) ($^{*}P$<0.05 and $^{**}P$<0.01 versus the DUSP22^{flox}/HFHC group). **e** RT-qPCR analysis for mRNA levels of fibrosis markers, including α-SMA, COL1A1, COL3A1, CTGF, Fibronectin and TGF-β1, in the livers of the indicated mice fed a HFHC for 24 weeks (n=4 mice per group) ($^{*}P$<0.05 versus the DUSP22^{flox}/HFHC group). Data are expressed as mean ± SEM from at least three independent experiments. For statistical analysis, **a** and **b** were performed by one-way ANOVA; **c-e** were conducted by two-tailed Student's *t*-test.

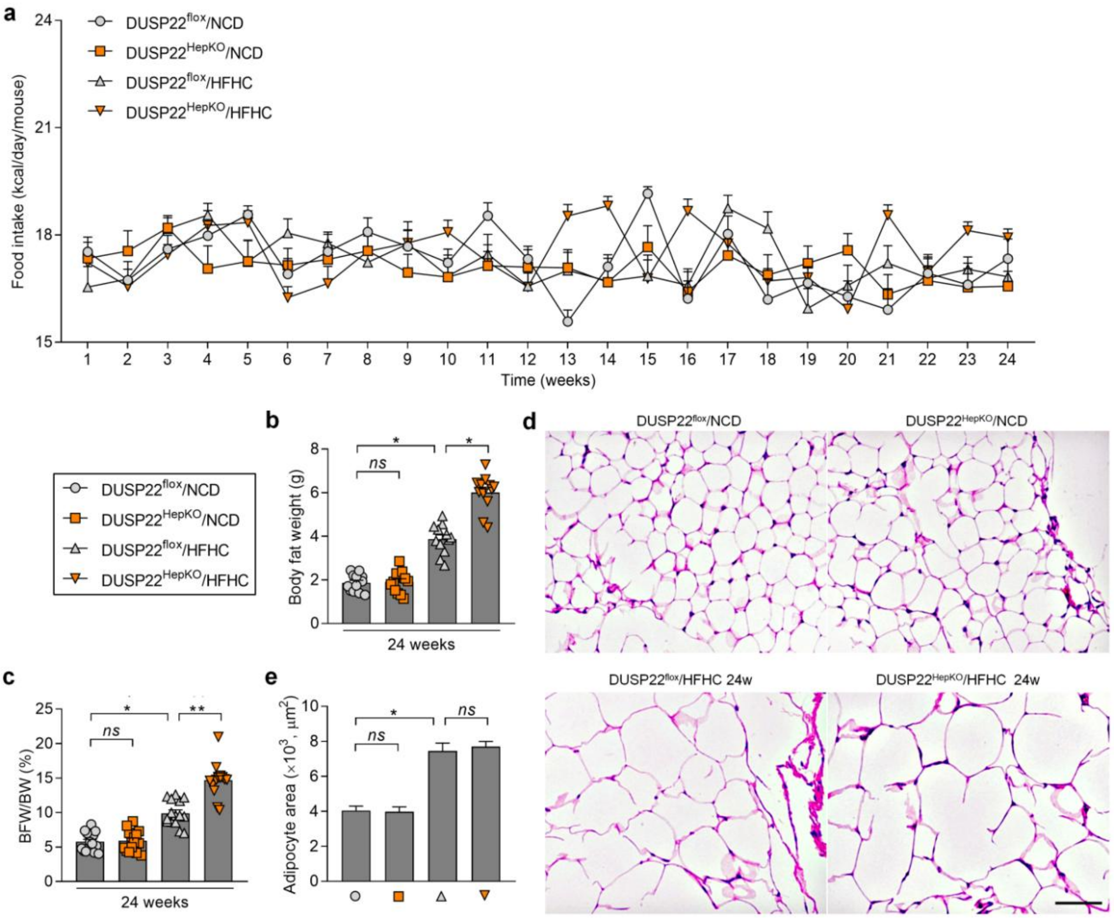

a
DUSP22flox/NCD
DUSP22HepKO/NCD
DUSP22flox/HFHC
DUSP22HepKO/HFHC
Food intake (kcal/day/mouse)
Time (weeks)
b
Body fat weight (g)
24 weeks
ns
c
BFW/BW (%)
24 weeks
ns
d
DUSP22flox/NCD
DUSP22HepKO/NCD
DUSP22flox/HFHC 24w
DUSP22HepKO/HFHC 24w
e
Adipocyte area (×10^3, μm^2)
ns

Supplementary Figure 8. Hepatocyte-specific DUSP22 knockout promotes weight gain of adipose tissue to accelerate HFHC-induced NASH pathologies. a Records of food intake (Kcal/day/per mice) of the shown groups of mice in a 24-weeks NCD or HFHC feeding (n=15 mice per group). **b,c** Measurements of (**b**) body fat weight (BFW) and (**c**) the ratio of BFW/BW (n=15 mice per group) ($^{*}P$<0.05 and $^{**}P$<0.01; *ns*, no significant difference). **d** Representative images of H&E staining of the white adipose tissue (WAT) sections of mice from the DUSP22^{flox} and DUSP22^{HepKO} groups fed with a NCD or HFHC for 24 weeks (n=8 mice per group, with 10 images for each mouse; Scale bars, 100 μm). **e** Quantification for the adipocyte area (μm^2) following (**d**) (n=8 mice per group) ($^{*}P$<0.05; *ns*, no significant difference). Data are expressed as mean ± SEM from at least three independent experiments. Statistical analysis were performed by one-way ANOVA.

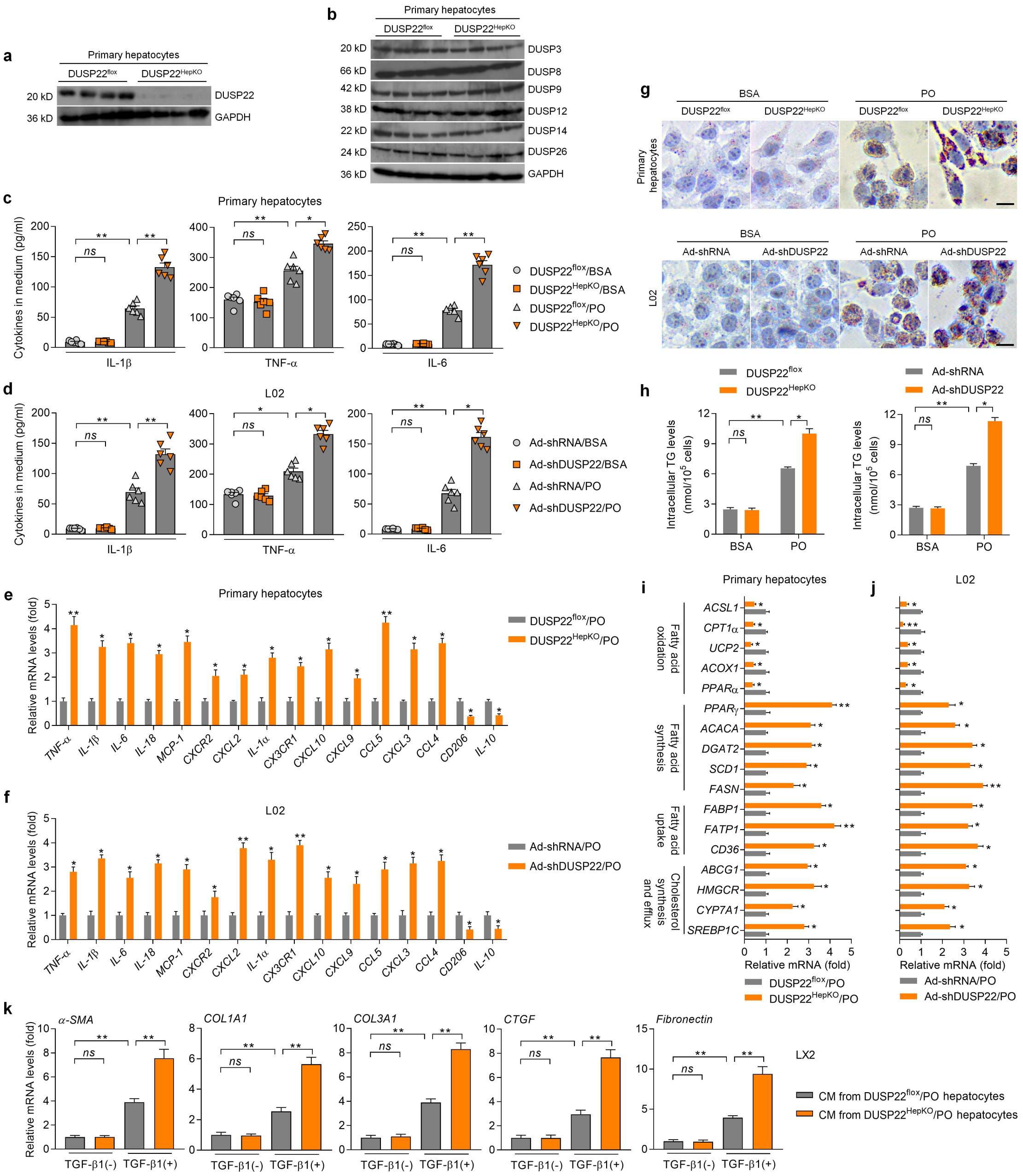

a
Primary hepatocytes
DUSP22flox
DUSP22HepKO
20 kD
DUSP22
36 kD
GAPDH
b
Primary hepatocytes
DUSP22flox
DUSP22HepKO
DUSP3
DUSP8
DUSP9
DUSP12
DUSP14
DUSP26
GAPDH
c
Primary hepatocytes
Cytokines in medium (pg/ml)
IL-1β
TNF-α
IL-6
DUSP22flox/BSA
DUSP22HepKO/BSA
DUSP22flox/PO
DUSP22HepKO/PO
d
L02
Ad-shRNA/BSA
Ad-shDUSP22/BSA
Ad-shRNA/PO
Ad-shDUSP22/PO
e
Primary hepatocytes
Relative mRNA levels (fold)
DUSP22flox/PO
DUSP22HepKO/PO
TNF-α
IL-1β
IL-6
IL-18
MCP-1
CXCR2
CXCL2
IL-1α
CX3CR1
CXCL10
CXCL9
CCL5
CXCL3
CCL4
CD206
IL-10
f
L02
Ad-shRNA/PO
Ad-shDUSP22/PO
g
BSA
PO
Primary hepatocytes
L02
Ad-shRNA
Ad-shDUSP22
h
Intracellular TG levels (nmol/10^5 cells)
i
j
Fatty acid oxidation
Fatty acid synthesis
Fatty acid uptake
Cholesterol synthesis and efflux
ACSL1
CPT1α
UCP2
ACOX1
PPARα
PPARγ
ACACA
DGAT2
SCD1
FASN
FABP1
FATP1
CD36
ABCG1
HMGCR
CYP7A1
SREBP1C
Relative mRNA (fold)
k
α-SMA
COL1A1
COL3A1
CTGF
Fibronectin
TGF-β1(-)
TGF-β1(+)
LX2
CM from DUSP22flox/PO hepatocytes
CM from DUSP22HepKO/PO hepatocytes

Supplementary Figure 9. DUSP22 decreases aggravate inflammation, lipid deposition and fibrosis *in vitro*. a,b Western blot showing (**a**) DUSP22, (**b**) DUSP3, DUSP8, DUSP9, DUSP12, DUSP14 and DUSP26 protein expression levels in the isolated primary hepatocytes from $DUSP22^{flox}$ and $DUSP22^{HepKO}$ mice (n=4 per group). **c** The concentrations of IL-1β, TNF-α and IL-6 in the culture medium of primary hepatocytes that were isolated from $DUSP22^{flox}$ and $DUSP22^{HepKO}$ mice. The hepatocytes were treated with PO (0.4 mM PA and 0.8 mM OA) for 24 h. The medium was then collected for ELISA analysis (n=6 per group) ($^{*}P<0.05$ and $^{**}P<0.01$; *ns*, no significant difference). **d** The concentrations of IL-1β, TNF-α and IL-6 in the medium of L02 cells transfected with Ad-shDUSP22 or Ad-shRNA after 24 h PO (0.4 mM PA and 0.8 mM OA) treatment (n=6 per group) ($^{*}P<0.05$ and $^{**}P<0.01$; *ns*, no significant difference). **e** RT-qPCR analysis for the mRNA expression levels of inflammation-related genes in the isolated primary hepatocytes as shown after PO (0.4 mM PA and 0.8 mM OA) incubation for 24 h (n=4 per group) ($^{*}P<0.05$ and $^{**}P<0.01$ versus the $DUSP22^{flox}$/PO group). **f** RT-qPCR analysis for the mRNA expression levels of inflammation-related genes in L02 cells with knockdown of DUSP22 after PO (0.4 mM PA and 0.8 mM OA) treatment for 24 h (n=4 per group) ($^{*}P<0.05$ and $^{**}P<0.01$ versus the Ad-shRNA/PO group). **g** Representative Oil red O staining images of the primary $DUSP22^{flox}$ and $DUSP22^{HepKO}$ hepatocytes (top) and L02 cells transfected with shRNA or shDUSP22 (bottom) after PO (0.4 mM PA and 0.8 mM OA) treatment for 24 h (n=5 per group, with 10 images per group; Scale bar, 25 μm). **h** Intracellular TG contents in the primary $DUSP22^{flox}$ and $DUSP22^{HepKO}$ hepatocytes (left) and L02 cells with or without DUSP22 knockdown (right) after PO (0.4 mM PA and 0.8 mM OA) incubation for 24 h (n=5 per group) ($^{*}P<0.05$ and $^{**}P<0.01$; *ns*, no significant difference). **i,j** RT-qPCR analysis of mRNA levels for lipid metabolism-related genes in the (**i**) primary $DUSP22^{flox}$ and $DUSP22^{HepKO}$ hepatocytes ($^{*}P<0.05$ and $^{**}P<0.01$ versus the $DUSP22^{flox}$/PO group), and (**j**) L02 cells transfected with shRNA or shDUSP22 after PO (0.4 mM PA and 0.8 mM OA) exposure for 24 h ($^{*}P<0.05$ and $^{**}P<0.01$ versus the Ad-shRNA/PO group) (n=4 per group). **k** RT-qPCR analysis of fibrosis-related genes α-SMA, COL1A1, COL3A1, CTGF and Fibronectin expression levels in 24 h TGF-β1 (10 ng/ml)-treated human hepatic stellate cell (HSC) line LX2 cells incubated with fresh medium that was mixed with CM from (**e**) at 1:1 ratio (n=4 per group) ($^{**}P<0.01$; *ns*, no significant difference). Data are expressed as mean ± SEM from at least three independent experiments. For statistical analysis, **c, d, h** and **k** were conducted by one-way ANOVA; **e, f, i** and **j** were performed by two-tailed Student's *t*-test.

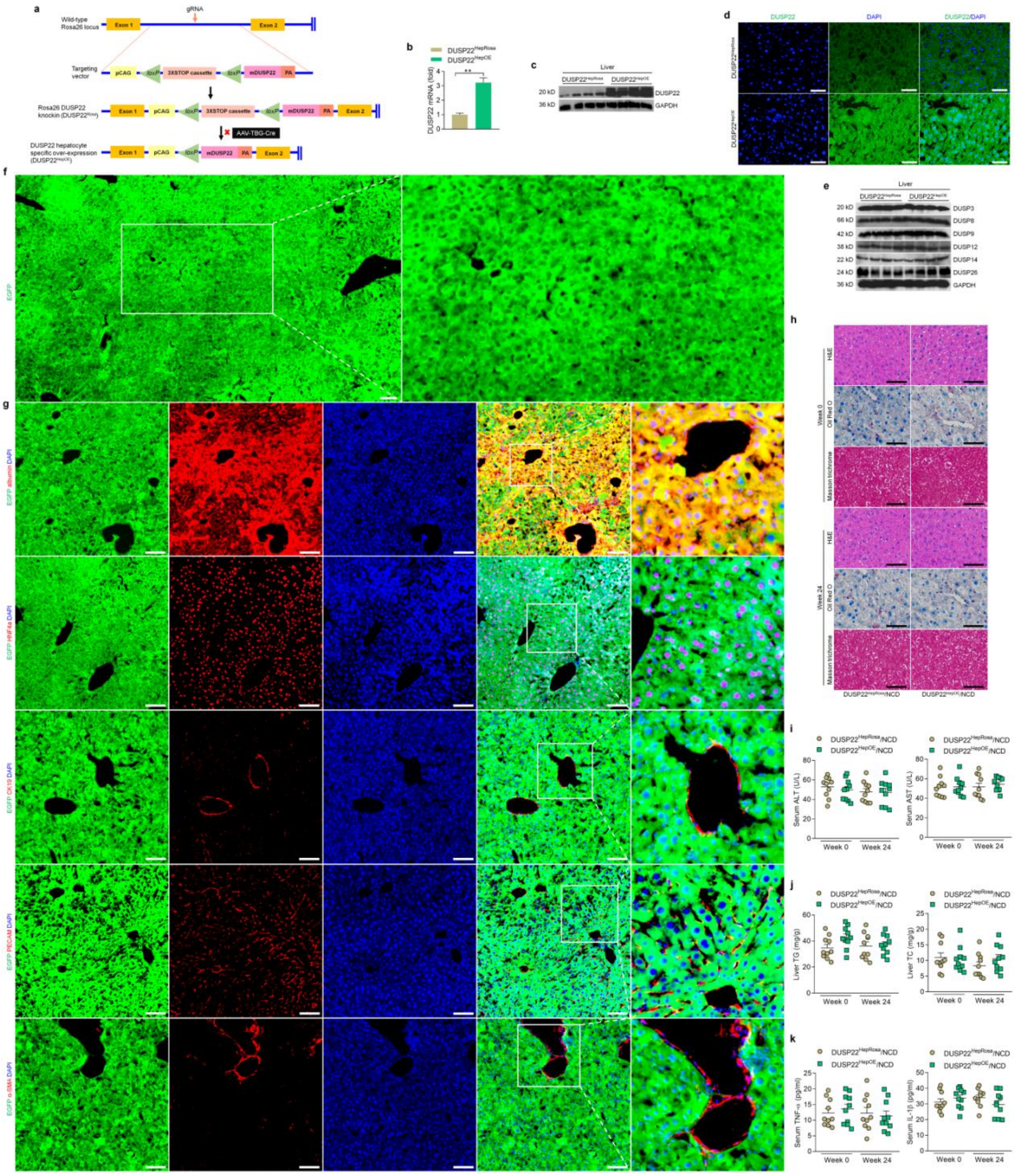

a
Wild-type Rosa26 locus
gRNA
Exon 1
Exon 2
Targeting vector
pCAG
loxP
3XSTOP cassette
mDUSP22
PA
Rosa26 DUSP22 knockin (DUSP22Rosa)
AAV-TBG-Cre
DUSP22 hepatocyte specific over-expression (DUSP22HepOE)
b
DUSP22HepRosa
DUSP22HepOE
DUSP22 mRNA (fold)
c
Liver
DUSP22
GAPDH
20 kD
36 kD
d
DUSP22
DAPI
DUSP22/DAPI
e
Liver
DUSP3
DUSP8
DUSP9
DUSP12
DUSP14
DUSP26
GAPDH
f
EGFP
g
EGFP albumin DAPI
EGFP HNF4α DAPI
EGFP CK19 DAPI
EGFP PECAM DAPI
EGFP α-SMA DAPI
h
Week 0
Week 24
H&E
Oil Red O
Masson trichrome
DUSP22HepRosa/NCD
DUSP22HepOE/NCD
i
Serum ALT (U/L)
Serum AST (U/L)
Week 0
Week 24
j
Liver TG (mg/g)
Liver TC (mg/g)
k
Serum TNF-α (pg/ml)
Serum IL-1β (pg/ml)

Supplementary Figure 10. Construction of hepatocyte-specific DUSP22 over-expression mice. a Scheme showing the establishment of hepatocyte-specific DUSP22 over-expression ($DUSP22^{HepOE}$) mouse strain. **b,c** (**b**) RT-qPCR and (**c**) western blot showing DUSP22 expression in liver from $DUSP22^{HepRosa}$ and $DUSP22^{HepOE}$ mice (n=4 mice per group) ($^{**}P$<0.01). **d** Representative immunofluorescence images of DUSP22 (green) in the livers of mice from $DUSP22^{HepRosa}$ and $DUSP22^{HepOE}$ mice, and nuclei were stained with DAPI (blue) (n=3 mice per group, with 10 images for each mouse; Scale bars, 40 μm). **e** Western blot showing DUSP3, DUSP8, DUSP9, DUSP12, DUSP14 and DUSP26 protein expression in liver from $DUSP22^{HepRosa}$ and $DUSP22^{HepOE}$ mice (n=4 mice per group). **f** Fluorescence view of the adult liver from 32-week-old AAV-TBG-Cre;$Rosa26^{DUSP22}$-EGFP mice (n=3 mice per group, with 10 images for each mouse; Scale bars, 100 μm). **g** Immunostaining for EGFP, Albumin, HNF4a, CK19, PECAM and α-SMA on liver sections showing DUSP22-expressing hepatocytes in 32-week-old AAV-TBG-Cre;$Rosa26^{DUSP22}$-EGFP mice (n=3 mice per group, with 10 images for each mouse; Scale bars, 40 μm). **h** H&E staining of liver sections from $DUSP22^{HepRosa}$ and $DUSP22^{HepOE}$ mice at 0 and 24 weeks of NCD feeding (n=8 mice per group, with 10 images for each mouse; Scale bars, 50 μm). **i-k** Quantification for (**i**) serum ALT and AST contents, (**j**) liver TG and TC levels, and (**k**) serum TNF-α and IL-1β concentrations in $DUSP22^{HepRosa}$ and $DUSP22^{HepOE}$ mice at 0 and 24 weeks of NCD feeding (n=10 per group). Data are expressed as mean ± SEM from at least three independent experiments. Statistical analysis were performed by two-tailed Student's *t*-test.

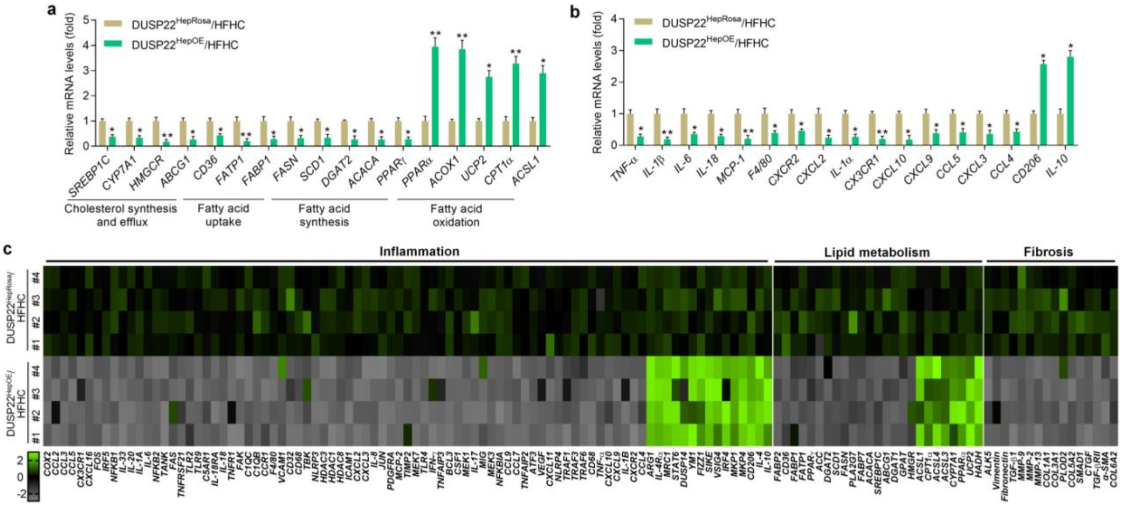
a
b
c
DUSP22^HepRosa/HFHC
DUSP22^HepOE/HFHC
Relative mRNA levels (fold)
SREBP1C
CYP7A1
HMGCR
ABCG1
CD36
FATP1
FABP1
FASN
SCD1
DGAT2
ACACA
PPARγ
PPARα
ACOX1
UCP2
CPT1α
ACSL1
Cholesterol synthesis and efflux
Fatty acid uptake
Fatty acid synthesis
Fatty acid oxidation
TNF-α
IL-1β
IL-6
IL-18
MCP-1
F4/80
CXCR2
CXCL2
IL-1α
CX3CR1
CXCL10
CXCL9
CCL5
CXCL3
CCL4
CD206
IL-10
Inflammation
Lipid metabolism
Fibrosis
#1
#2
#3
#4

Supplementary Figure 11. Hepatocyte-specific DUSP22 over-expression mitigates HFHC-induced steatohepatitis. a RT-qPCR analysis of mRNA levels for lipid metabolism-related genes in the livers of a 24-week HFHC-fed DUSP22^{HepRosa} and DUSP22^{HepOE} mice (n=4 mice per group) ($^{*}P$<0.05 and $^{**}P$<0.01 versus the DUSP22^{HepRosa}/HFHC group). **b** RT-qPCR analysis of mRNA levels for inflammation-associated genes in the livers of DUSP22^{HepRosa} and DUSP22^{HepOE} mice after HFHC feeding for 24 weeks (n=4 mice per group) ($^{*}P$<0.05 and $^{**}P$<0.01 versus the DUSP22^{HepRosa}/HFHC group). **c** HTqPCR analysis indicating the inflammation-, lipid metabolism- and fibrosis-related genes expression alteration in liver of DUSP22^{HepRosa} and DUSP22^{HepOE} mice fed with a HFHC for 24 weeks (n=4 mice per group). Data are expressed as mean ± SEM from at least three independent experiments. Statistical analysis were performed by two-tailed Student's *t*-test.

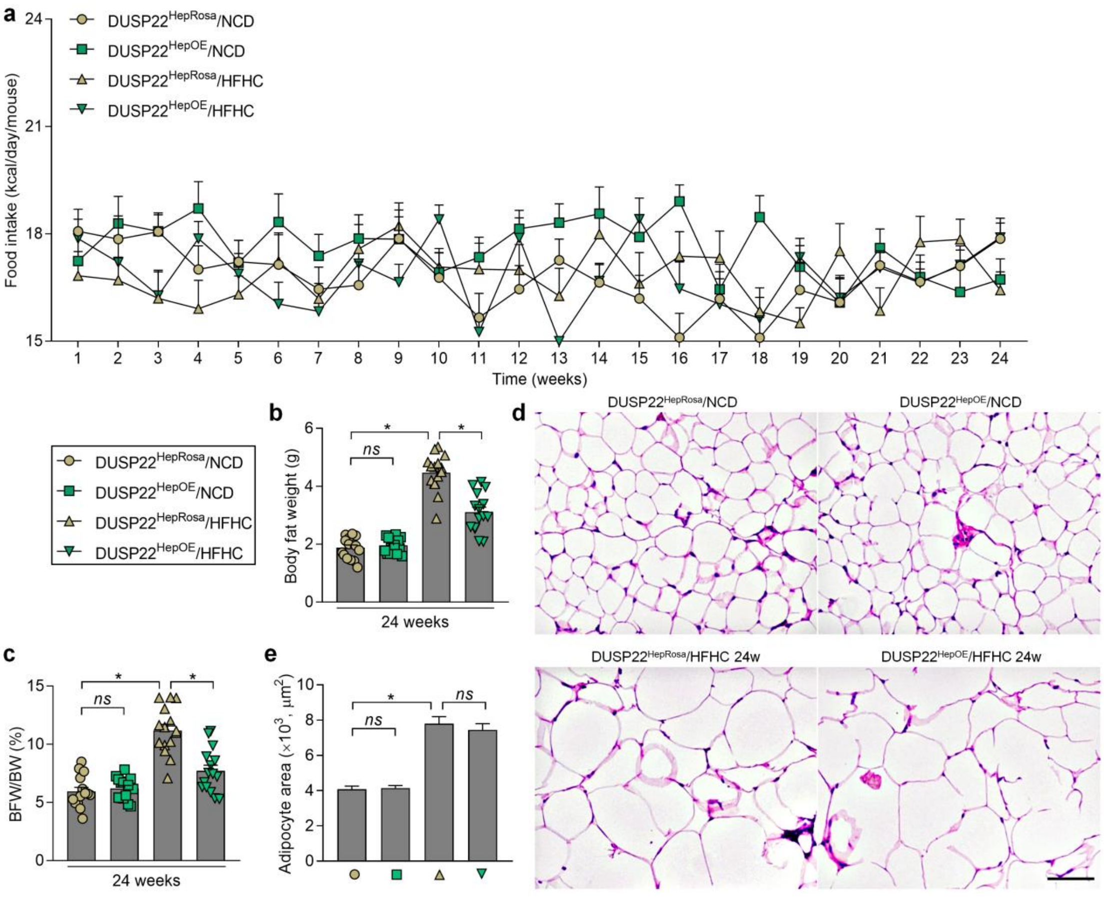

a
b
c
d
e
DUSP22^HepRosa/NCD
DUSP22^HepOE/NCD
DUSP22^HepRosa/HFHC
DUSP22^HepOE/HFHC
Food intake (kcal/day/mouse)
Time (weeks)
Body fat weight (g)
24 weeks
BFW/BW (%)
Adipocyte area (×10^3, μm^2)
ns
*
DUSP22^HepRosa/HFHC 24w
DUSP22^HepOE/HFHC 24w

Supplementary Figure 12. Hepatocyte-specific DUSP22 over-expression ameliorates weight gain of adipose tissue to alleviate HFHC-triggered NASH pathologies. a Records of food intake (Kcal/day/per mice) of the shown groups of mice with a 24-week NCD or HFHC feeding (n=15 mice per group). **b,c** Measurements of (**b**) body fat weight (BFW) and (**c**) the ratio of BFW/BW ($^{*}P<0.05$; *ns*, no significant difference). **d** Representative images of H&E staining of the WAT sections of mice from the DUSP22^{HepRosa} and DUSP22^{HepOE} groups fed with a NCD or HFHC for 24 weeks (n=8 mice per group, with 10 images for each mouse; Scale bars, 100 μm). **e** Quantification for the adipocyte area (μm^2) following (**d**) (n=8 mice per group) ($^{*}P<0.05$; *ns*, no significant difference). Data are expressed as mean ± SEM from at least three independent experiments. Statistical analysis were performed by one-way ANOVA.

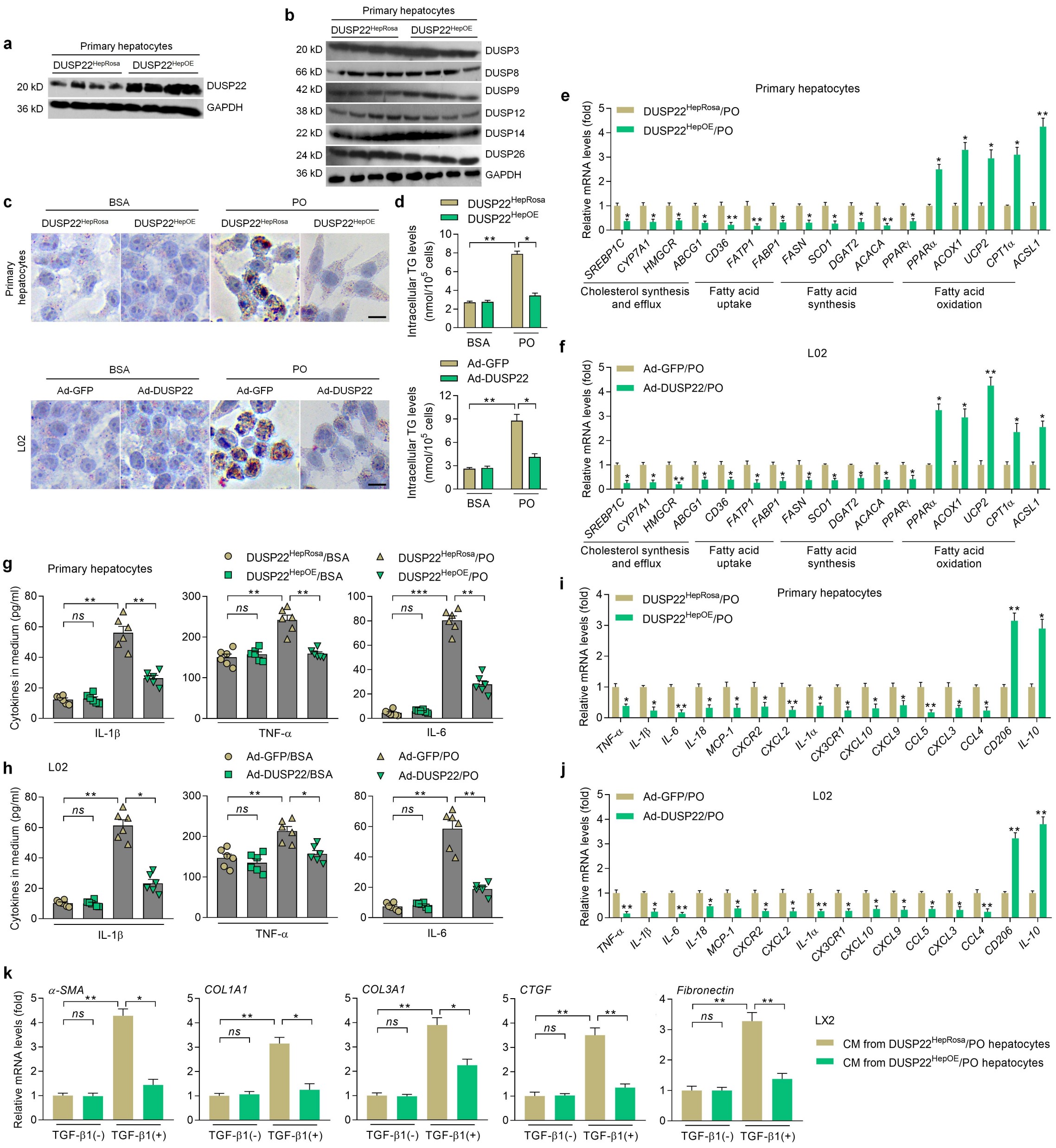

a
Primary hepatocytes
DUSP22HepRosa
DUSP22HepOE
20 kD
36 kD
DUSP22
GAPDH
b
Primary hepatocytes
DUSP22HepRosa
DUSP22HepOE
20 kD
66 kD
42 kD
38 kD
22 kD
24 kD
36 kD
DUSP3
DUSP8
DUSP9
DUSP12
DUSP14
DUSP26
GAPDH
c
BSA
PO
DUSP22HepRosa
DUSP22HepOE
Primary hepatocytes
Ad-GFP
Ad-DUSP22
L02
d
Intracellular TG levels (nmol/10^5 cells)
BSA
PO
e
Primary hepatocytes
DUSP22HepRosa/PO
DUSP22HepOE/PO
Relative mRNA levels (fold)
SREBP1C
CYP7A1
HMGCR
ABCG1
CD36
FATP1
FABP1
FASN
SCD1
DGAT2
ACACA
PPARγ
PPARα
ACOX1
UCP2
CPT1α
ACSL1
Cholesterol synthesis and efflux
Fatty acid uptake
Fatty acid synthesis
Fatty acid oxidation
f
L02
Ad-GFP/PO
Ad-DUSP22/PO
g
Primary hepatocytes
DUSP22HepRosa/BSA
DUSP22HepOE/BSA
DUSP22HepRosa/PO
DUSP22HepOE/PO
Cytokines in medium (pg/ml)
ns
IL-1β
TNF-α
IL-6
h
L02
Ad-GFP/BSA
Ad-DUSP22/BSA
Ad-GFP/PO
Ad-DUSP22/PO
i
Primary hepatocytes
TNF-α
IL-1β
IL-6
IL-18
MCP-1
CXCR2
CXCL2
IL-1α
CX3CR1
CXCL10
CXCL9
CCL5
CXCL3
CCL4
CD206
IL-10
j
L02
k
α-SMA
COL1A1
COL3A1
CTGF
Fibronectin
TGF-β1(-)
TGF-β1(+)
LX2
CM from DUSP22HepRosa/PO hepatocytes
CM from DUSP22HepOE/PO hepatocytes

Supplementary Figure 13. Increases of DUSP22 mitigates inflammation, lipid deposition and fibrosis *in vitro*. a,b Western blot showing (**a**) DUSP22, (**b**) DUSP3, DUSP8, DUSP9, DUSP12, DUSP14 and DUSP26 protein expression in the isolated primary hepatocytes from $DUSP22^{HepRosa}$ and $DUSP22^{HepOE}$ mice (n=4 per group). **c** Representative Oil red O staining images of the primary $DUSP22^{HepRosa}$ and $DUSP22^{HepOE}$ hepatocytes (top) and L02 cells transfected with Ad-GFP or Ad-DUSP22 (bottom) after PO (0.4 mM PA and 0.8 mM OA) treatment for 24 h (n=5 per group, with 10 images per group; Scale bar, 25 μm). **d** Intracellular TG contents in the primary $DUSP22^{HepRosa}$ and $DUSP22^{HepOE}$ hepatocytes (top) and L02 cells with or without DUSP22 over-expression (bottom) after PO (0.4 mM PA and 0.8 mM OA) incubation for 24 h (n=5 per group) ($^{*}P<0.05$ and $^{**}P<0.01$). **e** RT-qPCR analysis for the mRNA expression levels of lipid metabolism-associated genes in the isolated primary hepatocytes as shown after PO (0.4 mM PA and 0.8 mM OA) exposure for 24 h (n=4 per group) ($^{*}P<0.05$ and $^{**}P<0.01$ versus the $DUSP22^{HepRosa}$/PO group). **f** RT-qPCR analysis for the mRNA expression levels of lipid metabolism-related genes in L02 cells transfected with Ad-GFP or Ad-DUSP22 following PO (0.4 mM PA and 0.8 mM OA) treatment for 24 h (n=4 per group) ($^{*}P<0.05$ and $^{**}P<0.01$ versus the Ad-GFP/PO group). **g** The concentrations of IL-1β, TNF-α and IL-6 in the culture medium of primary hepatocytes that were isolated from $DUSP22^{HepRosa}$ and $DUSP22^{HepOE}$ mice. The hepatocytes were then treated with PO (0.4 mM PA and 0.8 mM OA) for 24 h. The medium was collected for ELISA assay (n=6 per group) ($^{**}P<0.01$ and $^{***}P<0.001$; *ns*, no significant difference). **h** The concentrations of IL-1β, TNF-α and IL-6 in the medium of L02 cells with or without DUSP22 over-expression post 24 h PO (0.4 mM PA and 0.8 mM OA) treatment (n=6 per group) ($^{*}P<0.05$ and $^{**}P<0.01$; *ns*, no significant difference). **i,j** RT-qPCR analysis of mRNA levels for inflammation-related genes in the (**i**) primary hepatocytes isolated from $DUSP22^{HepRosa}$ and $DUSP22^{HepOE}$ mice ($^{*}P<0.05$ and $^{**}P<0.01$ versus the $DUSP22^{HepRosa}$/PO group), and (**j**) L02 cells transfected with Ad-GFP or Ad-DUSP22 after PO (0.4 mM PA and 0.8 mM OA) incubation for 24 h ($^{*}P<0.05$ and $^{**}P<0.01$ versus the Ad-GFP/PO group) (n=4 per group). **k** RT-qPCR analysis of fibrosis-associated genes expression levels in 24 h of TGF-β1 (10 ng/ml)-stimulated LX2 cells that were incubated with the mix of fresh medium and CM from (**e**) at 1:1 ratio (n=4 per group) ($^{*}P<0.05$ and $^{**}P<0.01$; *ns*, no significant difference). Data are expressed as mean ± SEM from at least three independent experiments. For statistical analysis, **d, g, h** and **k** were conducted by one-way ANOVA; **e, f, i** and **j** were performed by two-tailed Student's *t*-test.

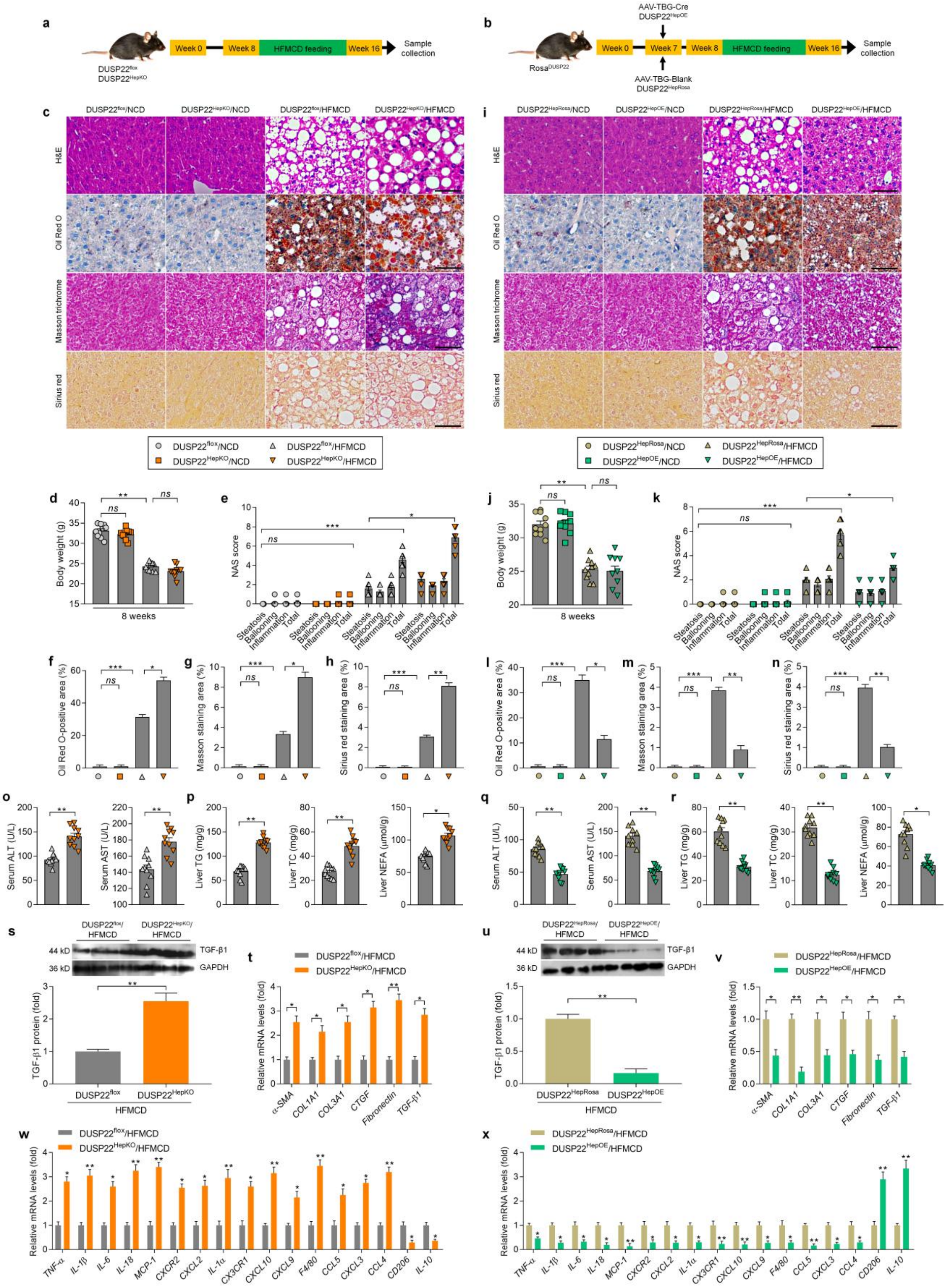

a
DUSP22flox
DUSP22HepKO
Week 0
Week 8
HFMCD feeding
Week 16
Sample collection
b
RosaDUSP22
AAV-TBG-Cre
DUSP22HepOE
AAV-TBG-Blank
DUSP22HepRosa
Week 0
Week 7
Week 8
HFMCD feeding
Week 16
Sample collection
c
DUSP22flox/NCD
DUSP22HepKO/NCD
DUSP22flox/HFMCD
DUSP22HepKO/HFMCD
H&E
Oil Red O
Masson trichrome
Sirius red
i
DUSP22HepRosa/NCD
DUSP22HepOE/NCD
DUSP22HepRosa/HFMCD
DUSP22HepOE/HFMCD
d
Body weight (g)
8 weeks
e
NAS score
Steatosis
Ballooning
Inflammation
Total
j
k
f
Oil Red O-positive area (%)
g
Masson staining area (%)
h
Sirius red staining area (%)
l
m
n
o
Serum ALT (U/L)
Serum AST (U/L)
p
Liver TG (mg/g)
Liver TC (mg/g)
Liver NEFA (μmol/g)
q
r
s
44 kD
36 kD
TGF-β1
GAPDH
TGF-β1 protein (fold)
HFMCD
t
Relative mRNA levels (fold)
α-SMA
COL1A1
COL3A1
CTGF
Fibronectin
TGF-β1
u
v
w
DUSP22flox/HFMCD
DUSP22HepKO/HFMCD
TNF-α
IL-1β
IL-6
IL-18
MCP-1
CXCR2
CXCL2
IL-1α
CX3CR1
CXCL10
CXCL9
F4/80
CCL5
CXCL3
CCL4
CD206
IL-10
x
DUSP22HepRosa/HFMCD
DUSP22HepOE/HFMCD
ns

Supplementary Figure 14. Hepatocyte DUSP22 ameliorates HFMCD diet-induced NASH phenotypes *in vivo*. a Scheme showing hepatocyte-specific DUSP22 knockout mice with an 8-week NCD or HFMCD feeding ($DUSP22^{HepKO}$ NCD; $DUSP22^{HepKO}$ HFMCD). The $DUSP22^{flox}$ was used as control ($DUSP22^{flox}$ NCD; $DUSP22^{flox}$ HFMCD). **b** Schematic diagram of AAV-TBG-Cre-mediated DUSP22 over-expression in liver of an 8-week NCD or HFMCD diet-fed RosaDUSP22 mice ($DUSP22^{HepOE}$ NCD; $DUSP22^{HepOE}$ HFMCD). The AAV-TBG-blank was used as control ($DUSP22^{HepRosa}$ NCD; $DUSP22^{HepRosa}$ HFMCD). **c** Representative images of H&E staining, Oil Red O staining, Masson trichrome staining and Sirius red staining in liver sections from $DUSP22^{flox}$ and $DUSP22^{HepKO}$ mice fed with a NCD or a HFMCD for 8 weeks (n=6 or 10 mice per group, with 10 images for each mouse; Scale bars, 50 μm). **d** Measurements of body weights of mice from the shown groups of mice (n=10 mice per group) ($^{**}P$<0.01; *ns*, no significant difference). **e-h** Results for (**e**) NAS score, (**f**) Oil Red O-positive staining, (**g**) Masson trichrome-positive staining, and (**h**) Sirius red-positive staining were analyzed and quantified in the shown groups of mice (n=6 or 10 mice per group) ($^{*}P$<0.05, $^{**}P$<0.01 and $^{***}P$<0.001; *ns*, no significant difference). **i** Representative images of H&E staining, Oil Red O staining, Masson trichrome staining and Sirius red staining in liver sections from $DUSP22^{HepRosa}$ and $DUSP22^{HepOE}$ mice fed with a NCD or a HFMCD for 8 weeks (n=6 or 10 mice per group, with 10 images for each mouse; Scale bars, 50 μm). **j** Measurements of body weights of mice from the indicated groups of mice (n=10 mice per group) ($^{**}P$<0.01; *ns*, no significant difference). **k-n** Results for (**k**) NAS score, (**l**) Oil Red O-positive staining, (**m**) Masson trichrome-positive staining, and (**n**) Sirius red-positive staining were analyzed and quantified in the indicated groups of mice (n=6 or 10 mice per group) ($^{*}P$<0.05, $^{**}P$<0.01 and $^{***}P$<0.001; *ns*, no significant difference). **o,p** Contents of (**o**) serum ALT and AST, and (**p**) liver TG, TC and NEFA were assessed in $DUSP22^{flox}$ and $DUSP22^{HepKO}$ mice fed with a HFMCD for 8 weeks (n=10 mice per group) ($^{*}P$<0.05 and $^{**}P$<0.01). **q,r** Contents of (**q**) serum ALT and AST, and (**r**) liver TG, TC and NEFA were assessed in $DUSP22^{HepRosa}$ and $DUSP22^{HepOE}$ mice fed with a HFMCD for 8 weeks (n=10 mice per group) ($^{*}P$<0.05 and $^{**}P$<0.01). **s** Representative western blotting and quantification of the protein expression of TGF-β1 in liver of $DUSP22^{flox}$ and $DUSP22^{HepKO}$ mice fed with a HFMCD for 8 weeks (n=4 mice per group) ($^{**}P$<0.01). **t** RT-qPCR results for mRNA levels of fibrosis-related genes in liver of the shown groups of mice (n=4 mice per group) ($^{*}P$<0.05 and $^{**}P$<0.01). **u** Representative western blotting of TGF-β1 in liver of $DUSP22^{HepRosa}$ and $DUSP22^{HepOE}$ mice fed with a HFMCD for 8 weeks (n=4 mice per group) ($^{**}P$<0.01). **v** RT-qPCR results for mRNA levels of fibrosis-related genes in liver of the indicated groups of mice (n=4 mice per group) ($^{*}P$<0.05 and $^{**}P$<0.01). **w,x** RT-qPCR results

for inflammation-related genes in liver of (**w**) DUSP22^{flox} and DUSP22^{HepKO} mice ($^{*}P$<0.05 and $^{**}P$<0.01 versus the DUSP22^{flox}/HFMCD group), and (**x**) DUSP22^{HepRosa} and DUSP22^{HepOE} mice fed with HFMCD for 8 weeks ($^{*}P$<0.05 and $^{**}P$<0.01 versus the DUSP22^{HepRosa}/HFMCD group) (n=4 mice per group). Data are expressed as mean ± SEM from at least three independent experiments. Statistical analysis were conducted by two-tailed Student's *t*-test.

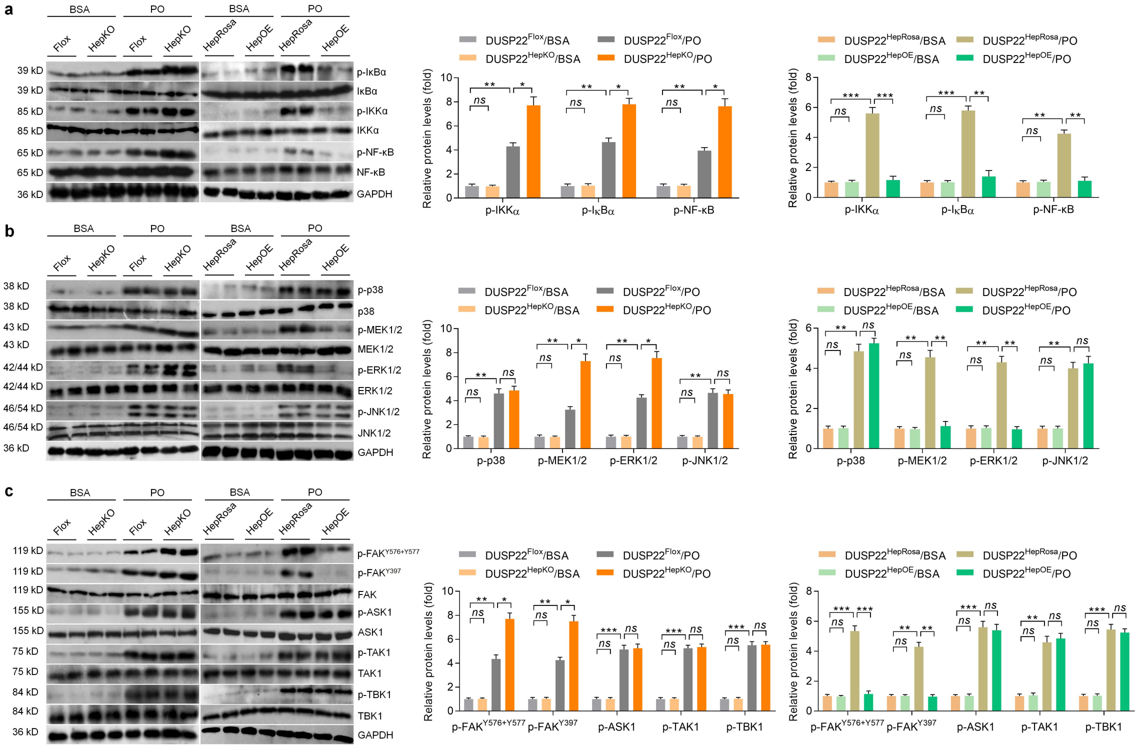
a
b
c
BSA
PO
Flox
HepKO
HepRosa
HepOE
p-IκBα
IκBα
p-IKKα
IKKα
p-NF-κB
NF-κB
GAPDH
p-p38
p38
p-MEK1/2
MEK1/2
p-ERK1/2
ERK1/2
p-JNK1/2
JNK1/2
p-FAK Y576+Y577
p-FAK Y397
FAK
p-ASK1
ASK1
p-TAK1
TAK1
p-TBK1
TBK1
DUSP22 Flox/BSA
DUSP22 Flox/PO
DUSP22 HepKO/BSA
DUSP22 HepKO/PO
DUSP22 HepRosa/BSA
DUSP22 HepRosa/PO
DUSP22 HepOE/BSA
DUSP22 HepOE/PO
Relative protein levels (fold)

Supplementary Figure 15. DUSP22 regulates the activation of NF-κB and FAK signaling *in vitro*. a Representative western blotting and quantification of the protein expression of total and phosphorylated IKKα, IκBα and NF-κB in the 24 h PO (0.4 mM PA and 0.8 mM OA)-treated primary hepatocytes isolated from $DUSP22^{flox}$, $DUSP22^{HepKO}$, $DUSP22^{HepRosa}$ and $DUSP22^{HepOE}$ mice as shown ($^{*}P$<0.05, $^{**}P$<0.01 and $^{***}P$<0.001; *ns*, no significant difference). **b** Representative western blotting of total and phosphorylated p38, MEK1/2, ERK1/2 and JNK1/2 protein expression levels in the primary hepatocytes from the mice as displayed after PO (0.4 mM PA and 0.8 mM OA) treatment for 24 h ($^{*}P$<0.05 and $^{**}P$<0.01; *ns*, no significant difference). **c** Representative western blotting and quantification of the protein expression of total and phosphorylated $FAK^{Y576+Y577}$, FAK^{Y397}, ASK1, TAK1 and TBK1 in the primary hepatocytes from the mice as displayed after PO (0.4 mM PA and 0.8 mM OA) incubation for 24 h ($^{*}P$<0.05, $^{**}P$<0.01 and $^{***}P$<0.001; *ns*, no significant difference). Data are expressed as mean ± SEM from at least three independent experiments (n=4 per group). Statistical analysis was carried out by two-tailed Student's *t*-test.

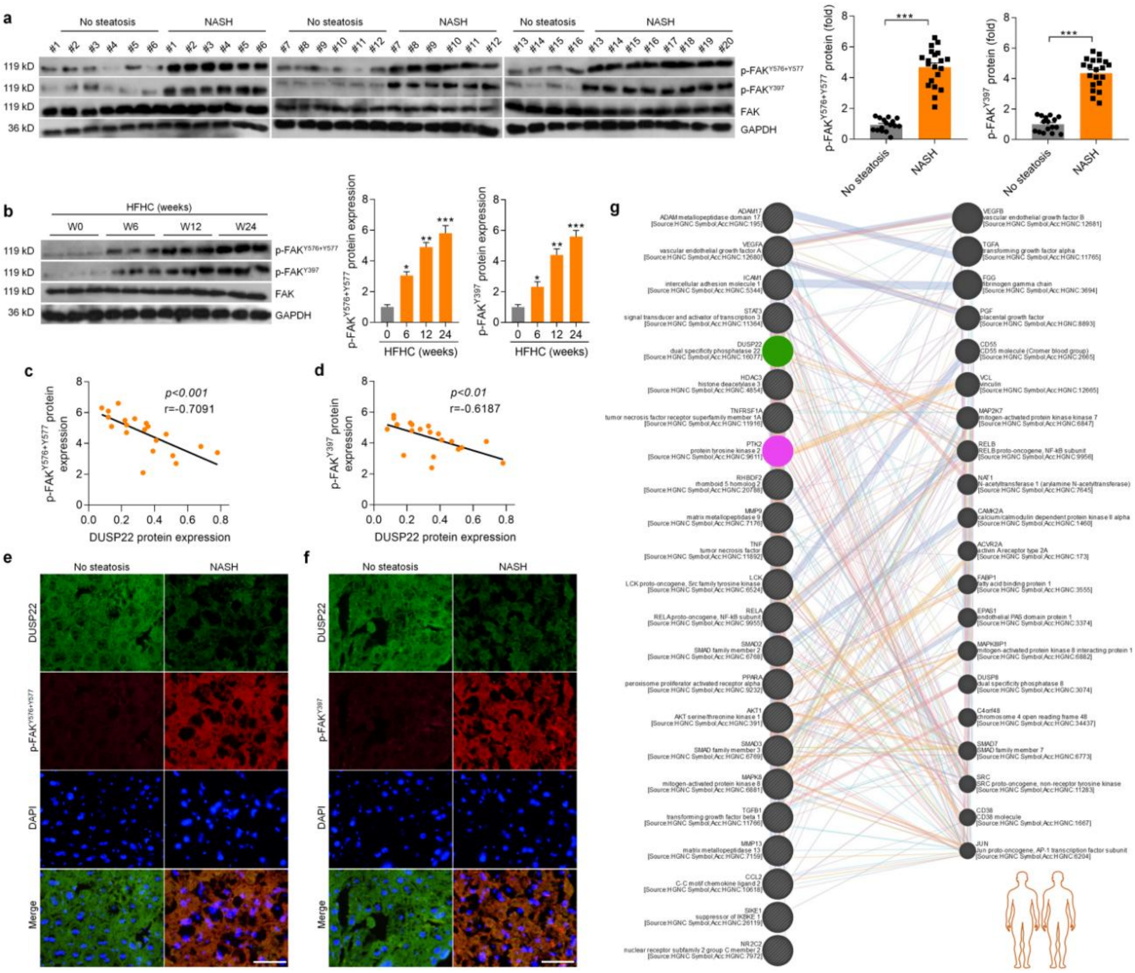
a
No steatosis
NASH
p-FAK Y576+Y577
p-FAK Y397
FAK
GAPDH
119 kD
36 kD
No steatosis
NASH
b
HFHC (weeks)
W0
W6
W12
W24
HFHC (weeks)
c
p<0.001
r=-0.7091
DUSP22 protein expression
d
p<0.01
r=-0.6187
e
f
DUSP22
DAPI
Merge
g

Supplementary Figure 16. FAK activation is up-regulated in livers with hepatic steatosis. a Representative western blotting analysis and quantification of total FAK and phosphorylated $FAK^{Y576+Y577}$ and FAK^{Y397} protein expression in livers of individuals without (No steatosis; n=16) or with NASH (n=20) ($^{***}P<0.001$). **b** Western blotting analysis for total FAK and phosphorylated $FAK^{Y576+Y577}$ and FAK^{Y397} protein levels in the livers of WT C57BL/6N mice fed a HFHC at the indicated time of weeks (n=3 per group) ($^{*}P<0.05$, $^{**}P<0.01$ and $^{***}P<0.001$ versus the expression at HFHC-0-week group). **c** Pearson comparison analysis of the correlation between DUSP22 protein level and phosphorylated $FAK^{Y576+Y577}$ protein expression levels in NASH patients (n=20). **d** Pearson comparison analysis of the correlation between DUSP22 protein level and phosphorylated FAK^{Y397} protein expression levels in NASH patients (n=20). **e** Representative immunofluorescence images of phosphorylated $FAK^{Y576+Y577}$ (red) and DUSP22 (green) co-expression in liver tissue of individuals with non-steatosis or NASH phenotype (n=8 per group, with 10 images for each sample; Scale bars, 50 μm). **f** Representative immunofluorescence images of phosphorylated FAK^{Y397} (red) and DUSP22 (green) co-expression in liver tissue of individuals with non-steatosis or NASH phenotype (n=8 per group, with 10 images for each sample; Scale bars, 50 μm). **g** GeneMANIA predicts the possible protein interaction network between human DUSP22 and FAK (PTK2). Data are expressed as mean ± SEM from at least three independent experiments. For statistical analysis, **a** was performed by two-tailed Student's *t*-test; **b** was performed by one-way ANOVA.

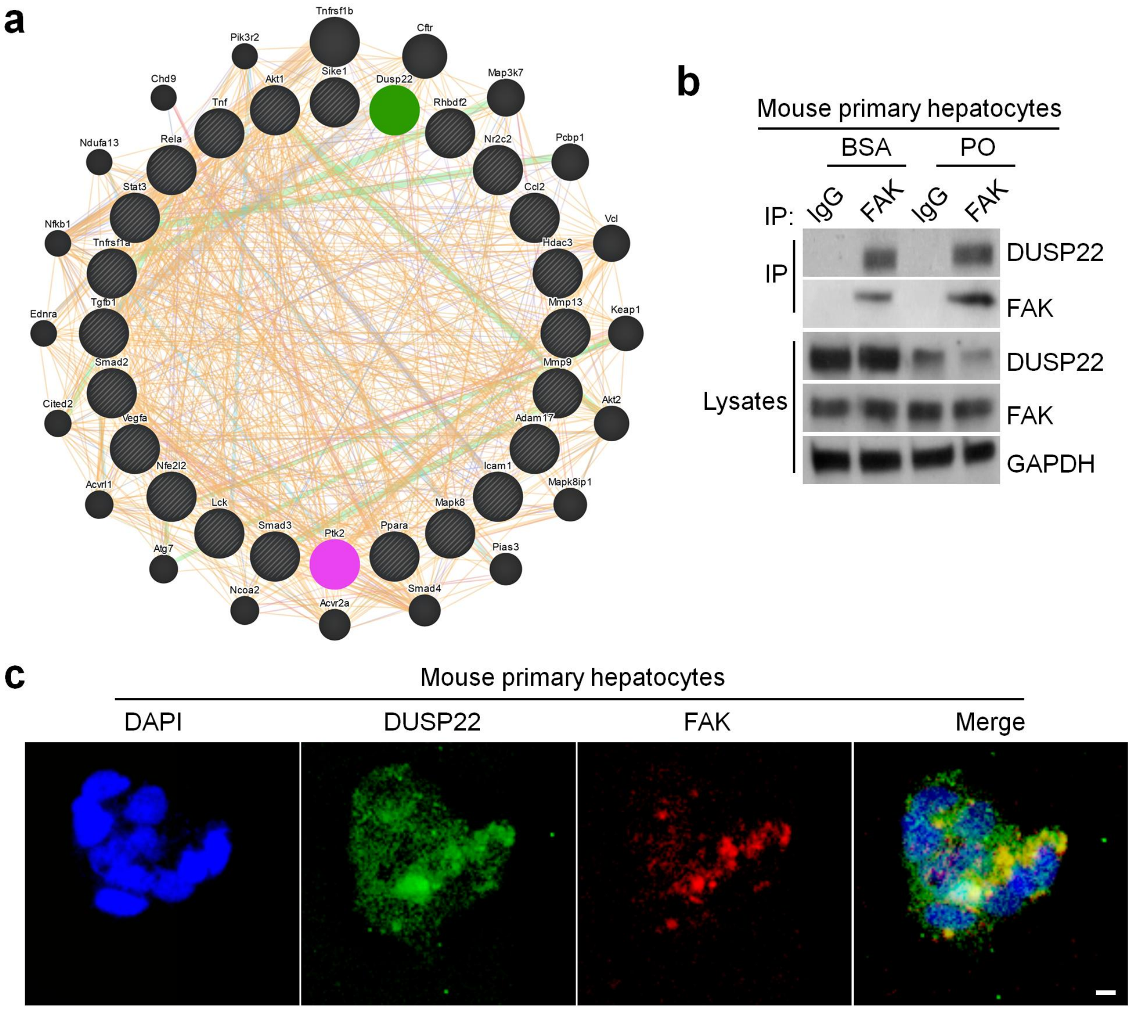
a
Tnfrsf1b
Pik3r2
Cftr
Chd9
Akt1
Sike1
Dusp22
Map3k7
Tnf
Rhbdf2
Ndufa13
Rela
Nr2c2
Pcbp1
Stat3
Ccl2
Nfkb1
Vcl
Tnfrsf1a
Hdac3
Ednra
Tgfb1
Mmp13
Keap1
Smad2
Mmp9
Cited2
Vegfa
Akt2
Adam17
Nfe2l2
Icam1
Acvrl1
Mapk8ip1
Lck
Mapk8
Smad3
Ptk2
Ppara
Atg7
Pias3
Ncoa2
Smad4
Acvr2a
b
Mouse primary hepatocytes
BSA
PO
IP:
IgG
FAK
IgG
FAK
IP
DUSP22
FAK
Lysates
DUSP22
FAK
GAPDH
c
Mouse primary hepatocytes
DAPI
DUSP22
FAK
Merge

Supplementary Figure 17. DUSP22 interacts with FAK in mouse. a GeneMANIA predicts the possible protein interaction network between mouse DUSP22 and FAK (PTK2). **b** Results from endogenous Co-IP of DUSP22 with FAK in primary mouse hepatocytes stimulated with PO (0.4 mM PA and 0.8 mM OA) or vehicle (BSA) for 24 h. IgG was used as a control. **c** Representative immunofluorescent staining of primary mouse hepatocytes co-transfected with Flag-tagged DUSP22 (green) and HA-tagged FAK (red) after transfection for 24 h (n=10 images in total; Scale bar, 10 μm). Data are expressed as mean ± SEM from at least three independent experiments.

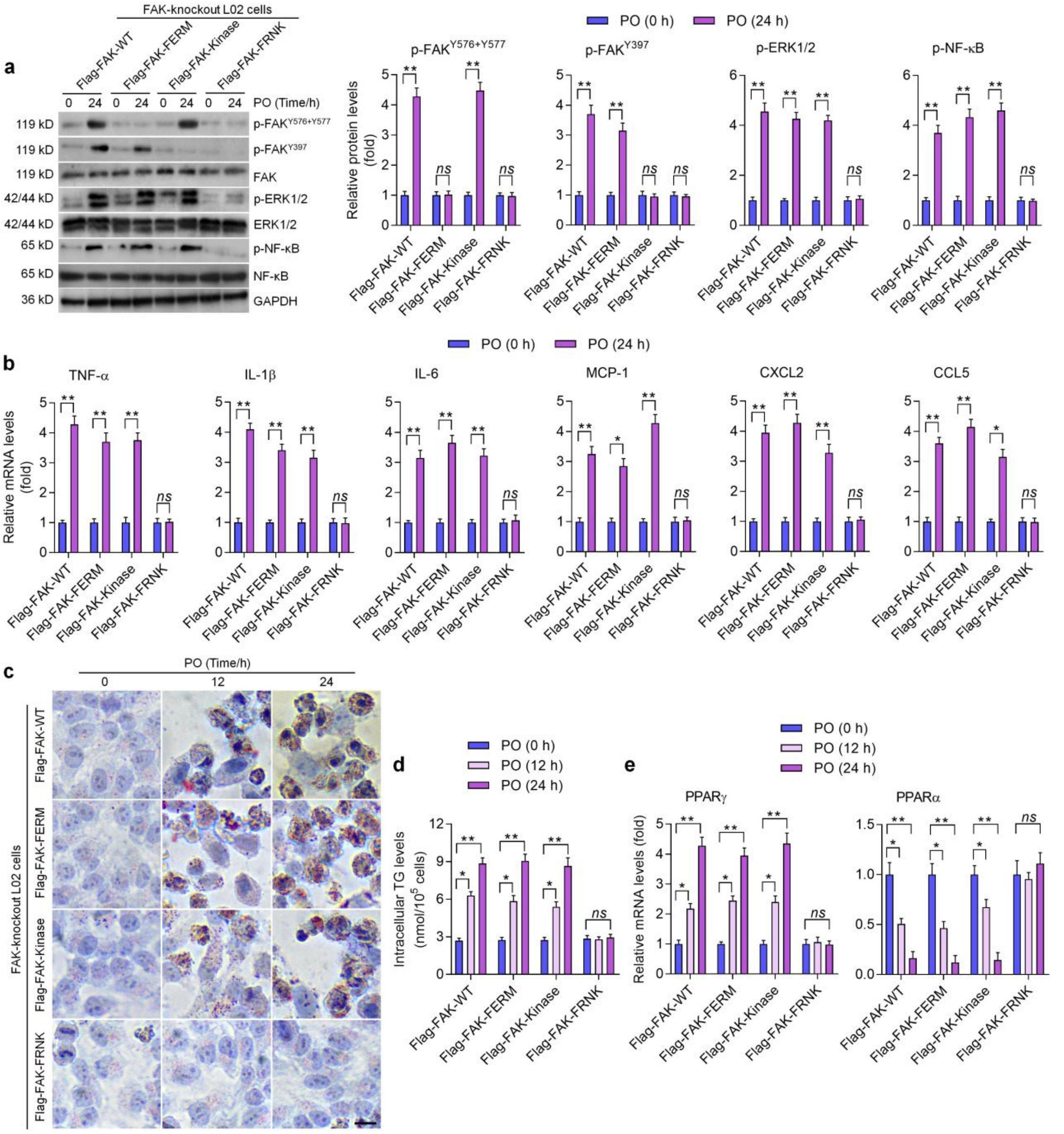

a
FAK-knockout L02 cells
Flag-FAK-WT
Flag-FAK-FERM
Flag-FAK-Kinase
Flag-FAK-FRNK
PO (Time/h)
0 24 0 24 0 24 0 24
119 kD p-FAK Y576+Y577
119 kD p-FAK Y397
119 kD FAK
42/44 kD p-ERK1/2
42/44 kD ERK1/2
65 kD p-NF-κB
65 kD NF-κB
36 kD GAPDH
PO (0 h)
PO (24 h)
Relative protein levels (fold)
p-FAK Y576+Y577
p-FAK Y397
p-ERK1/2
p-NF-κB
b
Relative mRNA levels (fold)
TNF-α
IL-1β
IL-6
MCP-1
CXCL2
CCL5
c
PO (Time/h)
0
12
24
FAK-knockout L02 cells
d
PO (12 h)
Intracellular TG levels (nmol/10^5 cells)
e
Relative mRNA levels (fold)
PPARγ
PPARα
ns

Supplementary Figure 18. Both FAK (FERM) and FAK (Kinase) promote NASH *in vitro*. a Representative western blot analysis of phosphorylated and total FAK, ERK1/2 and NF-κB in the FAK-knockout L02 cells transfected with the Flag-FAK-WT, Flag-FAK-FERM, Flag-FAK-Kinase or Flag-FAK-FRNK plasmids after PO (0.4 mM PA and 0.8 mM OA) challenge for 0 h and 24 h ($^{**}P$<0.01; *ns*, no significant difference). **b** RT-qPCR results for inflammation-related genes as shown in the FAK-knockout L02 cells transfected with the presented plasmids after PO (0.4 mM PA and 0.8 mM OA) incubation for 0 h and 24 h ($^{*}P$<0.05 and $^{**}P$<0.01; *ns*, no significant difference). **c,d** Representative Oil red O staining images (**c**) and intracellular TG contents (**d**) in FAK-knockout L02 cell line transfected with the shown plasmids following PO (0.4 mM PA and 0.8 mM OA) treatment for 0 h, 12 h and 24 h (10 images per group; Scale bar, 25 μm) ($^{*}P$<0.05 and $^{**}P$<0.01; *ns*, no significant difference). **e** RT-qPCR results for PPARγ and PPARα in the FAK-knockout L02 cells transfected with the indicated plasmids after PO (0.4 mM PA and 0.8 mM OA) exposure for 0 h, 12 h and 24 h ($^{*}P$<0.05 and $^{**}P$<0.01; *ns*, no significant difference). Data are expressed as mean ± SEM from at least three independent experiments (n=4 per group). Statistical analysis were performed by two-tailed Student's *t*-test.

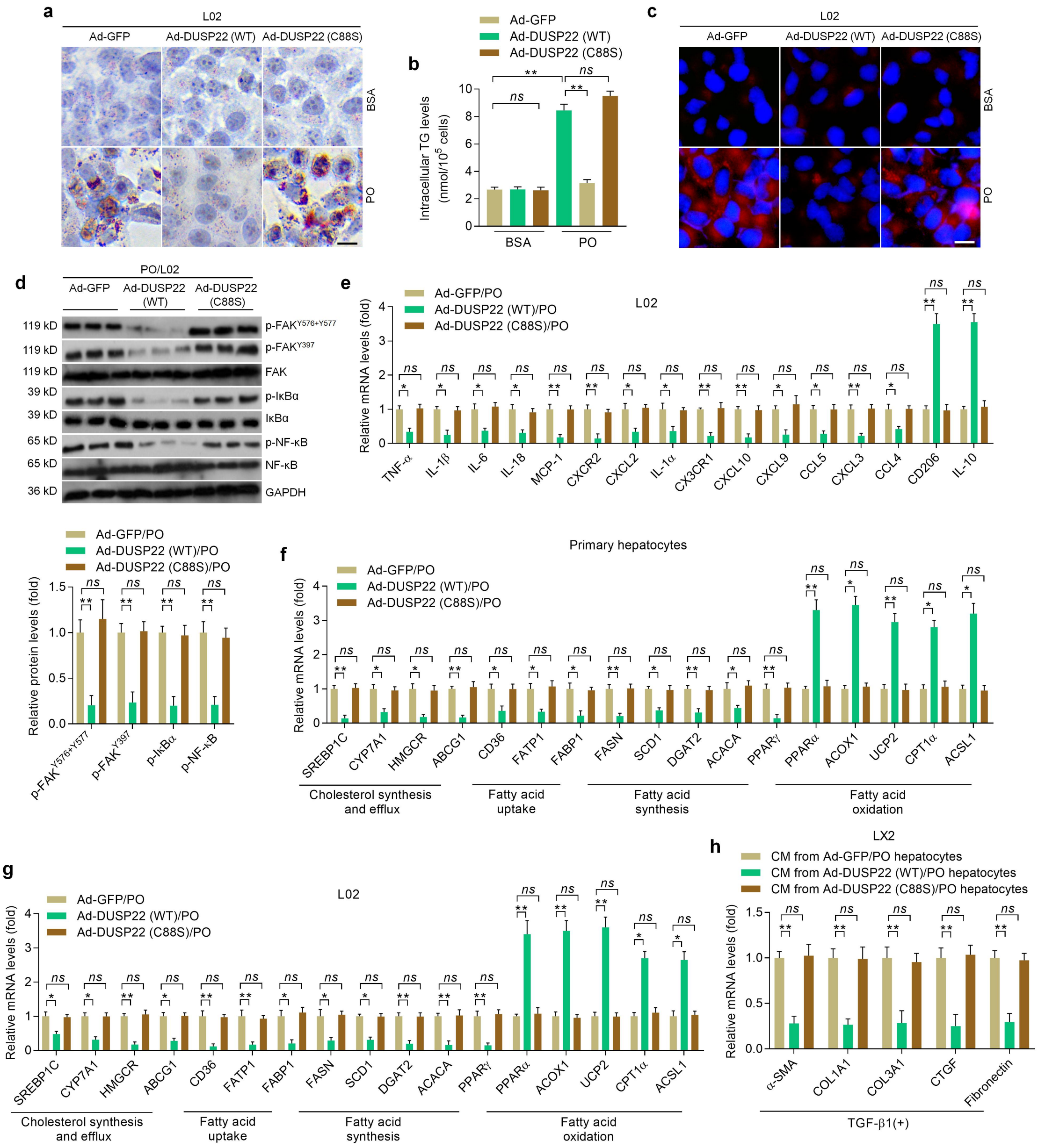
a
L02
Ad-GFP
Ad-DUSP22 (WT)
Ad-DUSP22 (C88S)
BSA
PO
b
Ad-GFP
Ad-DUSP22 (WT)
Ad-DUSP22 (C88S)
Intracellular TG levels (nmol/10^5 cells)
ns
**
BSA
PO
c
L02
Ad-GFP
Ad-DUSP22 (WT)
Ad-DUSP22 (C88S)
BSA
PO
d
PO/L02
Ad-GFP
Ad-DUSP22 (WT)
Ad-DUSP22 (C88S)
119 kD
p-FAKY576+Y577
119 kD
p-FAKY397
119 kD
FAK
39 kD
p-IκBα
39 kD
IκBα
65 kD
p-NF-κB
65 kD
NF-κB
36 kD
GAPDH
Ad-GFP/PO
Ad-DUSP22 (WT)/PO
Ad-DUSP22 (C88S)/PO
Relative protein levels (fold)
p-FAKY576+Y577
p-FAKY397
p-IκBα
p-NF-κB
e
L02
Ad-GFP/PO
Ad-DUSP22 (WT)/PO
Ad-DUSP22 (C88S)/PO
Relative mRNA levels (fold)
TNF-α
IL-1β
IL-6
IL-18
MCP-1
CXCR2
CXCL2
IL-1α
CX3CR1
CXCL10
CXCL9
CCL5
CXCL3
CCL4
CD206
IL-10
f
Primary hepatocytes
Ad-GFP/PO
Ad-DUSP22 (WT)/PO
Ad-DUSP22 (C88S)/PO
Relative mRNA levels (fold)
SREBP1C
CYP7A1
HMGCR
ABCG1
CD36
FATP1
FABP1
FASN
SCD1
DGAT2
ACACA
PPARγ
PPARα
ACOX1
UCP2
CPT1α
ACSL1
Cholesterol synthesis and efflux
Fatty acid uptake
Fatty acid synthesis
Fatty acid oxidation
g
L02
Ad-GFP/PO
Ad-DUSP22 (WT)/PO
Ad-DUSP22 (C88S)/PO
Relative mRNA levels (fold)
SREBP1C
CYP7A1
HMGCR
ABCG1
CD36
FATP1
FABP1
FASN
SCD1
DGAT2
ACACA
PPARγ
PPARα
ACOX1
UCP2
CPT1α
ACSL1
Cholesterol synthesis and efflux
Fatty acid uptake
Fatty acid synthesis
Fatty acid oxidation
h
LX2
CM from Ad-GFP/PO hepatocytes
CM from Ad-DUSP22 (WT)/PO hepatocytes
CM from Ad-DUSP22 (C88S)/PO hepatocytes
Relative mRNA levels (fold)
α-SMA
COL1A1
COL3A1
CTGF
Fibronectin
TGF-β1(+)

Supplementary Figure 19. DUSP22-C88S is responsible for FAK inactivation *in vitro*. **a-c** Results of (**a**) representative Oil red O staining images, (**b**) intracellular TG contents and (**c**) Nile Red staining images of 24 h BSA- or PO (0.4 mM PA and 0.8 mM OA)-treated L02 cells transfected with Ad-DUSP22 (WT) and Ad-DUSP22 (C88S) (n=4 per group, with 10 images per group; Scale bar, 25 μm) ($^{**}P$<0.01; *ns*, no significant difference). **d** Western blotting and quantification of the protein expression of total and phosphorylated $FAK^{Y576+Y577}$, FAK^{Y397} and NF-κB in L02 cells transfected with Ad-GFP, Ad-DUSP22 (WT) and Ad-DUSP22 (C88S) after PO (0.4 mM PA and 0.8 mM OA) incubation for 24 h (n=3 per group). **e** RT-qPCR analysis of inflammation-related genes as exhibited in L02 cells transfected with Ad-GFP, Ad-DUSP22 (WT) and Ad-DUSP22 (C88S) in response to PO (0.4 mM PA and 0.8 mM OA) for 24 h (n=3 per group) ($^{*}P$<0.05 and $^{**}P$<0.01; *ns*, no significant difference). **f,g** RT-qPCR results for lipid metabolism-associated genes as displayed in (**f**) mouse primary hepatocytes and (**g**) L02 cells transfected with Ad-GFP, Ad-DUSP22 (WT) and Ad-DUSP22 (C88S) after PO (0.4 mM PA and 0.8 mM OA) exposure for 24 h (n=3 per group) ($^{*}P$<0.05 and $^{**}P$<0.01; *ns*, no significant difference). **h** RT-qPCR analysis of fibrosis-related genes as shown in LX2 cells cultured in fresh medium mixed with CM derived from (**f**) at 1:1 ratio for 24 h (n=3 per group) ($^{**}P$<0.01; *ns*, no significant difference). Data are expressed as mean ± SEM from at least three independent experiments. Statistical analysis was conducted by one-way ANOVA.

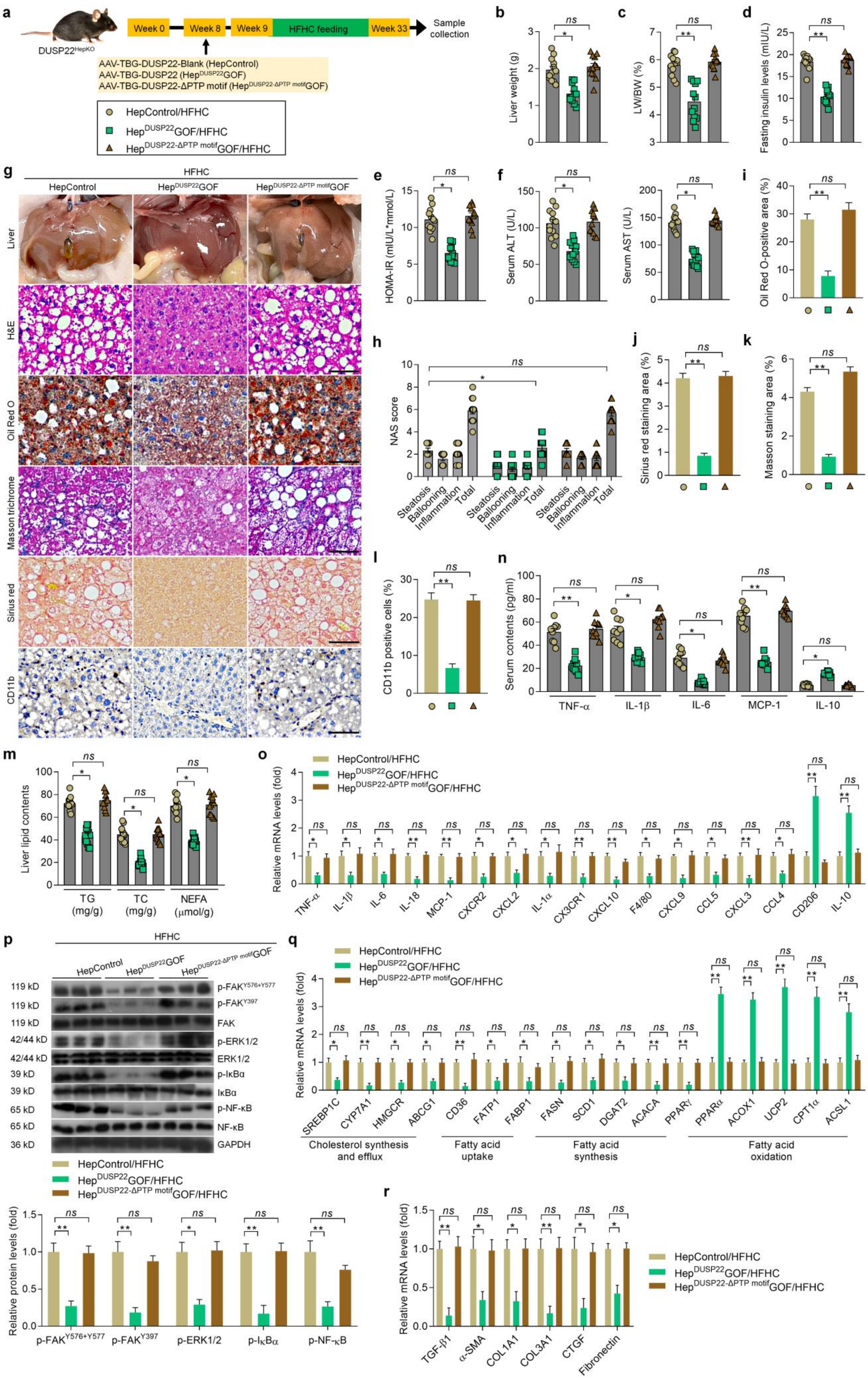

Supplementary Figure 20. DUSP22 (PTP motif) mitigates NASH *in vivo*. a Scheme showing AAV-TBG-DUSP22 and AAV-loaded DUSP22 with PTP-motif domain deletion (AAV-TBG-DUSP22 (ΔPTP motif))-regulated hepatocyte-specific DUSP22 gain-of-function (GOF) in liver of a 24-week HFHC-fed $DUSP22^{HepKO}$ mice (Hep^{DUSP22}GOF/HFHC; $Hep^{DUSP22\text{-}\Delta PTP\ motif}$ GOF/HFHC). The AAV-TBG-blank was used as control (HepControl/HFHC). **b-f** Measurements of (**b**) liver weight, (**c**) ratio of LW/BW, (**d**) fasting insulin levels, (**e**) HOMA-IR index, and (**f**) serum ALT and AST contents in the groups of mice as shown (n=12 mice per group) ($^{*}P<0.05$ and $^{**}P<0.01$; *ns*, no significant difference). **g** Representative images of liver appearance, H&E staining, Oil Red O staining, Masson trichrome staining, Sirius red staining and immunohistochemistry examination of CD11b in liver sections from HepControl, Hep^{DUSP22}GOF and $Hep^{DUSP22\text{-}\Delta PTP\ motif}$ mice fed with a HFHC for 24 weeks (n=6 or 12 mice per group, with 10 images for each mouse; Scale bars, 50 μm). **h-l** Results for (**h**) NAS score, (**i**) Oil Red O-positive staining, (**j**) Sirius red-positive staining, (**k**) Masson trichrome-positive staining, and (**l**) CD11b-positive cells were analyzed and quantified (n=6 or 12 mice per group) ($^{*}P<0.05$ and $^{**}P<0.01$; *ns*, no significant difference). **m** Examination for TG, TC and NEFA contents in liver of the shown groups of mice (n=12 mice per group) ($^{*}P<0.05$; *ns*, no significant difference). **n** Measurements of serum concentrations of inflammatory factors TNF-α, IL-1β, IL-6, MCP-1 and IL-10 from the indicated groups of mice (n=9 mice per group) ($^{*}P<0.05$ and $^{**}P<0.01$; *ns*, no significant difference). **o** RT-qPCR analysis for mRNA levels of inflammation-associated genes in livers of the displayed groups of mice (n=4 mice per group) ($^{*}P<0.05$ and $^{**}P<0.01$; *ns*, no significant difference). **p** Representative western blotting and quantification of the protein expression of total and phosphorylated $FAK^{Y576+Y577}$, FAK^{Y397}, IκBα and NF-κB in liver of the indicated groups of mice fed with a HFHC for 24 weeks (n=4 mice per group) ($^{*}P<0.05$ and $^{**}P<0.01$; *ns*, no significant difference). **q** RT-qPCR analysis of mRNA levels for lipid metabolism-related genes in the liver of the shown groups of mice (n=4 mice per group) ($^{*}P<0.05$ and $^{**}P<0.01$; *ns*, no significant difference). **r** RT-qPCR results for mRNA levels of fibrosis-related genes in livers of the indicated groups of mice (n=4 mice per group) ($^{*}P<0.05$ and $^{**}P<0.01$; *ns*, no significant difference). Data are expressed as mean ± SEM from at least three independent experiments. Statistical analysis was conducted by one-way ANOVA.

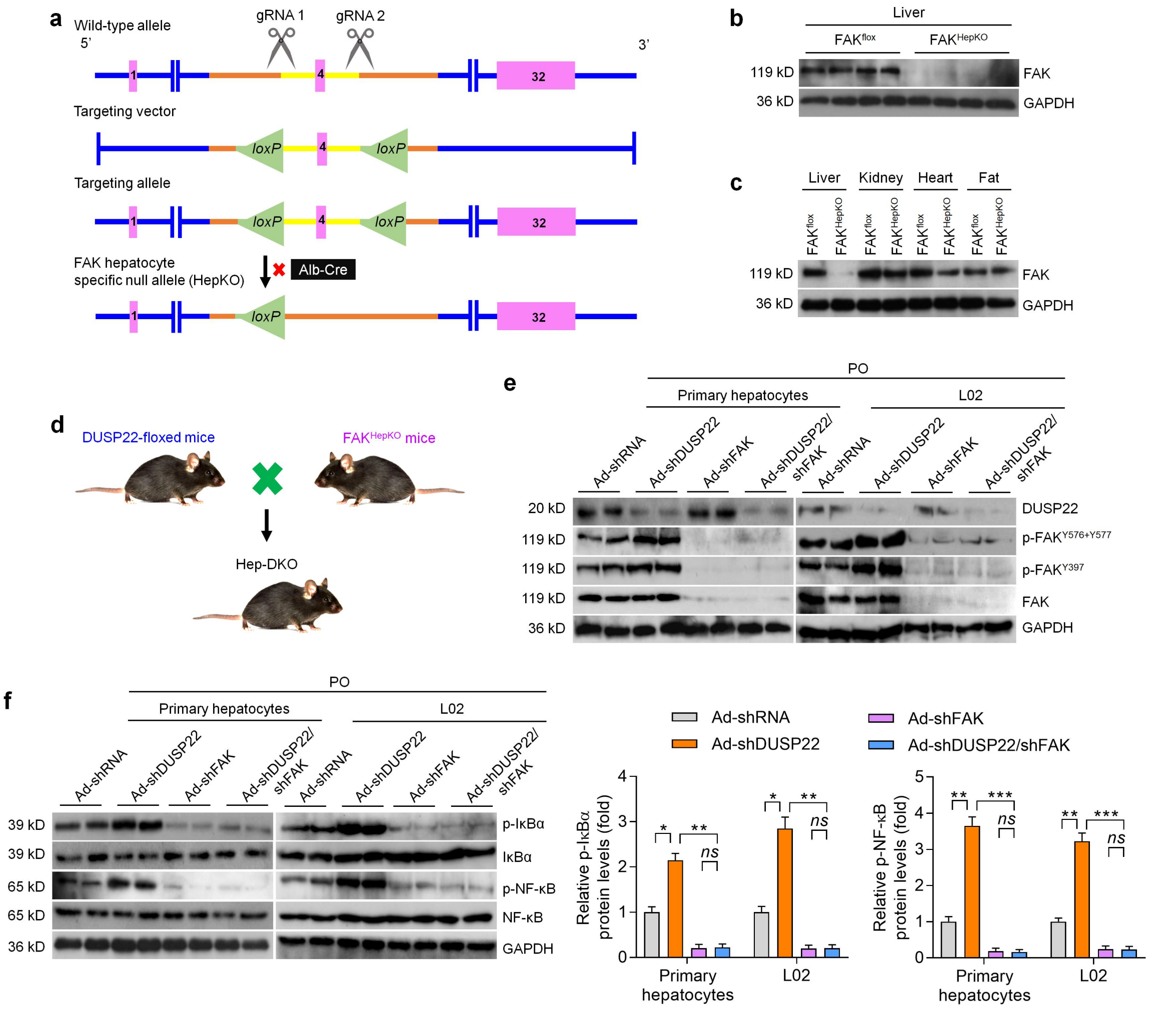

a
Wild-type allele
5'
3'
gRNA 1
gRNA 2
Targeting vector
loxP
Targeting allele
FAK hepatocyte specific null allele (HepKO)
Alb-Cre
b
Liver
FAK^flox
FAK^HepKO
119 kD
36 kD
FAK
GAPDH
c
Liver
Kidney
Heart
Fat
d
DUSP22-floxed mice
FAK^HepKO mice
Hep-DKO
e
PO
Primary hepatocytes
L02
Ad-shRNA
Ad-shDUSP22
Ad-shFAK
Ad-shDUSP22/shFAK
20 kD
DUSP22
p-FAK^Y576+Y577
p-FAK^Y397
f
39 kD
65 kD
p-IκBα
IκBα
p-NF-κB
NF-κB
Relative p-IκBα protein levels (fold)
Relative p-NF-κB protein levels (fold)
ns

Supplementary Figure 21. Construction of hepatocyte-specific FAK knockout mice, and FAK is required for DUSP22 to restrain NF-κB signaling *in vitro*. a Schematic workflow showing the establishment of the hepatocyte-specific FAK-knockout (FAK^{HepKO}) mouse strain. **b** Western blot showing FAK expression in liver from FAK^{flox} and FAK^{HepKO} mice (n=4 mice per group). **c** Western blot showing FAK expression in liver, kidney, heart and fat of the indicated mice (n=3 mice per group). **d** Schematic diagram of the establishment of hepatocyte-specific DUSP22 and FAK double knockout (HepDKO) mouse strain. **e** Representative western blotting for the expression of DUSP22, $p\text{-}FAK^{Y576+Y577}$, $p\text{-}FAK^{Y397}$ and FAK in the 24 h PO (0.4 mM PA and 0.8 mM OA)-treated primary hepatocytes or L02 cells transfected or co-transfected with Ad-shDUSP22, Ad-shFAK and Ad-shDUSP22/shFAK as shown. Cells transfected with Ad-shRNA were used as a control (n=4 per group). **f** Representative western blotting (left) and quantification (wight) for the expression of total and phosphorylated IκBα and NF-κB in the 24 h PO (0.4 mM PA and 0.8 mM OA)-treated primary hepatocytes or L02 cells transfected or co-transfected with Ad-shDUSP22, Ad-shFAK and Ad-shDUSP22/shFAK as shown. Cells transfected with Ad-shRNA were used as a control (n=4 per group) ($^{*}P<0.05$, $^{**}P<0.01$ and $^{***}P<0.001$; *ns*, no significant difference). Data are expressed as mean ± SEM from at least three independent experiments. Statistical analysis was performed by two-tailed Student's *t*-test.

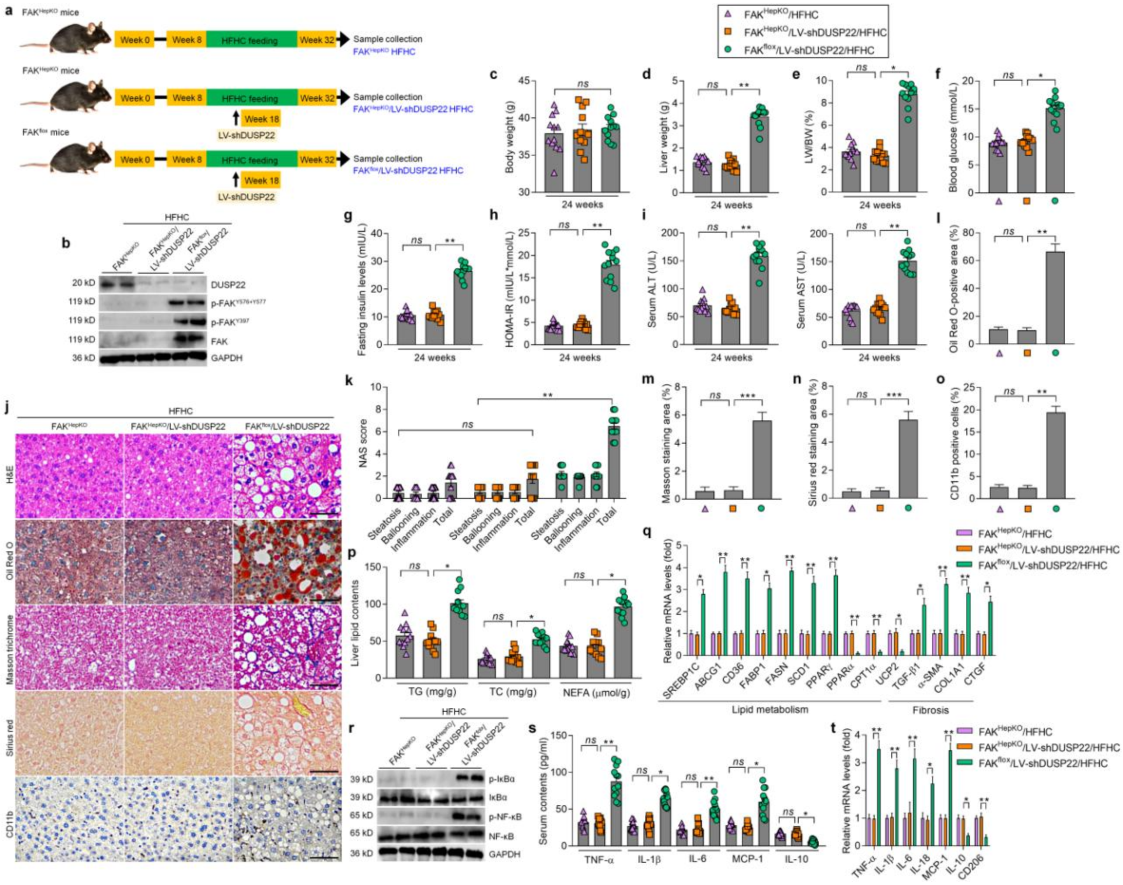

Supplementary Figure 22. FAK is indispensable for the protective function of DUSP22 against HFHC-induced NASH. a Schematic diagram of the establishment of LV-shDUSP22-regulated hepatocyte-specific DUSP22 loss-of function in FAK^{HepKO} mice (FAK^{HepKO}/LV-shDUSP22 HFHC) and FAK^{flox} mice (FAK^{flox}/LV-shDUSP22 HFHC) halfway through a 24-week HFHC feeding treatment. **b** Western blotting showing DUSP22, p-$FAK^{Y576+Y577}$, p-FAK^{Y397} and FAK expression in liver of FAK^{HepKO}, FAK^{HepKO}/LV-shDUSP22 and FAK^{flox}/LV-shDUSP22 mice after HFHC feeding for 24 weeks (n=4 mice per group). **c-i** Measurements of (**c**) body weight, (**d**) liver weight, (**e**) ratio of LW/BW index, (**f**) blood glucose levels, (**g**) fasting insulin levels, (**h**) HOMA-IR index, and (**i**) serum ALT and AST concentrations in mice from the shown groups (n=12 mice per group) ($^{*}P<0.05$ and $^{**}P<0.01$; *ns*, no significant difference). **j** Representative images of H&E staining, Oil Red O staining, Masson trichrome staining, Sirius red staining and immunohistochemistry examination of CD11b in liver sections from the shown groups of mice fed with a HFHC for 24 weeks (n=6 or 12 mice per group, with 10 images for each mouse; Scale bars, 50 μm). **k-o** Results for (**k**) NAS score, (**l**) Oil Red O-positive staining, (**m**) Masson trichrome-positive staining, (**n**) Sirius red-positive staining, and (**o**) CD11b-positive cells were analyzed and quantified (n=6 or 12 mice per group) ($^{*}P<0.05$, $^{**}P<0.01$ and $^{***}P<0.001$; *ns*, no significant difference). **p** Examination of liver TG, TC and NEFA contents from the indicated groups of mice (n=12 mice per group) ($^{*}P<0.05$; *ns*, no significant difference). **q** RT-qPCR results for crucial lipid metabolism- and fibrosis-associated genes as presented in liver of the shown groups of mice (n=4 mice per group) ($^{*}P<0.05$ and $^{**}P<0.01$). **r** Representative western blotting of the protein expression of total and phosphorylated IκBα and NF-κB in liver of the indicated groups of mice (n=4 mice per group). **s** Assessments of serum levels of TNF-α, IL-1β, IL-6, MCP-1 and IL-10 from the shown groups of mice (n=12 mice per group) ($^{*}P<0.05$ and $^{**}P<0.01$; *ns*, no significant difference). **t** RT-qPCR analysis of several critical inflammation-related genes in liver of the presented groups of mice (n=4 mice per group) ($^{*}P<0.05$ and $^{**}P<0.01$). Data are expressed as mean ± SEM from at least three independent experiments. For statistical analysis, **c-p**, and **s** were performed by one-way ANOVA; **q** and **t** were carried out by two-tailed Student's *t*-test.

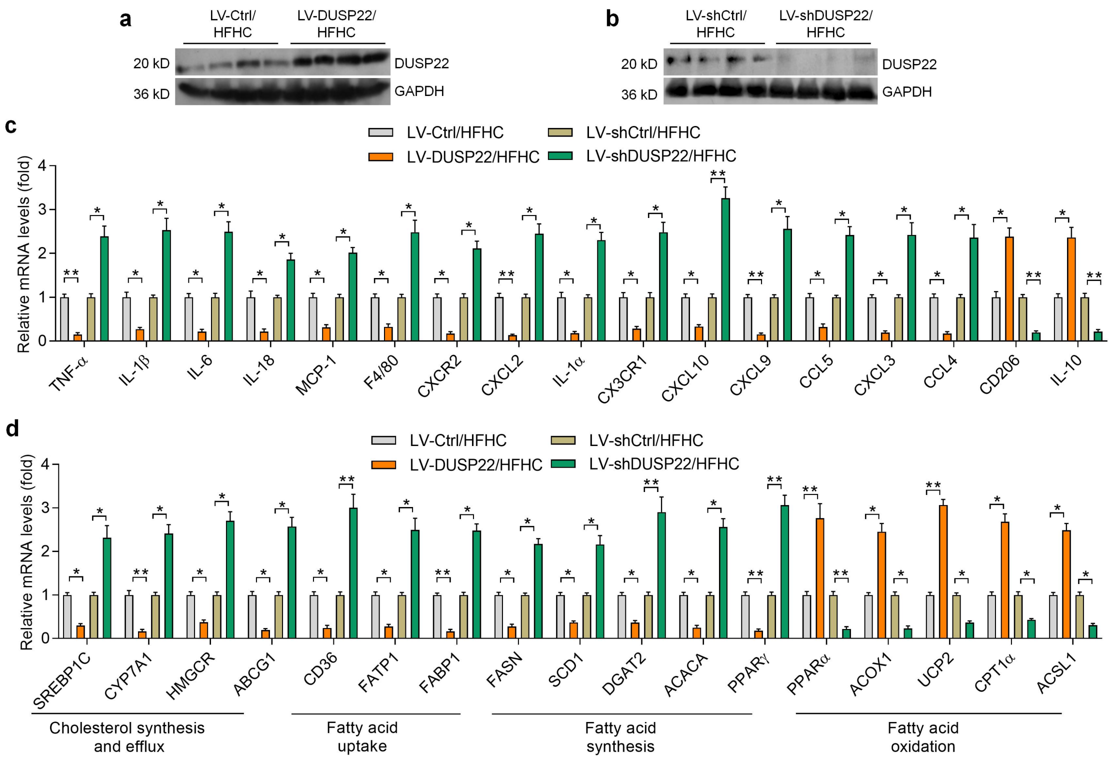
a
LV-Ctrl/HFHC
LV-DUSP22/HFHC
20 kD
36 kD
DUSP22
GAPDH
b
LV-shCtrl/HFHC
LV-shDUSP22/HFHC
20 kD
36 kD
DUSP22
GAPDH
c
LV-Ctrl/HFHC
LV-DUSP22/HFHC
LV-shCtrl/HFHC
LV-shDUSP22/HFHC
Relative mRNA levels (fold)
TNF-α
IL-1β
IL-6
IL-18
MCP-1
F4/80
CXCR2
CXCL2
IL-1α
CX3CR1
CXCL10
CXCL9
CCL5
CXCL3
CCL4
CD206
IL-10
d
LV-Ctrl/HFHC
LV-DUSP22/HFHC
LV-shCtrl/HFHC
LV-shDUSP22/HFHC
Relative mRNA levels (fold)
SREBP1C
CYP7A1
HMGCR
ABCG1
CD36
FATP1
FABP1
FASN
SCD1
DGAT2
ACACA
PPARγ
PPARα
ACOX1
UCP2
CPT1α
ACSL1
Cholesterol synthesis and efflux
Fatty acid uptake
Fatty acid synthesis
Fatty acid oxidation

Supplementary Figure 23. DUSP22 protects against HFHC-induced hepatic dyslipidemia and inflammation. a,b Western blotting bands of DUSP22 protein expression levels in liver of HFHC-fed transplanted (**a**) LV-DUSP22 mice, and (**b**) LV-shDUSP22 mice. LV-Ctrl and LV-shCtrl were used as corresponding controls (n=4 mice per group). **c** RT-qPCR results for inflammation-related molecules in liver of the indicated groups of mice (n=6 mice per group) ($^{*}P$<0.05 and $^{**}P$<0.01). **d** RT-qPCR results of lipid metabolism-associated genes in liver of the shown groups of mice (n=6 mice per group) ($^{*}P$<0.05 and $^{**}P$<0.01). Data are expressed as mean ± SEM from at least three independent experiments. Statistical analysis were carried out by two-tailed Student's *t*-test.

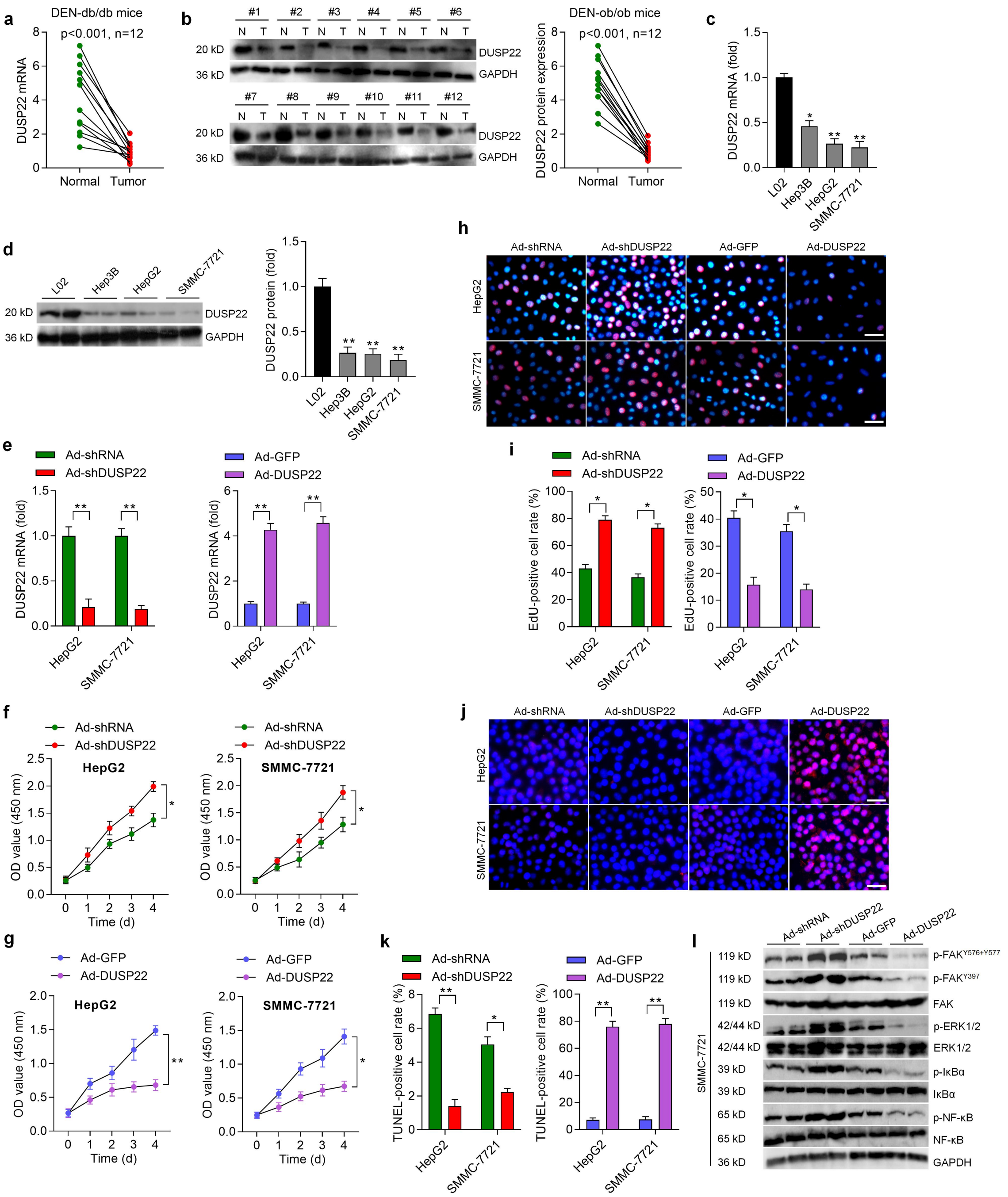

a
DEN-db/db mice
p<0.001, n=12
DUSP22 mRNA
Normal
Tumor
b
DUSP22
GAPDH
20 kD
36 kD
DEN-ob/ob mice
p<0.001, n=12
DUSP22 protein expression
c
DUSP22 mRNA (fold)
L02
Hep3B
HepG2
SMMC-7721
d
DUSP22 protein (fold)
e
Ad-shRNA
Ad-shDUSP22
Ad-GFP
Ad-DUSP22
f
HepG2
SMMC-7721
OD value (450 nm)
Time (d)
g
h
i
EdU-positive cell rate (%)
j
k
TUNEL-positive cell rate (%)
l
p-FAK$^{Y576+Y577}$
p-FAK^{Y397}
FAK
p-ERK1/2
ERK1/2
p-IκBα
IκBα
p-NF-κB
NF-κB
GAPDH

Supplementary Figure 24. DUSP22 meliorates NASH-HCC cell growth. a,b Results for (**a**) RT-qPCR and (**b**) western blotting analysis of DUSP22 mRNA and protein expression levels, respectively, in liver of genetic NASH-HCC mouse models: DEN-treated ob/ob mice (n=12 mice per group). **c,d** Results for (**c**) RT-qPCR and (**d**) western blotting analysis of DUSP22 mRNA and protein expression levels, respectively, in human HCC cell lines (Hep3B, HepG2 and SMMC-7721) and in non-tumor L02 cells. DUSP22 expression in L02 cells was used as a control (n=4 per group) ($^{*}P$<0.05 and $^{**}P$<0.01 versus the expression from L02 group). **e** RT-qPCR results for DUSP22 in HepG2 and SMMC-7721 cells transfected with Ad-shDUSP22 (left) or Ad-DUSP22 (right) for DUSP22 knockdown and over-expression, respectively. Ad-shRNA and Ad-GFP were used as corresponding controls (n=4 per group) ($^{**}P$<0.01). **f,g** CCK-8 analysis of cell growth in the indicated stable cell lines with (**f**) DUSP22 knockdown or (**g**) over-expression (n=4 per group) ($^{*}P$<0.05 and $^{**}P$<0.01). **h,i** Representative images (**h**) and quantification (**i**) of the proliferation of HepG2 and SMMC-7721 cells transfected with Ad-shDUSP22 or Ad-DUSP22 by EdU assay (n=5 per group, with 10 images per group; Scale bar, 80 μm) ($^{*}P$<0.05). **j,k** Representative images (**j**) and quantification (**k**) of the apoptotic cell death of HepG2 and SMMC-7721 cells with DUSP22 knockdown or over-expression by TUNEL staining assay (n=5 per group, with 10 images per group; Scale bar, 80 μm) ($^{*}P$<0.05 and $^{**}P$<0.01). **l** Representative western blotting and quantification of the protein expression of total and phosphorylated $FAK^{Y576+Y577}$, FAK^{Y397}, ERK1/2, IκBα and NF-κB in SMMC-7721 cells transfected with Ad-shDUSP22 or Ad-DUSP22 as shown (n=4 per group). Data are expressed as mean ± SEM from at least three independent experiments. Statistical analysis were carried out by two-tailed Student's *t*-test.

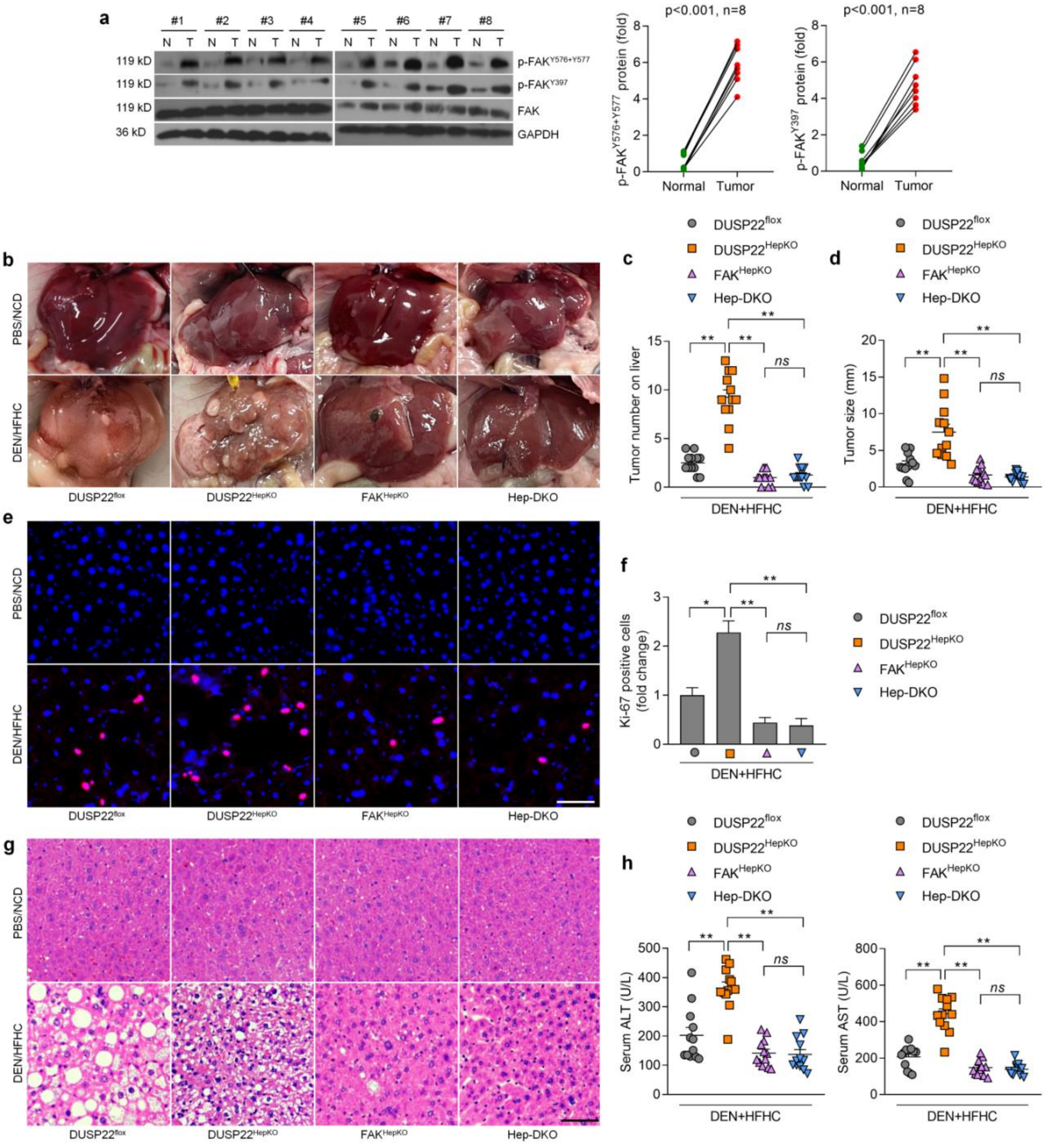

a
#1 #2 #3 #4 #5 #6 #7 #8
N T N T N T N T N T N T N T N T
119 kD p-FAKY576+Y577
119 kD p-FAKY397
119 kD FAK
36 kD GAPDH
p<0.001, n=8
p-FAKY576+Y577 protein (fold)
Normal Tumor
p<0.001, n=8
p-FAKY397 protein (fold)
Normal Tumor
b
PBS/NCD
DEN/HFHC
DUSP22flox DUSP22HepKO FAKHepKO Hep-DKO
c
DUSP22flox
DUSP22HepKO
FAKHepKO
Hep-DKO
Tumor number on liver
DEN+HFHC
d
Tumor size (mm)
DEN+HFHC
e
PBS/NCD
DEN/HFHC
DUSP22flox DUSP22HepKO FAKHepKO Hep-DKO
f
Ki-67 positive cells (fold change)
DEN+HFHC
g
PBS/NCD
DEN/HFHC
DUSP22flox DUSP22HepKO FAKHepKO Hep-DKO
h
Serum ALT (U/L)
DEN+HFHC
Serum AST (U/L)
DEN+HFHC

Supplementary Figure 25. FAK blockage is involved in DUSP22-inhibited progression of NASH-HCC induced by DEN/HFHC. a Western blotting analysis for p-FAK$^{Y576+Y577}$ and p-FAK^{Y397} protein expression levels in individual paired NAFLD-HCC (T) and adjacent normal samples (N) (n=8). **b** Representative images of liver appearances (n=12 mice per group). **c,d** Quantification for (**c**) surface tumor number and (**d**) tumor size in each shown group (n=12 per group) ($^{**}P<0.01$; *ns*, no significant difference). **e** Immunofluorescence examination of Ki-67 (Scale bars, 20 μm) in liver sections from the shown groups of mice (n=6 mice per group, with 10 images for each mouse). **f** Number of Ki-67-positive cells was quantified ($^{*}P<0.05$ and $^{**}P<0.01$; *ns*, no significant difference). **g** Representative images of H&E staining (Scale bars, 50 μm) in liver sections from the shown groups of mice (n=6 mice per group, with 10 images for each mouse). **h** Serum ALT and AST contents from the indicated groups of mice were examined (n=12 per group) ($^{**}P<0.01$; *ns*, no significant difference). Data are expressed as mean ± SEM from at least three independent experiments. For statistical analysis, **a** was performed by two-tailed Student's *t*-test; **c**, **d**, **e** and **h** were performed by one-way ANOVA.

Supplementary Tables

Supplementary Table 1. Clinical information of non-steatosis individuals and NASH patients.

Items	Non-steatosis	NASH
All number (n)	16	20
Male (n)	11	11
Female (n)	5	9
Age (years)	35.06 ± 2.00	38.05 ± 2.44
BMI (kg/m^2)	19.56 ± 0.62	29.78 ± 0.61
AST (U/L)	24.79 ± 1.88	54.27 ± 2.21
ALT (U/L)	22.22 ± 2.12	42.28 ± 1.74
TG (mmol/L)	1.71 ± 0.07	2.21 ± 0.09
TC (mmol/L)	3.76 ± 0.12	4.46 ± 0.17
Fasting blood glucose (mmol/L)	5.04 ± 0.19	6.28 ± 0.15
Liver IL-6 mRNA (fold)	1.55 ± 0.06	3.84 ± 0.20
Liver DUSP22 protein (fold)	1.00 ± 0.05	0.34 ± 0.04
Liver TNF-α mRNA (fold)	1.40 ± 0.08	4.61 ± 0.09
LN (μg/L)	78.31 ± 7.16	158.32 ± 8.32
HA (μg/L)	37.00 ± 2.81	101.47 ± 5.50
IVC (μg/L)	70.99 ± 6.44	113.05 ± 5.44
PCIII (μg/L)	49.31 ± 5.73	116.64 ± 8.41
GGT (U/L)	28.96 ± 2.71	71.83 ± 3.79
AKP (U/L)	86.74 ± 4.83	780.73 ± 7.6

Data are expressed as mean ± SEM unless otherwise stated. BMI, body mass index; AST, aspartate aminotransferase; ALT, alanine aminotransferase; TG, triglycerides; TC, total cholesterol; LN, laminin; HA, hyaluronuc acid; IVC, collagen type IV; PCIII, type III precollagen; GGT, γ-glutamyl Transpeptidase; AKP, alkline phosphatase.

Supplementary Table 2. Detailed information for non-steatosis individuals and NASH patients.

Items	Sex	Age (Years)	BMI (Kg/m^2)	AST (U/L)	ALT (U/L)	TG (mmol/L)	TC (mmol/L)	FBG (mmol/L)	NAS
Non-steatosis	Female	35	19.5	30.3	11.8	1.41	3.23	4.2	0
Non-steatosis	Male	43	15.2	32.1	18.7	1.33	4.71	5.7	0
Non-steatosis	Male	29	19.9	29.5	24.3	2.18	3.7	5.8	0
Non-steatosis	Male	26	19.9	27.0	25.7	1.35	3.42	4.7	0
Non-steatosis	Male	33	19.2	15.5	19.1	2.01	3.66	5.5	0
Non-steatosis	Male	21	17.9	30.7	27.5	1.47	3.73	4.1	0
Non-steatosis	Female	32	18.3	15.0	13.2	1.92	3.22	5.6	0
Non-steatosis	Female	29	18.1	15.2	30.9	1.93	3.25	4.0	0
Non-steatosis	Male	28	20.2	30.8	30.9	1.75	3.56	4.7	0
Non-steatosis	Male	30	19.9	33.8	13.6	1.42	3.91	5.3	0
Non-steatosis	Female	35	15.6	13.7	26.1	1.56	3.38	5.8	0
Non-steatosis	Male	41	18.1	31.8	29.8	1.88	4.08	4.3	0
Non-steatosis	Male	39	23.9	31.3	36.4	1.81	4.15	4.3	0
Non-steatosis	Female	47	23.9	21.4	22.4	1.63	3.47	6.0	0
Non-steatosis	Male	46	22.1	23.0	10.4	2.20	3.96	4.6	0
Non-steatosis	Male	47	21.6	15.6	10.7	1.60	4.78	6.1	0
NASH	Male	33	31.3	37.8	36.3	2.25	3.84	7.1	4
NASH	Female	31	30.3	64.4	57.2	3.54	5.96	7.9	4
NASH	Male	26	28.1	48.0	43.7	1.98	3.64	5.0	5
NASH	Female	32	37.4	59.7	39.9	2.45	4.13	5.2	4
NASH	Female	29	30.9	40.3	32.8	1.67	3.12	5.7	4
NASH	Male	30	28.3	46.6	48.1	2.67	3.71	5.3	5
NASH	Male	45	28.4	59.5	33.6	1.96	4.68	5.0	3
NASH	Male	28	31.5	54.8	37.0	2.33	4.52	6.7	4
NASH	Female	22	30.5	62.0	49.1	2.14	3.16	6.3	6
NASH	Female	35	28.1	62.0	54.5	2.58	4.02	5.9	5
NASH	Male	29	31.3	60.1	31.8	1.98	5.21	6.2	4
NASH	Male	27	29.4	56.9	37.8	1.91	5.9	6.7	4
NASH	Male	45	24.4	42.0	53.7	2.05	4.2	6.6	5
NASH	Female	55	27.3	55.8	33.6	2.11	5.11	6.9	4
NASH	Female	54	31.3	36.6	49.2	2.32	4.89	6.6	4
NASH	Female	54	32.5	44.4	34.7	2.41	4.75	6.3	4
NASH	Male	54	31.2	66.3	42.1	2.14	4.30	6.6	8
NASH	Male	39	28.1	59.2	46.3	2.02	4.71	5.9	6
NASH	Male	47	29.4	68.2	39.8	1.80	4.25	6.3	8
NASH	Female	46	26.3	60.8	44.4	1.97	5.06	6.4	5

FBG, fasting blood glucose; NAS, NAFLD activity score.

Supplementary Table 3. Clinical pathological information of the NAFLD-associated HCC patients.

Patient no.	Gender	Age (years)	Hep B	Hep C	Fatty liver	Diabetes	Hypertension	Dyslipidemia
1	Male	56	Negative	Negative	Yes	Yes	Yes	No
2	Male	69	Negative	Negative	Yes	Yes	Yes	Yes
3	Male	38	Negative	Negative	Yes	No	Yes	Yes
4	Female	72	Negative	Negative	Yes	Yes	No	Yes
5	Female	78	Negative	Negative	No	Yes	Yes	No
6	Male	74	Negative	Negative	Yes	Yes	Yes	No
7	Male	55	Negative	Negative	No	No	Yes	No
8	Male	62	Negative	Negative	No	Yes	Yes	Yes

Hep B, hepatitis B; Hep C, hepatitis C.

Supplementary Table 4. Primer sequences used for RT-qPCR analysis.

Items	Primer sequences (5'→3')
DUSP22 (Homo sapiens)	Forward: AGCAGCGGATTCACCATCTC
	Reverse: TGATGTATGCGATCACCAGTGT
IL-6 (Homo sapiens)	Forward: CCAGGAGCCCAGCTATGAAC
	Reverse: CCCAGGGAGAAGGCAACTG
TNF-α (Homo sapiens)	Forward: CCTCTCTCTAATCAGCCCTCTG
	Reverse: GAGGACCTGGGAGTAGATGAG
IL-1β (Homo sapiens)	Forward: CCTGCGTGTTGAAAGATGATAA
	Reverse: CTGCTTGAGAGGTGCTGATGTA
IL-18 (Homo sapiens)	Forward: AGAGCGCAATGGTG
	Reverse: GACGCATGCCCTCAA
MCP-1 (Homo sapiens)	Forward: GTCTCTGCCGCCCTTCTG
	Reverse: ACTTGCTGCTGGTGATTCTTCT
CXCR2 (Homo sapiens)	Forward: CATGGAGAGTGACAGCTTTGAA
	Reverse: CTTGTTGATTTCCAGGGATTCTG
CXCL2 (Homo sapiens)	Forward: ATGTCTTTCTTGTAAGGCATACTG
	Reverse: CGAAACCTCTCTGCTCTAACAC
IL-1α (Homo sapiens)	Forward: AATGACGCCCTCAATCAAAG
	Reverse: TGGGTATCTCAGGCATCTCC
CX3CR1 (Homo sapiens)	Forward: CTTACGATGGCACCCAGTGA
	Reverse: CAAGGCAGTCCAGGAGAGTT
CXCL10 (Homo sapiens)	Forward: GTGGCATTCAAGGAGTACCTC
	Reverse: TGATGGCCTTCGATTCTGGATT
CXCL9 (Homo sapiens)	Forward: CCAATACAGGAGTGACTTGGAAC
	Reverse: TCACTACTGGGTTCCTTGC
CCL5 (Homo sapiens)	Forward: CAGCAGTCGTCTTTGTCACC
	Reverse: AGGACTCTCCATCCTAGCTCA
CXCL3 (Homo sapiens)	Forward: GCGTATCATTGACACTTCCTGC
	Reverse: CCTTTCCAGCTGTCCCTAGAA
CCL4 (Homo sapiens)	Forward: AAGTCTGTGCTGATCCCAGT
	Reverse: CAGGTGACCTTCCCTGAAGA
CD206 (Homo sapiens)	Forward: GCCCGGAGTCAGATCACACA
	Reverse: AGTGGCTCAACCCGATATGACAG
IL-10 (Homo sapiens)	Forward: AGGGCACCCAGTCTGAGAACA
	Reverse: CGGCCTTGCTCTTGTTTTCAC
SREBP1C (Homo sapiens)	Forward: CATGTCTTCGATGTCGGTCAG
	Reverse: TCCTGTTGCCCATATGAAATCA
CYP7A1 (Homo sapiens)	Forward: GACATCATAGCTC TTTACCCAC
	Reverse: GGTGTTCTGCAGTCCTGTAAT
HMGCR (Homo sapiens)	Forward: TACCATGTCAGGGGTACGTC
	Reverse: CAAGCCTAGAGACATAATCATC

ABCG1 (Homo sapiens)	Forward: CCCTGTGGAATGTACCTATGTG
	Reverse: GAGGTGTCCCAAAGATGCAA
CD36 (Homo sapiens)	Forward: AGATGCAGCCTCATTTCCAC
	Reverse: GCCTTGGATGGAAGAACAAA
FATP1 (Homo sapiens)	Forward: CCACTTGGATGTCACCACTG
	Reverse: GTGGGACCCTCCAGTAGACA
FABP1 (Homo sapiens)	Forward: ATGAGTTTCTCCGGCAAGTACC
	Reverse: CTCTTCCGGCAGACCGATTG
FASN (Homo sapiens)	Forward: GGACCTGACCTGCCGTCTAG
	Reverse: GAGGAGTGGGTGTCGCTGTT
SCD1 (Homo sapiens)	Forward: CTCCACTGCTGGACATGAGA
	Reverse: AATGAGTGAAGGGGCACAAC
DGAT2 (Homo sapiens)	Forward: ATTGCTGGCTCATCGCTGT
	Reverse: GGGAAAGTAGTCTCGAAAGTAGC
ACACA (Homo sapiens)	Forward: TCCAGGCCAGTACGGAATTC
	Reverse: ACTTTCCTCGCCAAACCAGTAG
PPARγ (Homo sapiens)	Forward: ATGACAGCGACTTGGCAATA
	Reverse: GCAACTGGAAGAAGGGAAAT
PPARα (Homo sapiens)	Forward: GAGATTTCGCAATCCATCGG
	Reverse: CTACATTCGATGTTCAATGC
ACOX1 (Homo sapiens)	Forward: GTCTCCGTCATGAATCCCGA
	Reverse: TGCGATGCCAAATTCCCTCA
UCP2 (Homo sapiens)	Forward: TTGGGTTCAAGGCCACAGAT
	Reverse: CCAGCCCCAAGAAACTTCAC
CPT1α (Homo sapiens)	Forward: TCTTGCAGTCGACTCACCTT
	Reverse: TCCACAGGACACATAGTCAGG
ACSL1 (Homo sapiens)	Forward: ATCTGCAAGCCAGGAAGAGTC
	Reverse: CTTGCTTGATGCTTTGGTCTGT
α-SMA (Homo sapiens)	Forward: GCTACTCCTTCGTGACCACAG
	Reverse: GCCGTCGCCATCTCGTTCT
COL1A1 (Homo sapiens)	Forward: GTGCTCCTGGTATTGCTGGT
	Reverse: TCCTTGAACACCAACAGGGC
COL3A1 (Homo sapiens)	Forward: TTCGACTTCTCTCCAGCCGA
	Reverse: TCCACTGGCCTGATCCATGT
CTGF (Homo sapiens)	Forward: AGACCTGTGCCTGCCATTAC
	Reverse: ACGCCATGTCTCCGTACATC
GAPDH (Homo sapiens)	Forward: GGAGTCAACGGATTTGGTC
	Reverse: GGCAACAATATCCACTTTACC
DUSP22 (Mus musculus)	Forward: GTTGAGCAGGAACAAGGT
	Reverse: TTGAGATGGTGTGTCTGC
IL-6 (Mus musculus)	Forward: CAACGATGATGCACTTGCAGA
	Reverse: TCTCTCTGAAGGACTCTGGCT
TNF-α (Mus musculus)	Forward: ACCTGGCCTCTCTACCTTGT

	Reverse: CCCGTAGGGCGATTACAGTC
IL-1β (Mus musculus)	Forward: GCCACCTTTTGACAGTGATGAG
	Reverse: AGTGATACTGCCTGCCTGAAG
IL-18 (Mus musculus)	Forward: CAAGGCTGGTCCATGCTCC
	Reverse: TGCTATCACTTCCTTTCTGTTGC
MCP-1 (Mus musculus)	Forward: CTGTCACACTGGTCACTCCTAC
	Reverse: CTGTCACACTGGTCACTCCTAC
F4/80 (Mus musculus)	Forward: CGTCAGGTACGGGATGAATATAAG
	Reverse: CTATGCCATCCACTTCCAAGAT
CXCR2 (Mus musculus)	Forward: ATGCCCTCTATTCTGCCAGAT
	Reverse: GTGCTCCGGTTGTATAAGATGAC
CXCL2 (Mus musculus)	Forward: CGGTCAAAAAGTTTGCCTTG
	Reverse: TCCAGGTCAGTTAGCCTTGC
IL-1α (Mus musculus)	Forward: CGAAGACTACAGTTCTGCCATT
	Reverse: GACGTTTCAGAGGTTCTCAGAG
CX3CR1 (Mus musculus)	Forward: ATCCAGTTCAGGGAAGGAGG
	Reverse: AGACTGGGTGAGTGACTGGC
CXCL10 (Mus musculus)	Forward: ATGACGGGCCAGTGAGAATG
	Reverse: ATGATCTCAACACGTGGGCA
CXCL9 (Mus musculus)	Forward: TCTGCATCAGTGACGGTAAAC
	Reverse: TGAAGGGCACAGTTTGGAG
CCL5 (Mus musculus)	Forward: TGCTGCTTTGCCTACCTCTC
	Reverse: TCTTCTCTGGGTTGGCACAC
CXCL3 (Mus musculus)	Forward: CAGCCACACTCCAGCCTA
	Reverse: CACAACAGCCCCTGTAGC
CCL4 (Mus musculus)	Forward: TTCTGTGCTCCAGGGTTCTC
	Reverse: CGGGAGGTGTAAGAGAAACAG
CD206 (Mus musculus)	Forward: GGAATCAAGGGCACAGAGTTA
	Reverse: ATTGTGGAGCAGATGGAA
IL-10 (Mus musculus)	Forward: TTTTCACAGGGGAGAAATCG
	Reverse: CCAAGCCTTATCGGAAATGA
SREBP1C (Mus musculus)	Forward: CACTTCTGGAGACATCGCAAAC
	Reverse: ATGGTAGACAACAGCCGCATC
CYP7A1 (Mus musculus)	Forward: CTGGGCTGTGCTCTGAAGTT
	Reverse: TGCCTTCGCAGAAGTAGTGT
HMGCR (Mus musculus)	Forward: ATCATGTGCTGCTTCGGCTGCAT
	Reverse: AAATTGGACGACCCTCACGGCT
ABCG1 (Mus musculus)	Forward: ATCTACGGCTTGGACCGAGA
	Reverse: TGCCCAGGACGATGAAATCC
CD36 (Mus musculus)	Forward: AAATAAACCTCCTTGGCCTGA
	Reverse: GCAACAAACATCACCACACC
FATP1 (Mus musculus)	Forward: TGCACAGCAGGTACTACCGCAT
	Reverse: TGCGCAGTACCACCGTCAAC

FABP1 (Mus musculus)	Forward: GCAGAGCCAGGAGAACTTTG
	Reverse: GGGTCCATAGGTGATGGTGAG
FASN (Mus musculus)	Forward: CTGCGGAAACTTCAGGAAATG
	Reverse: GGTTCGGAATGCTATCCAGG
SCD1 (Mus musculus)	Forward: CCTACGACAAGAACATTCAATCCC
	Reverse: ACTCACTGGCAGAGTAGTCGAA
DGAT2 (Mus musculus)	Forward: GGCGCTACTTCCGAGACTAC
	Reverse: TGGTCAGCAGGTTGTGTGTC
ACACA (Mus musculus)	Forward: GGCCAGTGCTATGCTGAGAT
	Reverse: AGGGTCAAGTGCTGCTCCA
PPARγ (Mus musculus)	Forward: ATTCTGGCCCACCAACTTCGG
	Reverse: TGGAAGCCTGATGCTTTATCCCCA
PPARα (Mus musculus)	Forward: CAAGGCCTCAGGGTACCACT
	Reverse: TTGCAGCTCCGATCACACTT
ACOX1 (Mus musculus)	Forward: GTCTCCGTCATGAATCCCGA
	Reverse: TGCGATGCCAAATTCCCTCA
UCP2 (Mus musculus)	Forward: ACAAGACCATTGCACGAGAG
	Reverse: CATGGTAAGGGCACAGTGAC
CPT1α (Mus musculus)	Forward: CTCAGTGGGAGCGACTCTTCA
	Reverse: GGCCTCTGTGGTACACGACAA
ACSL1 (Mus musculus)	Forward: TGCCAGAGCTGATTGACATTC
	Reverse: GGCATACCAGAAGGTGGTGAG
α-SMA (Mus musculus)	Forward: TTCCTTCGTGACTACTGCCG
	Reverse: TATAGGTGGTTTCGTGGATGCC
COL1A1 (Mus musculus)	Forward: CGATGGATTCCCGTTCGAGT
	Reverse: AAGGGTGCTGTAGGTGAAGC
COL3A1 (Mus musculus)	Forward: CCCAACCCAGAGATCCCATT
	Reverse: GAAGCACAGGAGCAGGTGTAGA
CTGF (Mus musculus)	Forward: GAGTGTGCACTGCCAAAGAT
	Reverse: GGCAAGTGCATTGGTATTTG
Fibronectin (Mus musculus)	Forward: CACCCGTGAAGAATGAAG
	Reverse: CAGGCAGGAGATTTGTTAG
TGF-β1 (Mus musculus)	Forward: ATTTGGAGCCTGGACACACA
	Reverse: GAGCGCACAATCATGTTGGA
DUSP3 (Mus musculus)	Forward: GGGTGATGCCCAGTTTCT
	Reverse: GATCTCAACGACCTGCTCTC
DUSP8 (Mus musculus)	Forward: TGACCCAAAACGGAATAAGC
	Reverse: AGAGATGCCAGCCAGACAGT
DUSP9 (Mus musculus)	Forward: GGCATCCGCTATATCCTCAA
	Reverse: GGGGATCTGCTTGTAGTGGA
DUSP12 (Mus musculus)	Forward: TCTCTTGTGAGCAAAGGCTATT
	Reverse: CTTCGCTGTTGACCCAACTA
DUSP14 (Mus musculus)	Forward: GAGAAGGAGTCCCGACA

	Reverse: CAGGCAACGCACGTATTTC
DUSP16 (Mus musculus)	Forward: CACTCAGATATTCTGGCTCCC
	Reverse: CTAGACATGGTAGTGGTGATGGC
DUSP26 (Mus musculus)	Forward: ATGCCCTCTGTTCACCATCC
	Reverse: CTGTTGTGTGAGGCGTTGAG
GAPDH (Mus musculus)	Forward: GGTGAAGGTCGGTGTGAACG
	Reverse: CCCGTAGGGCGATTACAGTC

Uncropped western blotting bands in the study

Western blots for figure 1

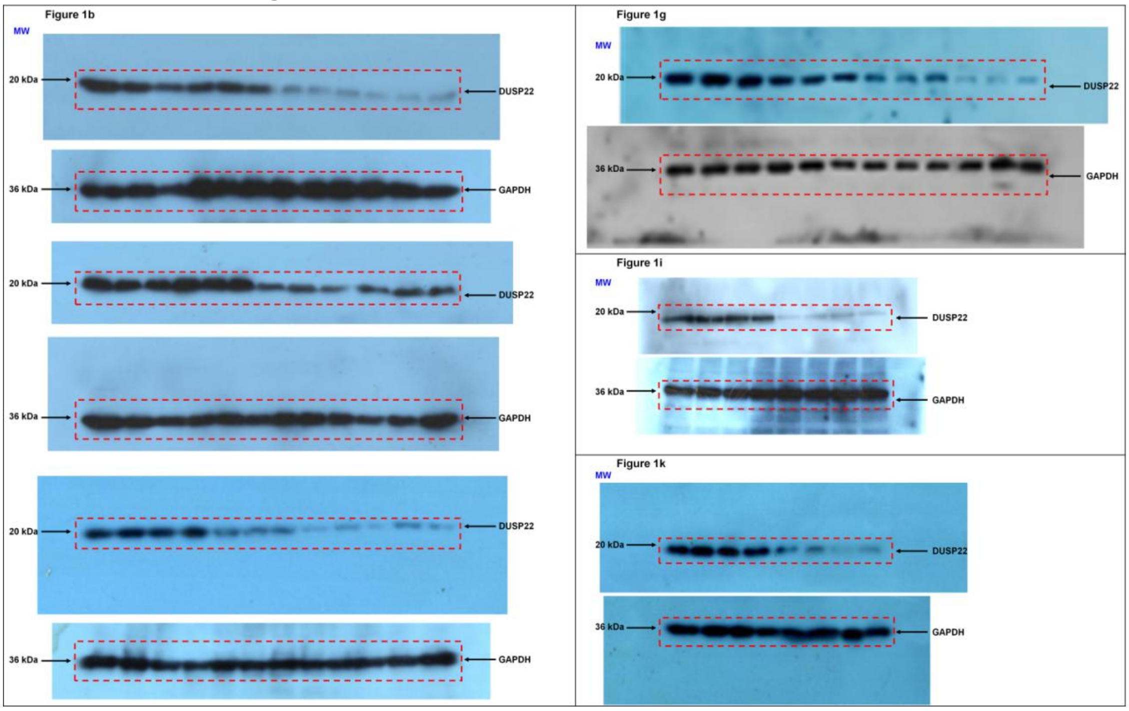

Western blots for figure 4

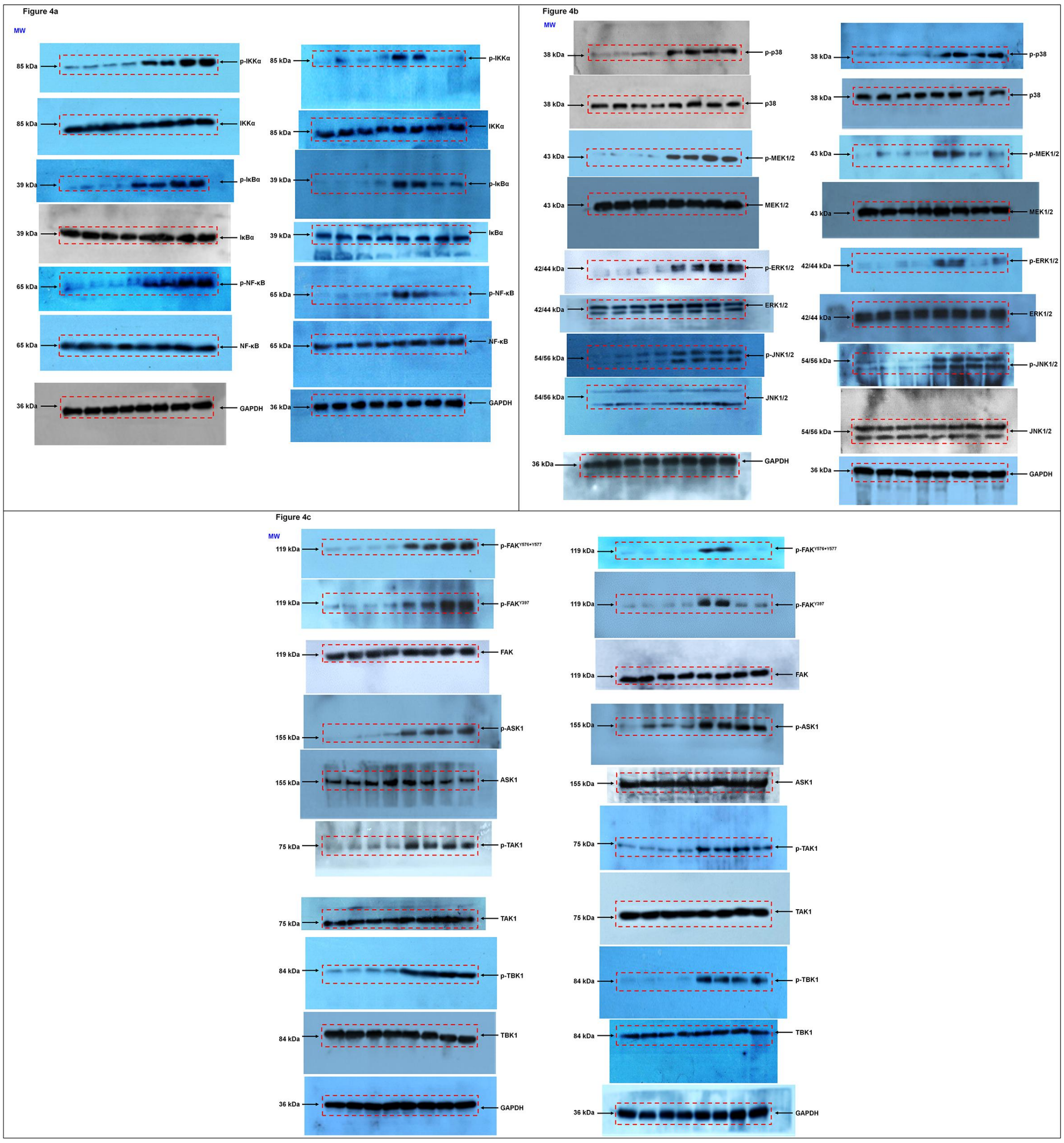

Western blots for figure 5

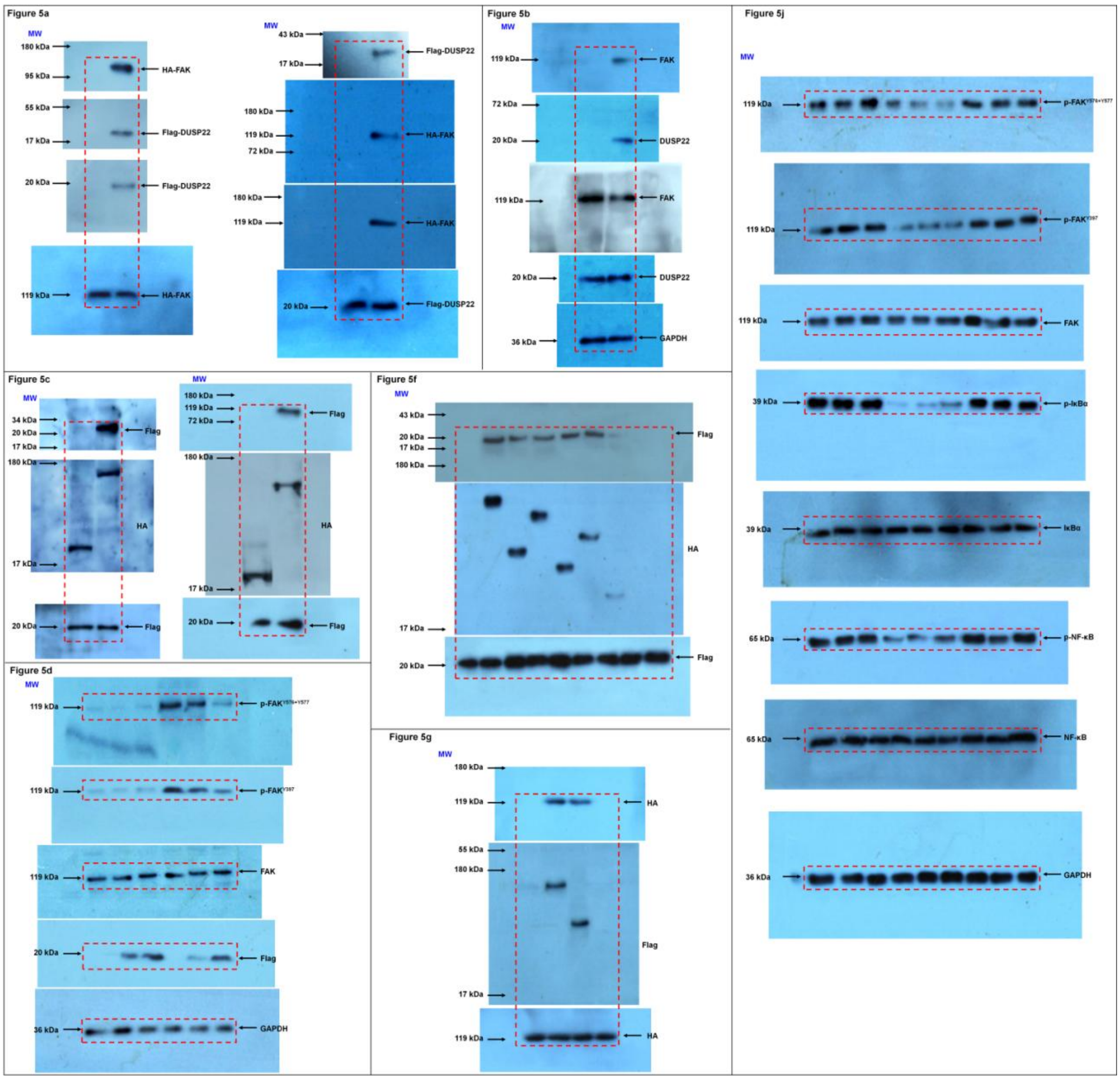

Western blots for figure 6

Figure 6a

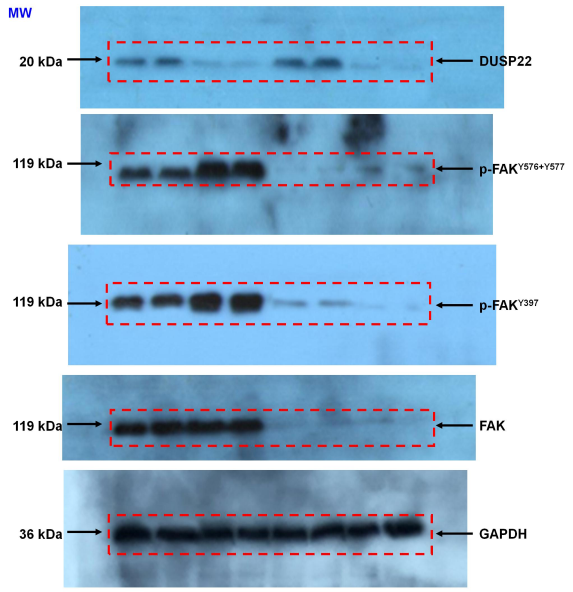

Figure 6p

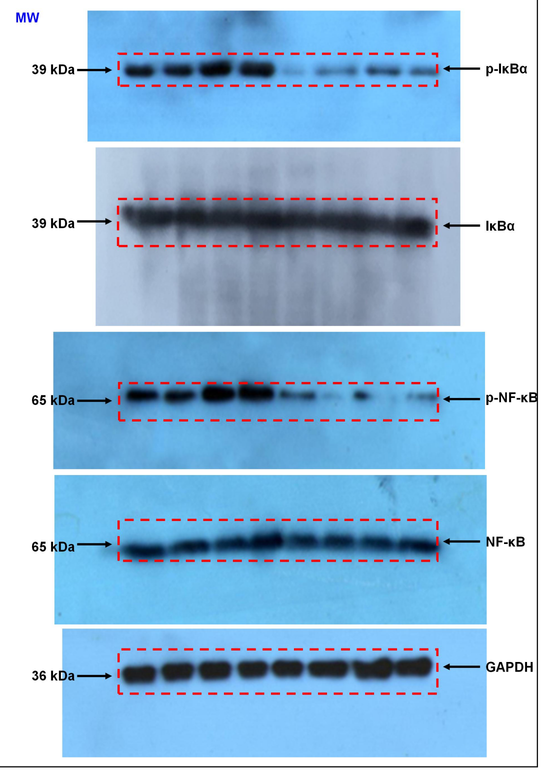

Western blots for figure 7

Figure 7q

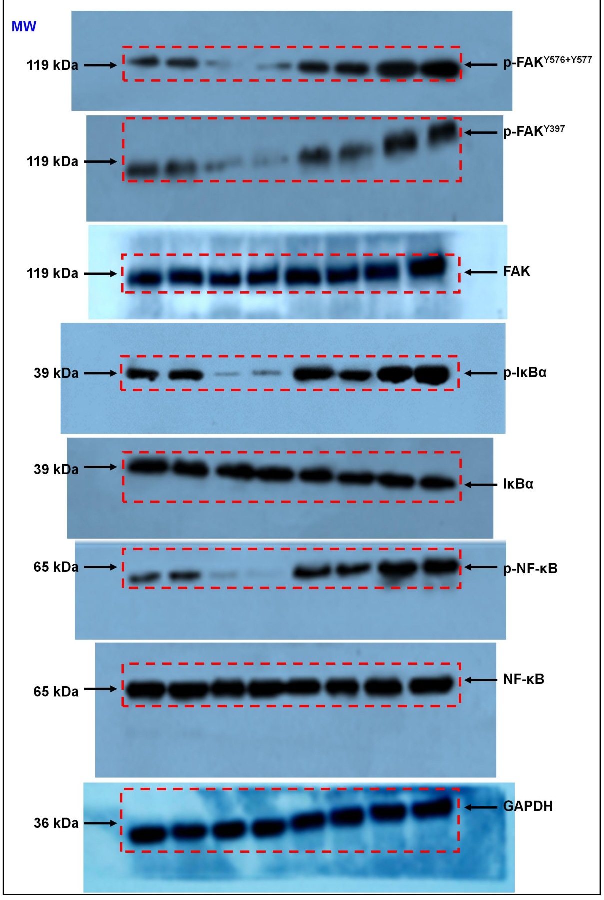

Western blots for figure 8

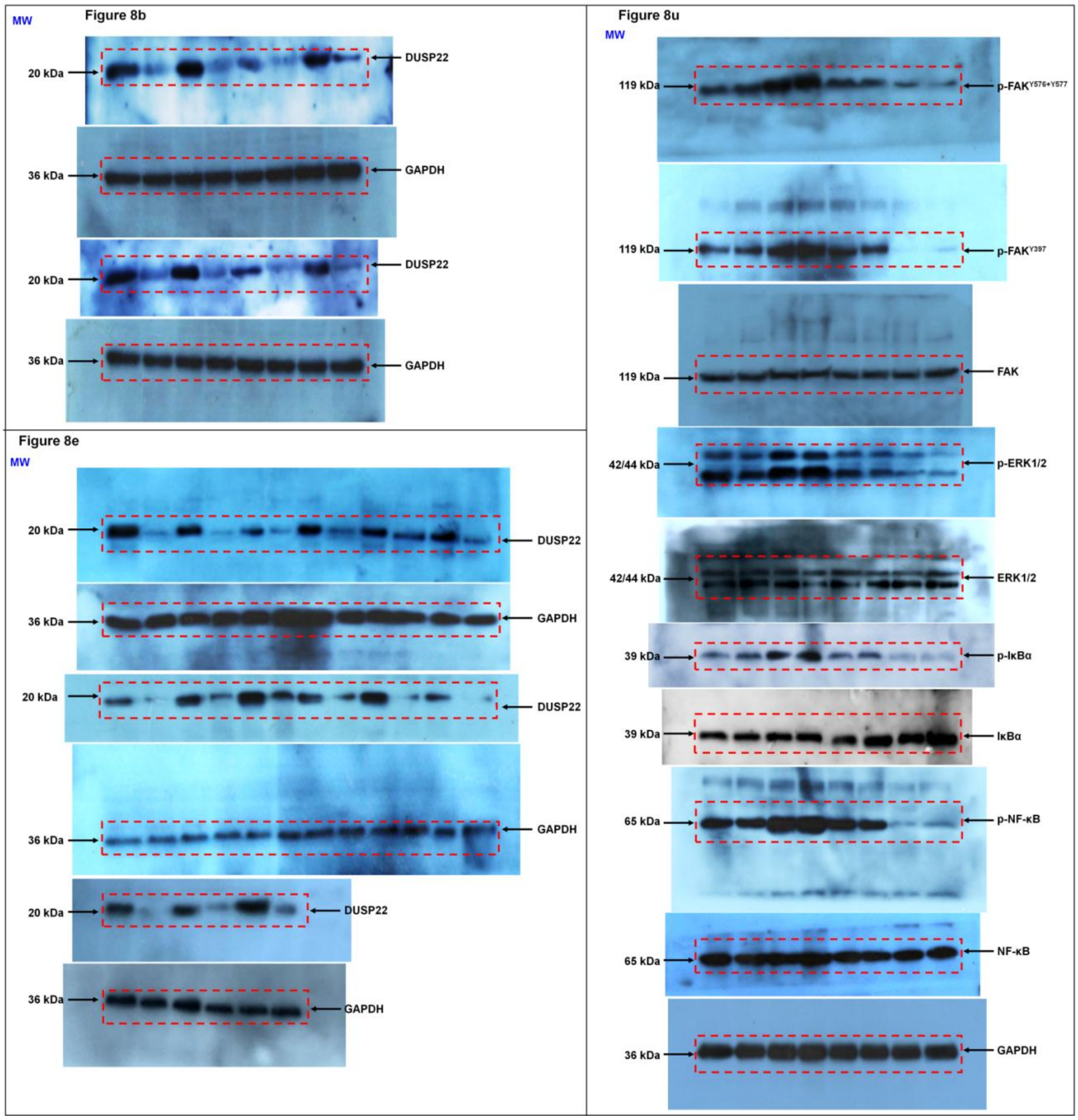

Western blots for supplementary figure 1

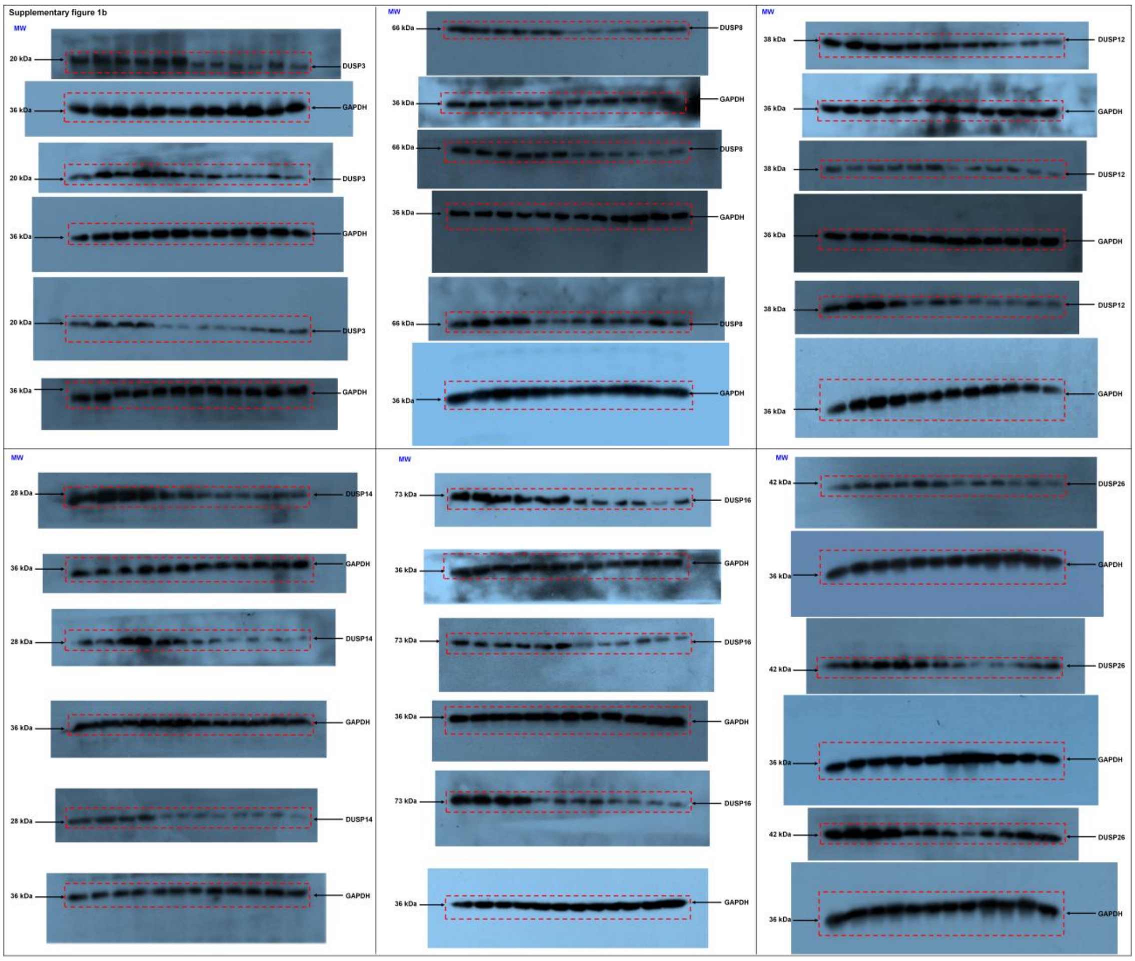

Western blots for supplementary figure 4

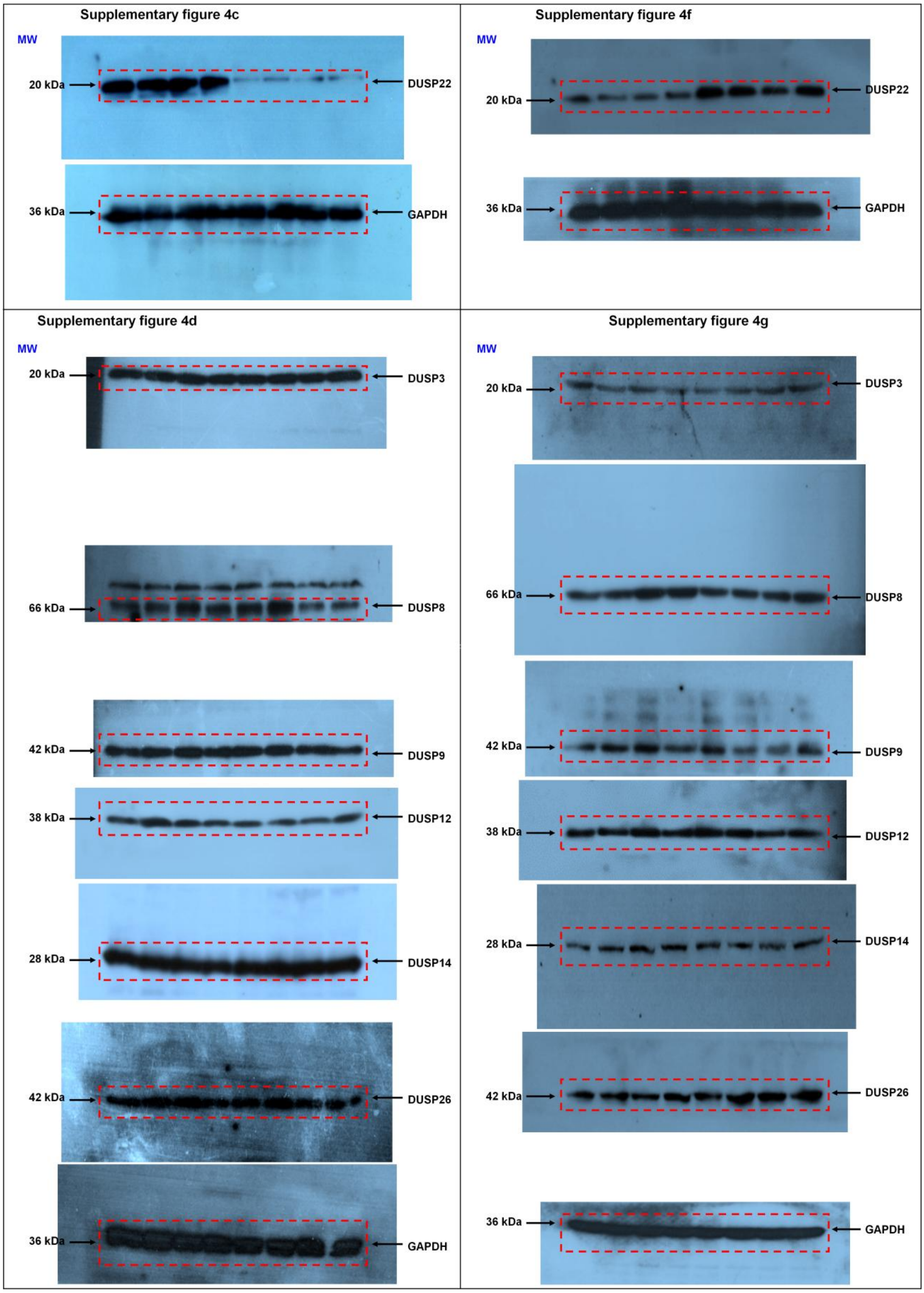

Western blots for supplementary figure 5

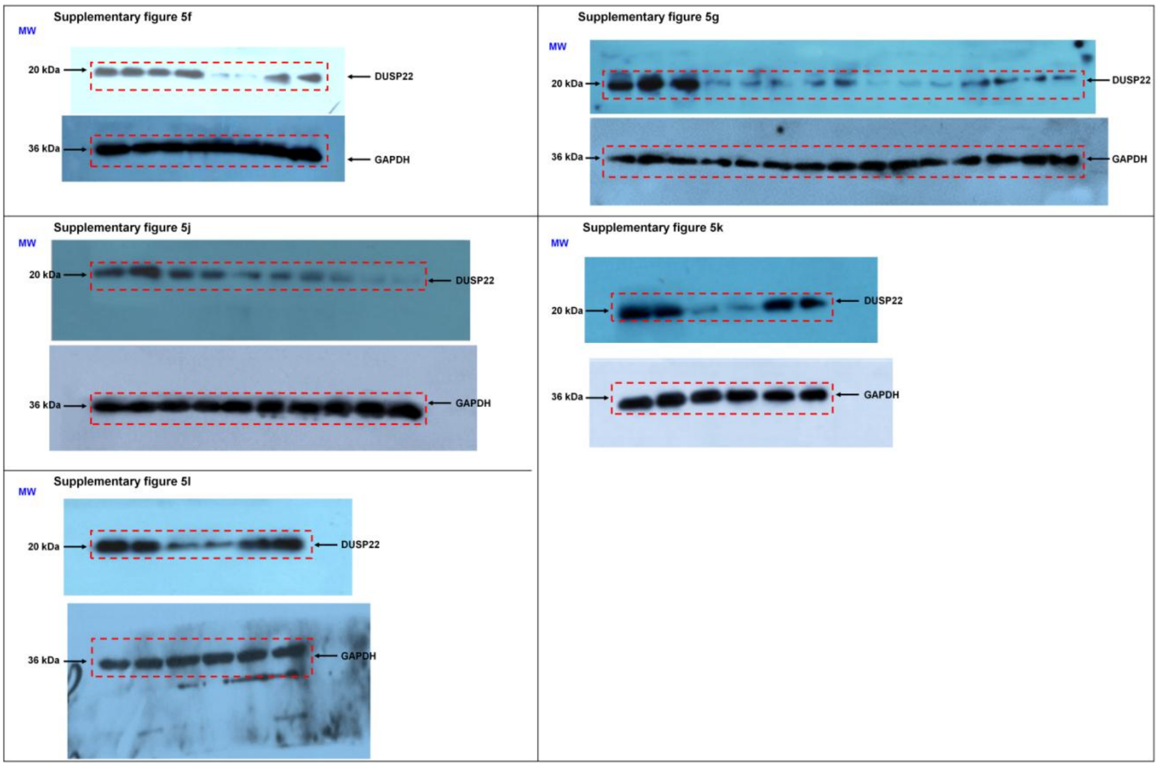

Western blots for supplementary figure 6

Supplementary figure 6c

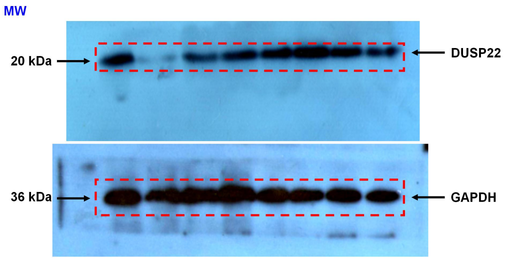

Supplementary figure 6d

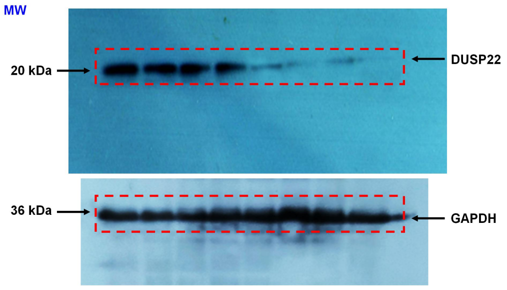

Supplementary figure 6e

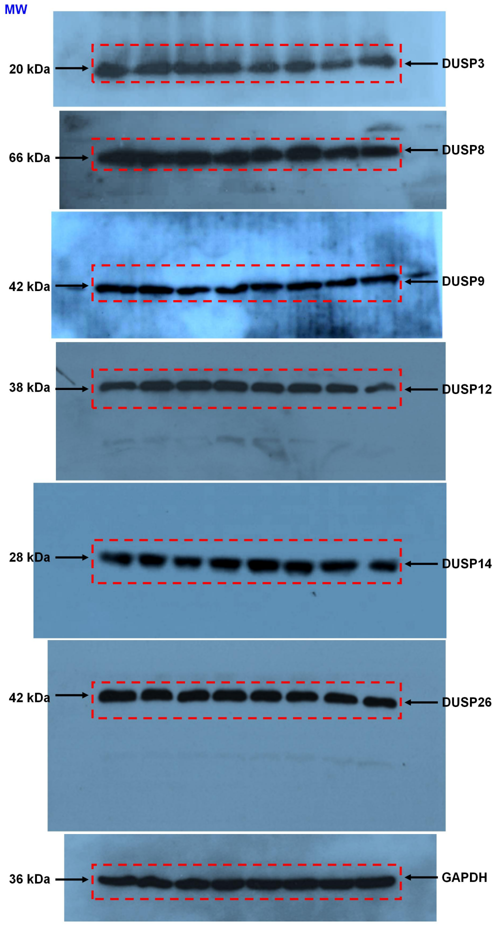

Western blots for supplementary figure 9

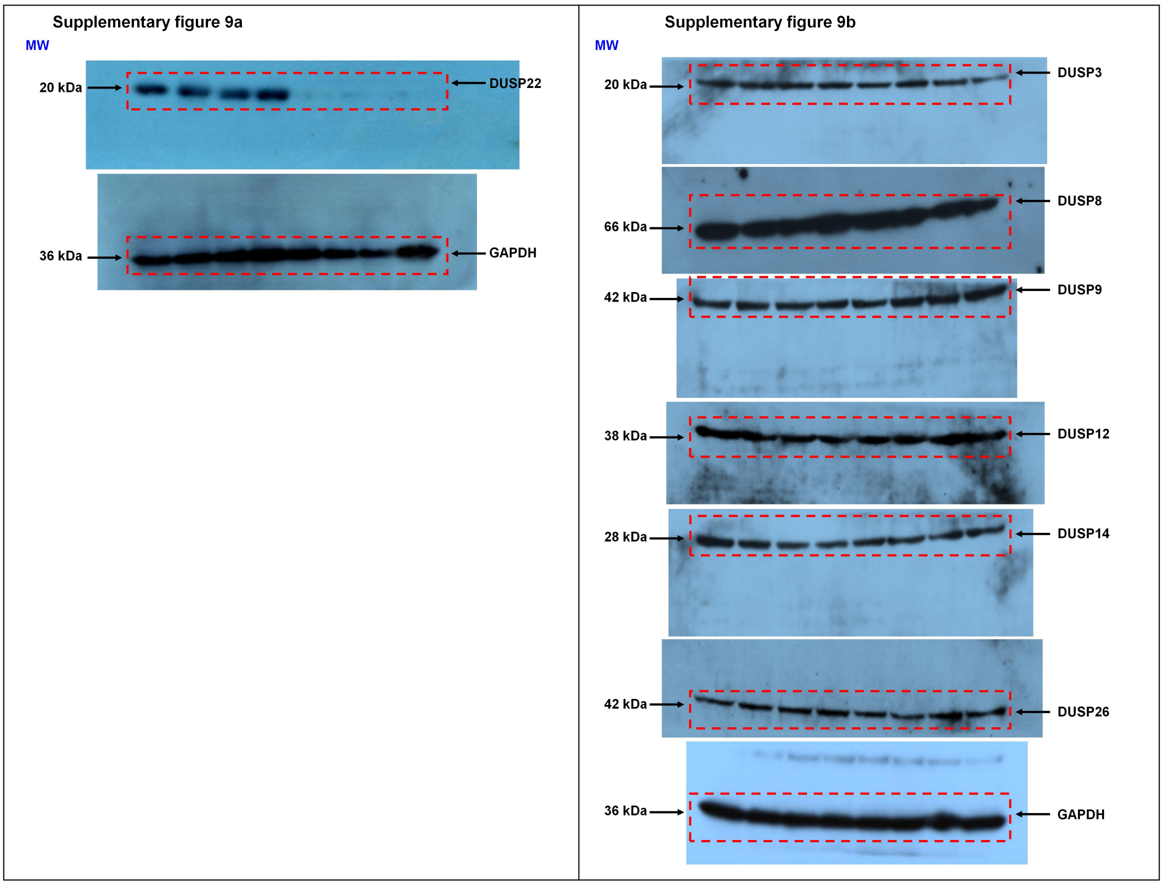

Western blots for supplementary figure 10

Supplementary figure 10c

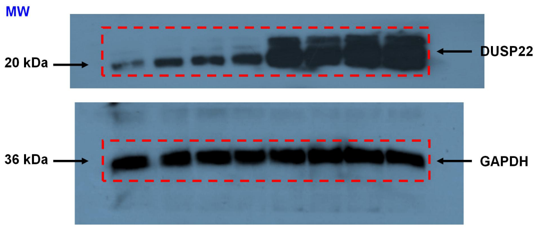

Supplementary figure 10e

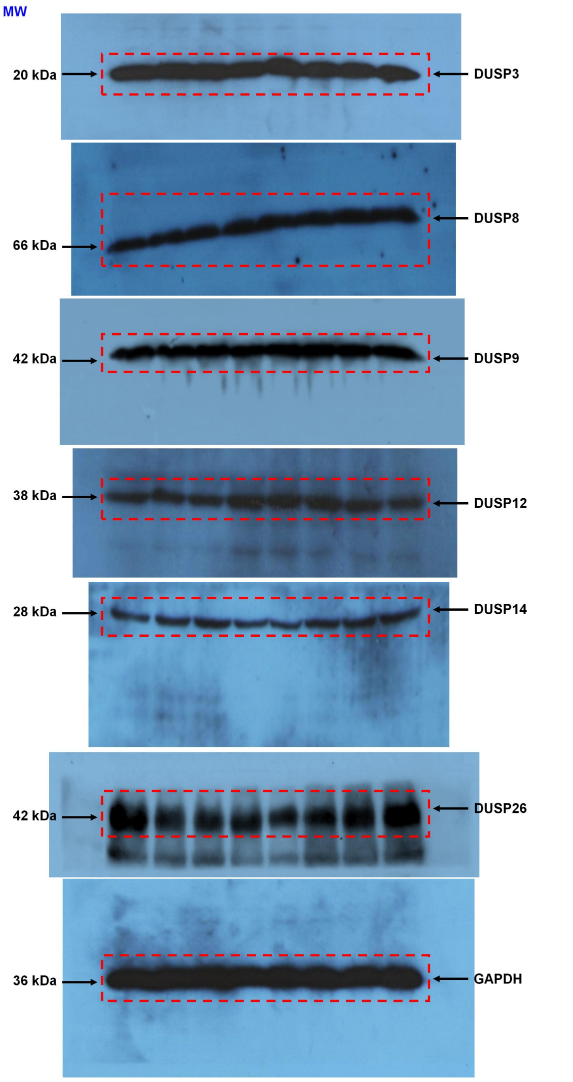

Western blots for supplementary figure 13

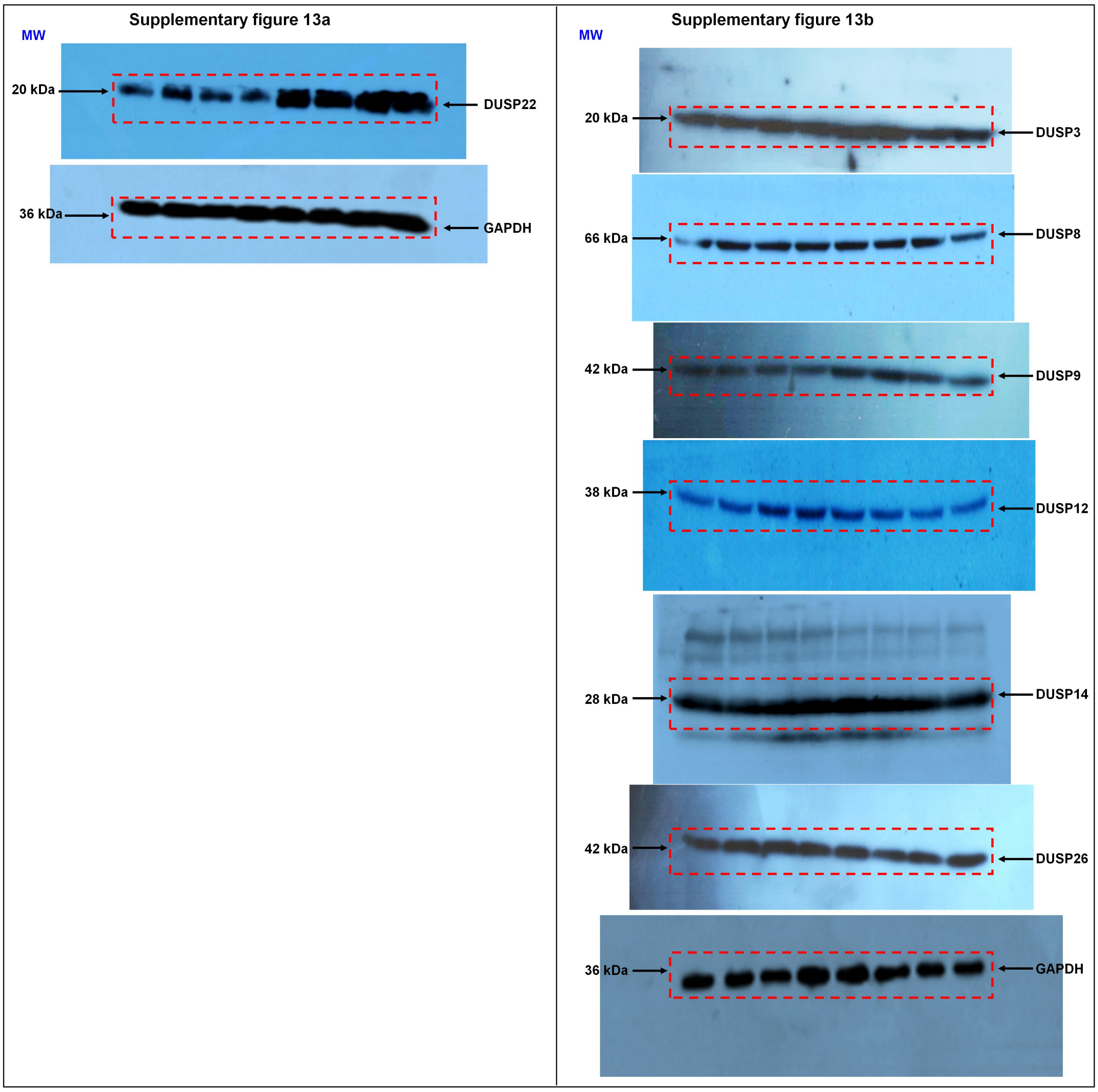

Western blots for supplementary figure 14

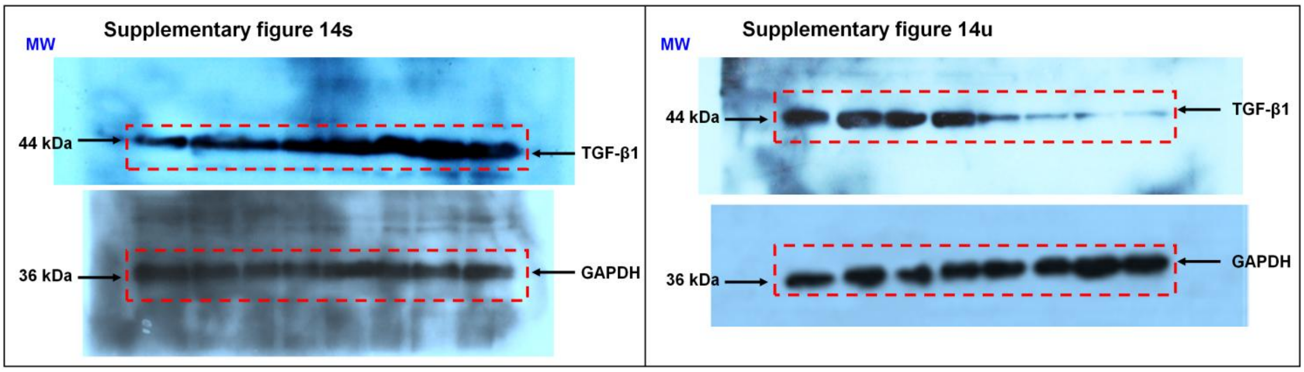

Western blots for supplementary figure 15

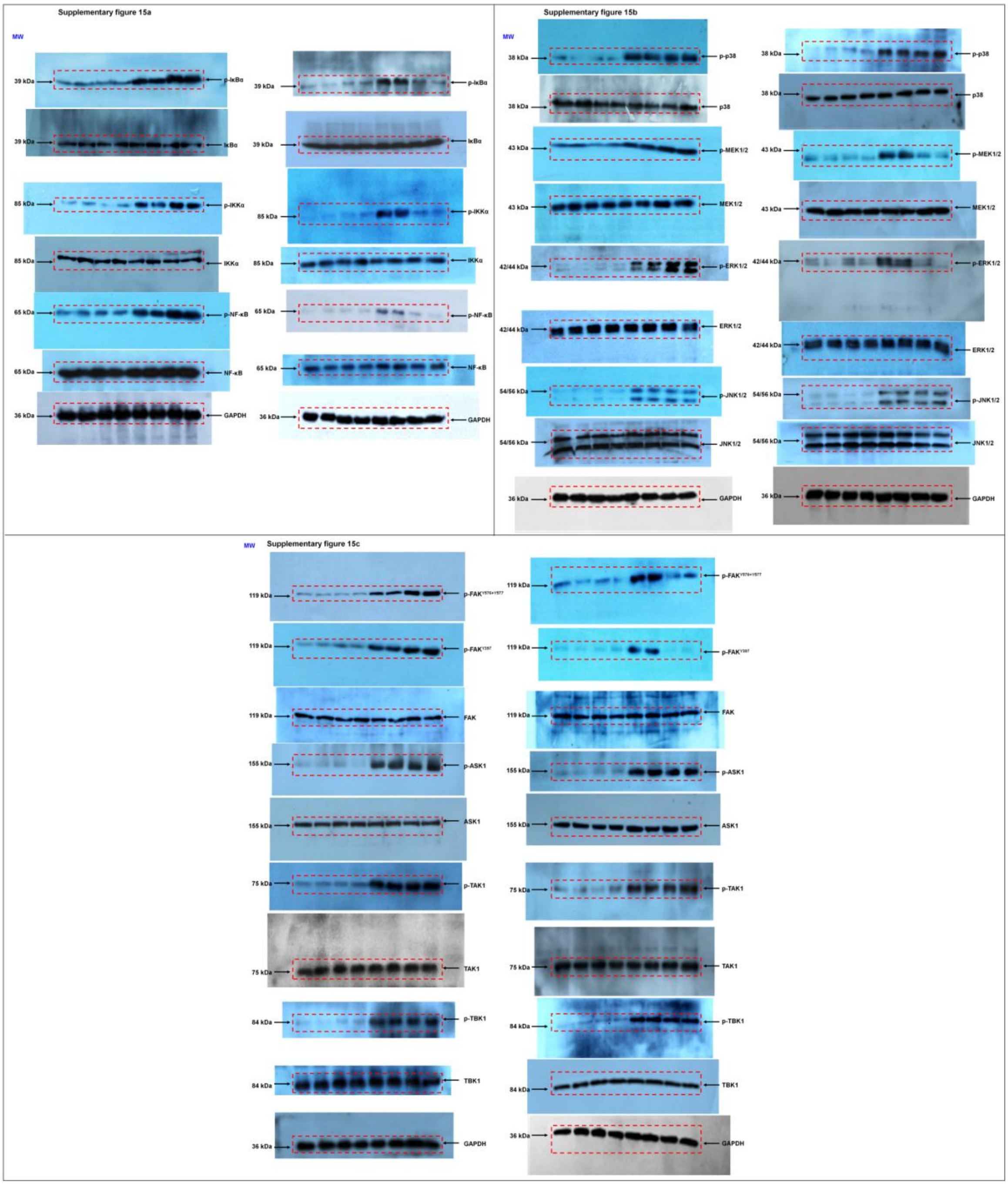

Western blots for supplementary figure 16

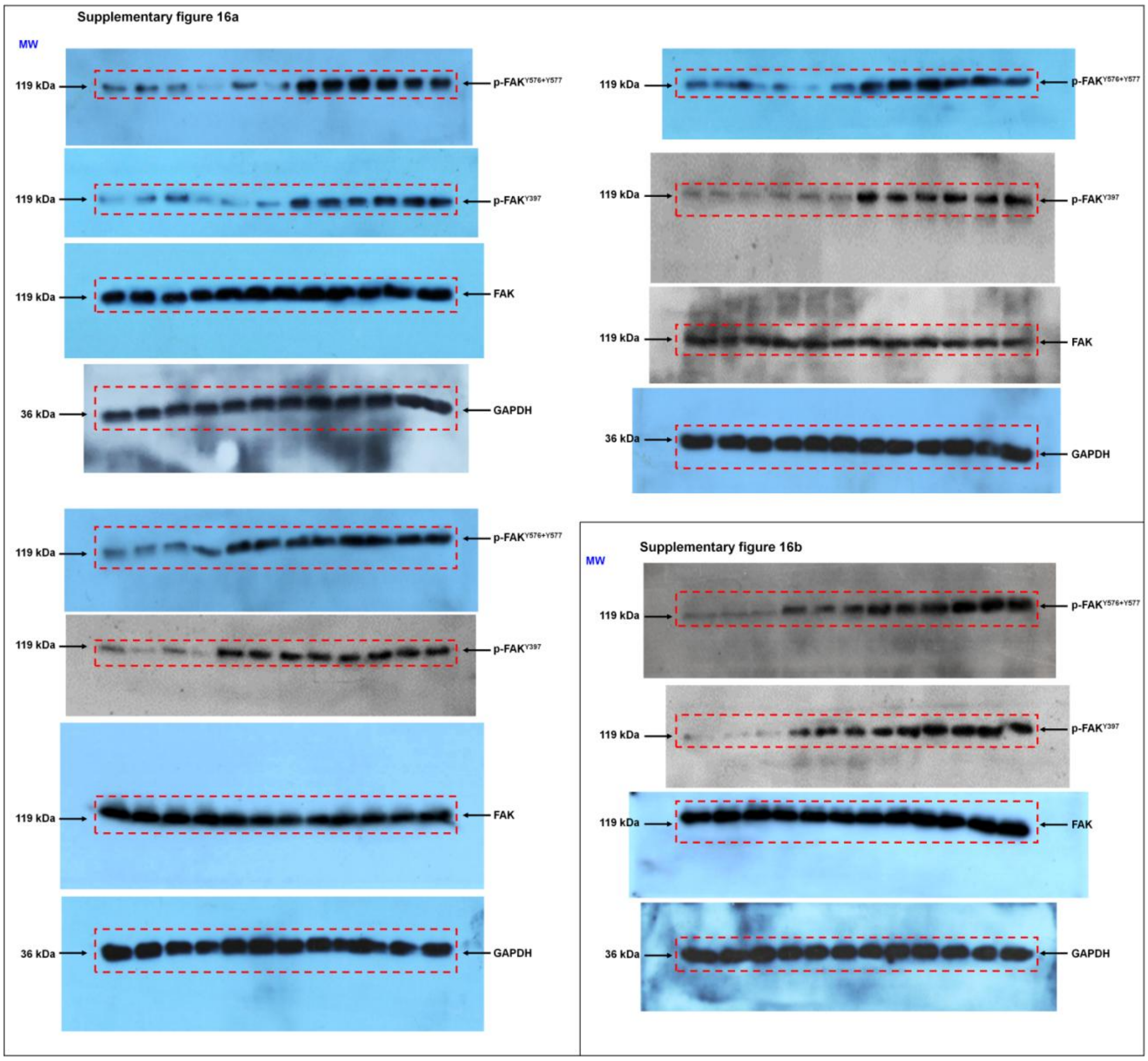

Western blots for supplementary figure 17

Supplementary figure 17b

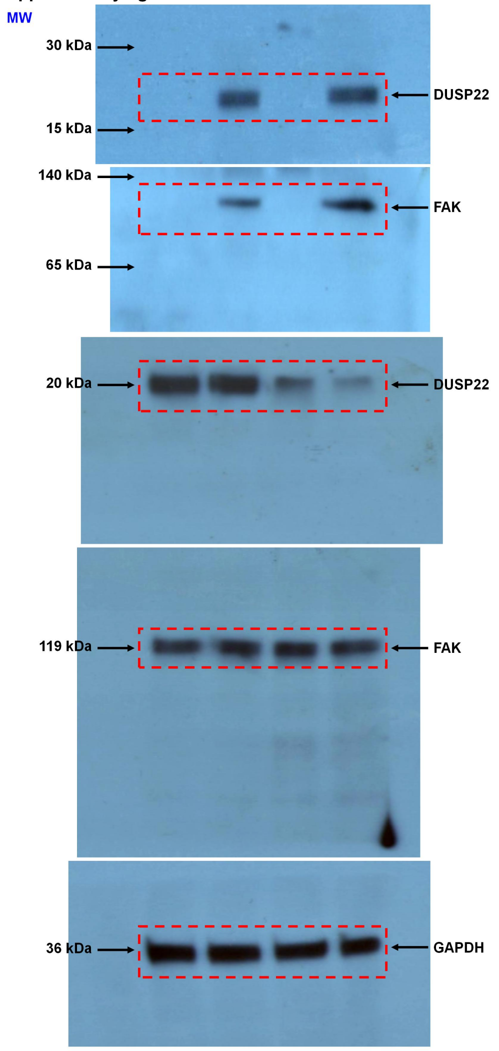

Western blots for supplementary figure 18

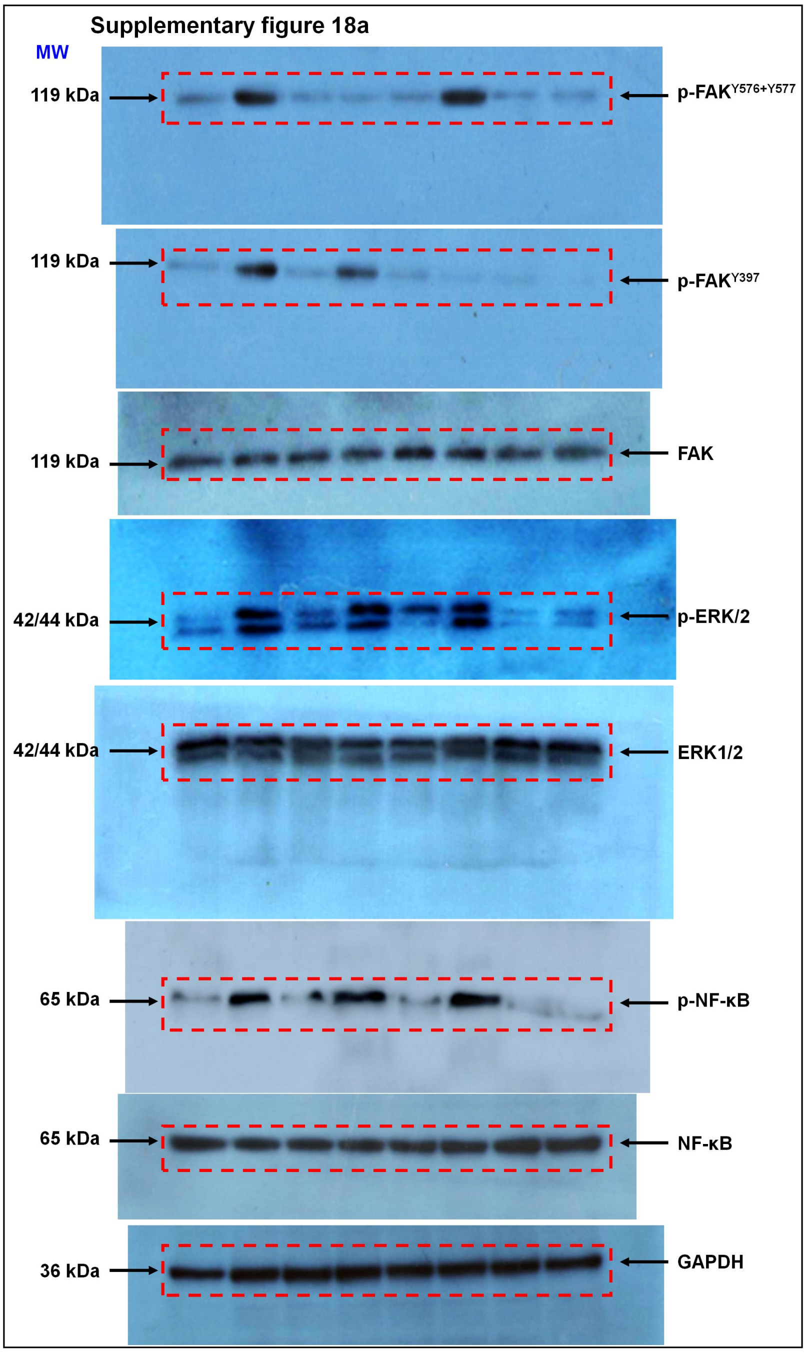

Western blots for supplementary figure 19

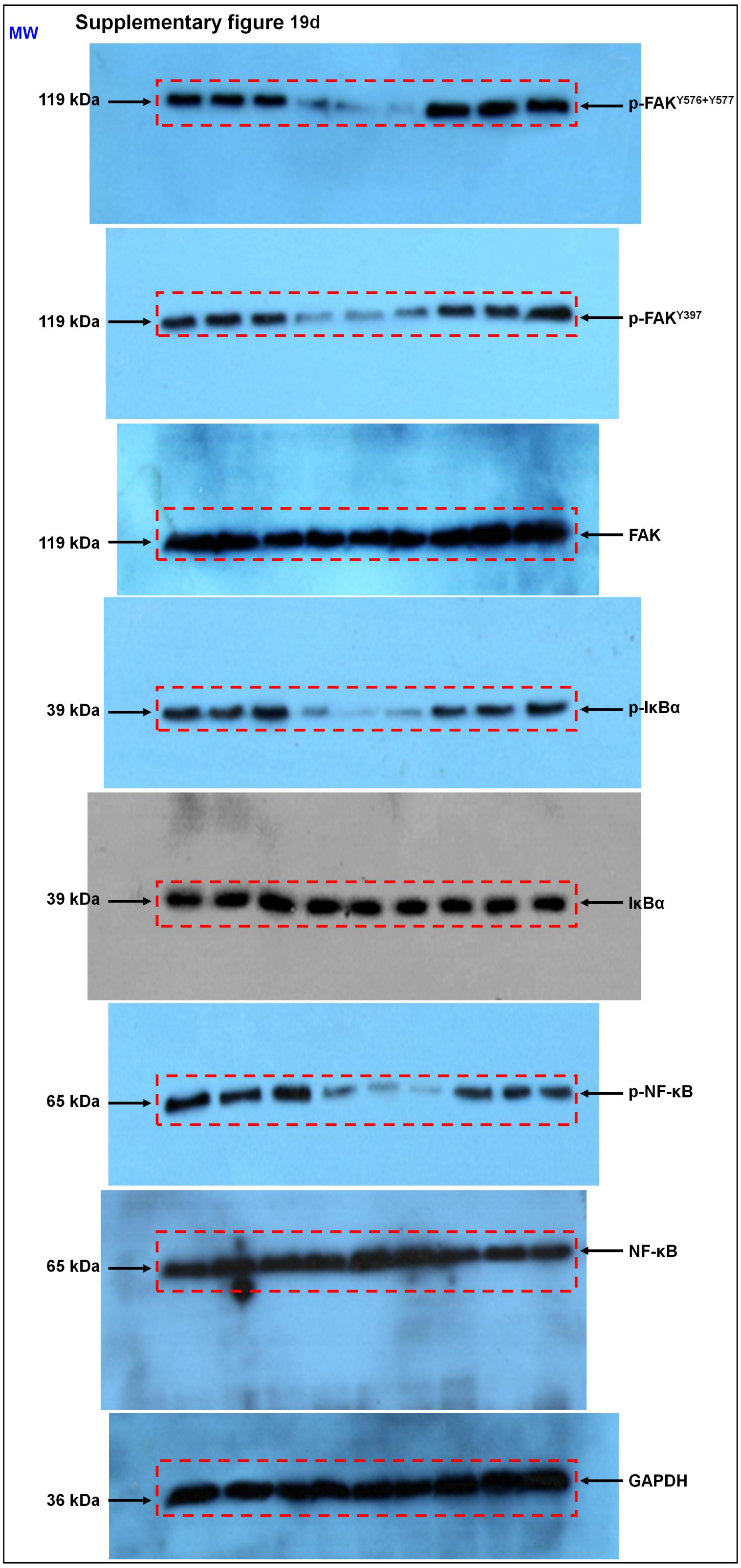

Western blots for supplementary figure 20

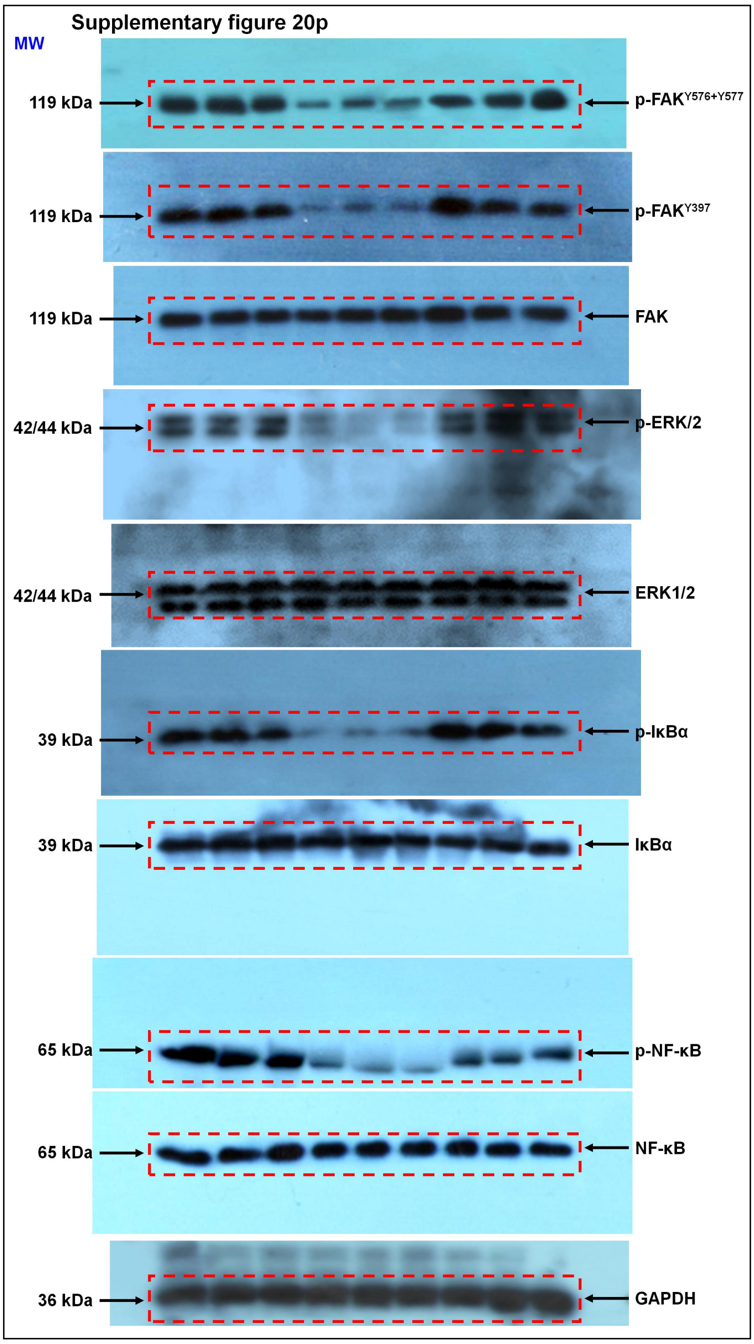

Western blots for supplementary figure 21

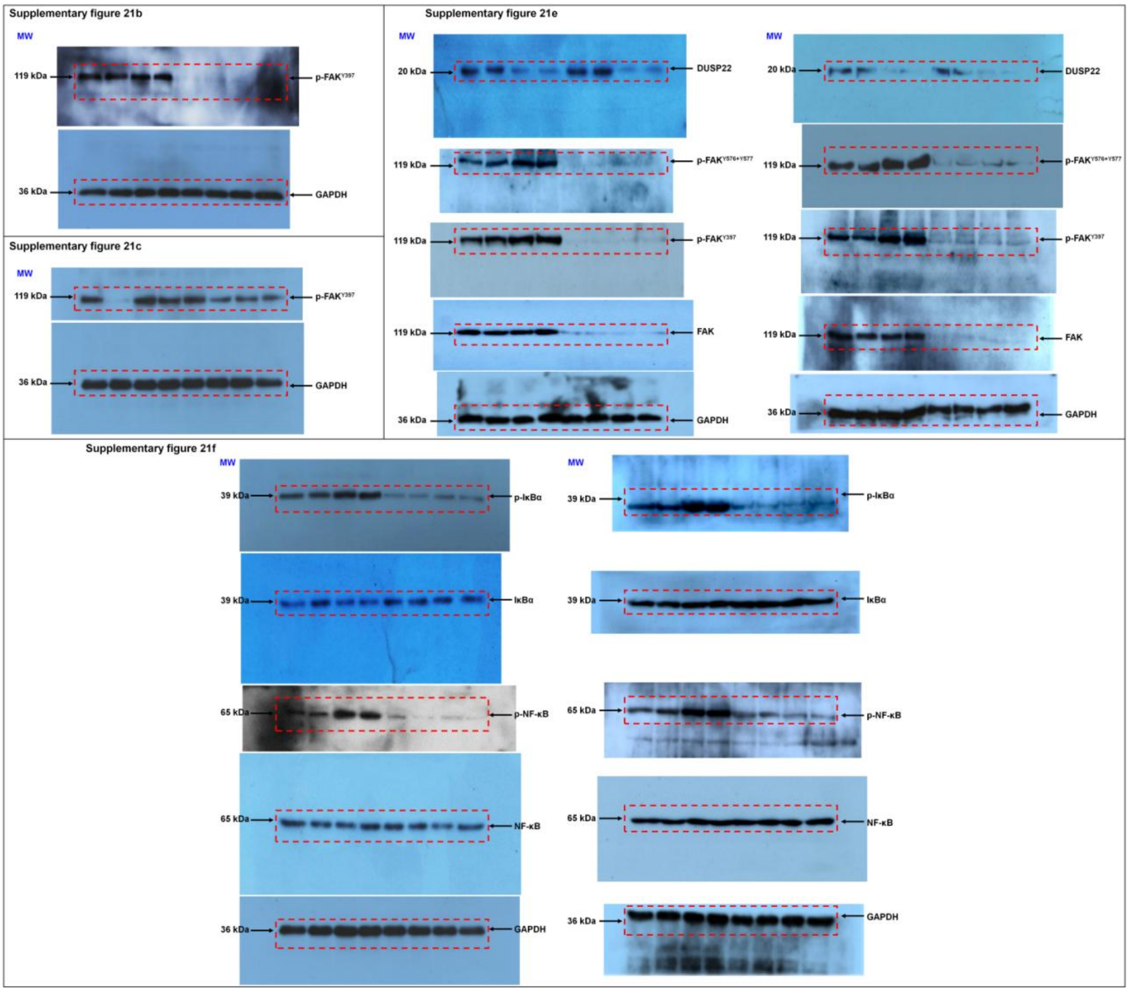

Western blots for supplementary figure 22

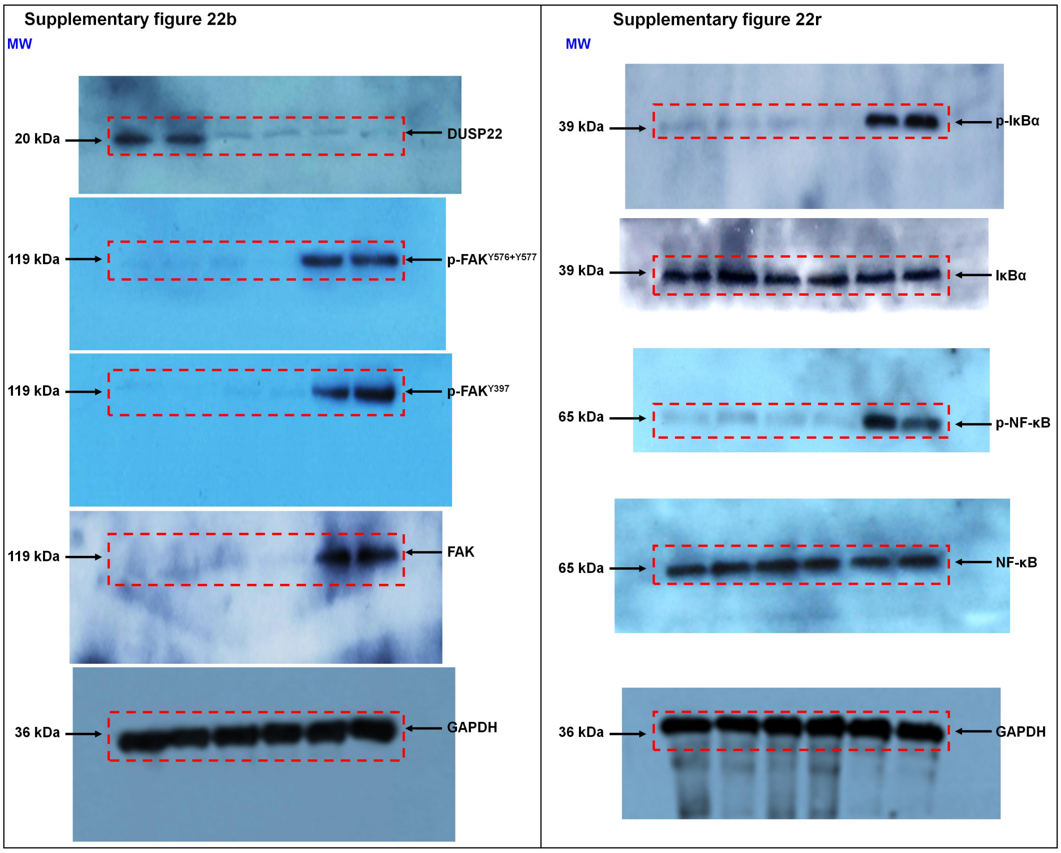

Western blots for supplementary figure 23

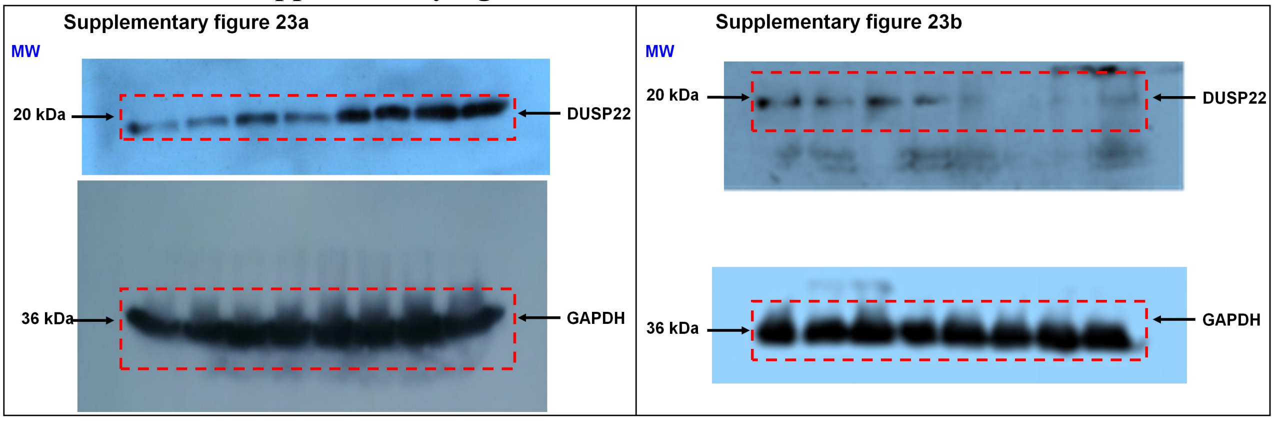

Western blots for supplementary figure 24

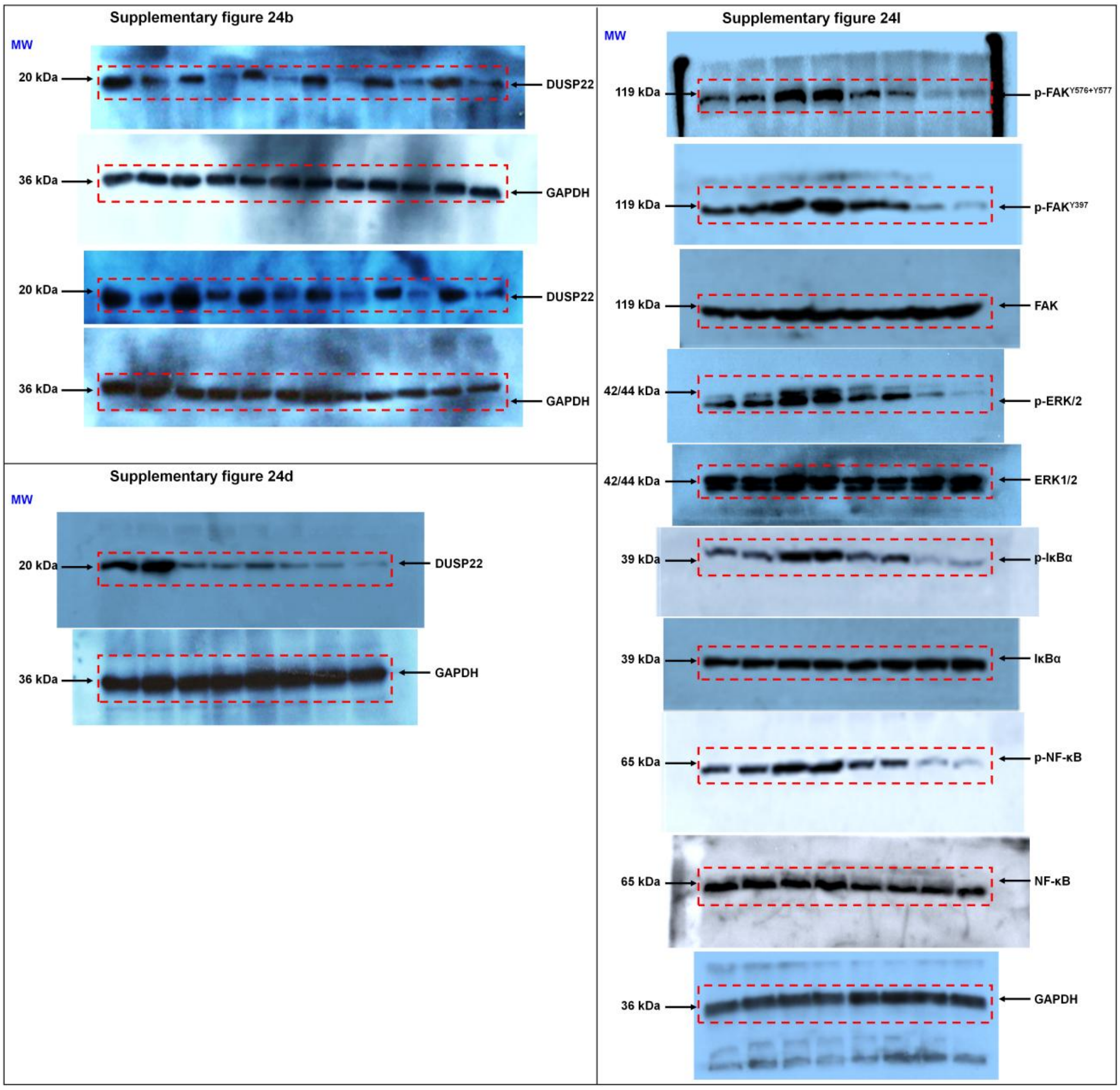

Western blots for supplementary figure 25

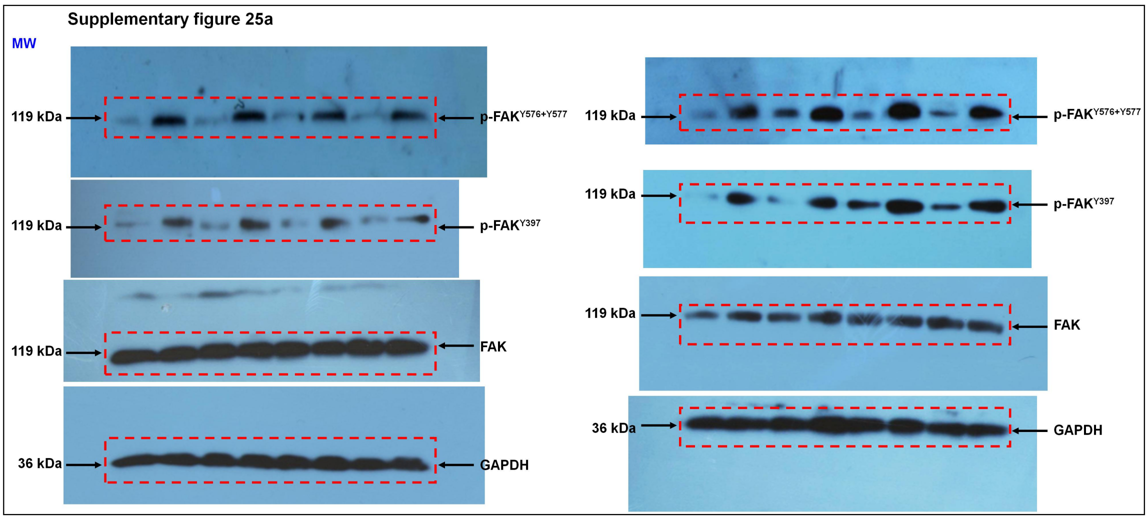

Supplement: Supplementary file 1 — Supplementary Information [file 41467_2022_33493_MOESM1_ESM.pdf]
