# Supplementary material for: Hepatocyte phosphatase DUSP22 mitigates NASH-HCC progression by targeting FAK
Source: Nat Commun. 2022 Oct 8;13:5945. doi: 10.1038/s41467-022-33493-5 (PMC9547917; doi:10.1038/s41467-022-33493-5)
Supplement: Supplementary file 2 — Reporting Summary [file 41467_2022_33493_MOESM2_ESM.pdf]

# nature portfolio

Corresponding author(s): NCOMMS-21-21899A

Last updated by author(s): Jun 21, 2022

# Reporting Summary

Nature Portfolio wishes to improve the reproducibility of the work that we publish. This form provides structure for consistency and transparency in reporting. For further information on Nature Portfolio policies, see our Editorial Policies and the Editorial Policy Checklist.

## Statistics

For all statistical analyses, confirm that the following items are present in the figure legend, table legend, main text, or Methods section.

| n/a | Confirmed | |
|---|---|---|
| ☐ | ☒ | The exact sample size (*n*) for each experimental group/condition, given as a discrete number and unit of measurement |
| ☐ | ☒ | A statement on whether measurements were taken from distinct samples or whether the same sample was measured repeatedly |
| ☐ | ☒ | The statistical test(s) used AND whether they are one- or two-sided<br>*Only common tests should be described solely by name; describe more complex techniques in the Methods section.* |
| ☐ | ☒ | A description of all covariates tested |
| ☐ | ☒ | A description of any assumptions or corrections, such as tests of normality and adjustment for multiple comparisons |
| ☐ | ☒ | A full description of the statistical parameters including central tendency (e.g. means) or other basic estimates (e.g. regression coefficient) AND variation (e.g. standard deviation) or associated estimates of uncertainty (e.g. confidence intervals) |
| ☐ | ☒ | For null hypothesis testing, the test statistic (e.g. *F*, *t*, *r*) with confidence intervals, effect sizes, degrees of freedom and *P* value noted<br>*Give P values as exact values whenever suitable.* |
| ☒ | ☐ | For Bayesian analysis, information on the choice of priors and Markov chain Monte Carlo settings |
| ☒ | ☐ | For hierarchical and complex designs, identification of the appropriate level for tests and full reporting of outcomes |
| ☐ | ☒ | Estimates of effect sizes (e.g. Cohen's *d*, Pearson's *r*), indicating how they were calculated |

*Our web collection on statistics for biologists contains articles on many of the points above.*

## Software and code

Policy information about availability of computer code

| | |
|---|---|
| Data collection | Microsoft Excel & Word-Microsoft Office 2017; Image J 1.52v, NIH; EndNote X9, Thomson Router; R (V 2.15.2); Olympus Confocal FV3000, Olympus; Olympus fluorescence microscope IX70, Olympus; Olympus optical microscope CX23, Olympus; ACCU-CHEK Performa, Roche Diabetes Care GmbH; ABI PRISM 7900HT Detection Systems, Applied Biosystems |
| Data analysis | SPSS (Statistical Product and Service Solutions) Statistics Software (Version 26.0.0.2, Mac OS X Snow Leopard; IBM); Image (Version J 1.52g, NIH) was used for quantification of immunoblotting, and positive-staining cells in the fluorescence staining and cell diameter of adipocytes; Image-Pro Plus software (Version 6.0) was used for quantification of cells with positive-staining by immunohistochemistry assay; ABI PRISM 7900HT detection systems was used for collection of qPCR data; GraphPad Prism Software (Version 8.0 & 9.2.0, Mac OS X Snow Leopard; Graph Pad Software) was used for data analysis. |

For manuscripts utilizing custom algorithms or software that are central to the research but not yet described in published literature, software must be made available to editors and reviewers. We strongly encourage code deposition in a community repository (e.g. GitHub). See the Nature Portfolio guidelines for submitting code & software for further information.

## Data

Policy information about availability of data

All manuscripts must include a data availability statement. This statement should provide the following information, where applicable:

- Accession codes, unique identifiers, or web links for publicly available datasets
- A description of any restrictions on data availability
- For clinical datasets or third party data, please ensure that the statement adheres to our policy

All data that support the findings of this study are available.

# Field-specific reporting

Please select the one below that is the best fit for your research. If you are not sure, read the appropriate sections before making your selection.

- [x] Life sciences
- [ ] Behavioural & social sciences
- [ ] Ecological, evolutionary & environmental sciences

For a reference copy of the document with all sections, see nature.com/documents/nr-reporting-summary-flat.pdf

# Life sciences study design

All studies must disclose on these points even when the disclosure is negative.

| | |
|---|---|
| Sample size | Sample size was chosen taking in consideration the means of the target values between the experimental group and the control group, the mean standard error and the statistical analysis used. For animal studies, sample size was defined on the basis of past experience with the models, to allow a power ≥80% at the 5% significance level. For ethical reasons, the minimum number of animals necessary to achieve the scientific objectives was used. |
| Data exclusions | No data were excluded in the current work when performing the final statistical analysis, which have been provided state in "Statistical analysis" section. |
| Replication | All in vitro experiments were performed in triplicate unless specified in the figure legends. The detailed replication of each experiments has been provided in Figure Legend. |
| Randomization | For all in vivo animal studies, all mice were randomly assigned to different treatment groups. |
| Blinding | The investigators were unaware of the experimental groups in all the quantifications. |

# Reporting for specific materials, systems and methods

We require information from authors about some types of materials, experimental systems and methods used in many studies. Here, indicate whether each material, system or method listed is relevant to your study. If you are not sure if a list item applies to your research, read the appropriate section before selecting a response.

## Materials & experimental systems

| n/a | Involved in the study | |
|---|---|---|
| [ ] | [x] | Antibodies |
| [ ] | [x] | Eukaryotic cell lines |
| [x] | [ ] | Palaeontology and archaeology |
| [ ] | [x] | Animals and other organisms |
| [ ] | [x] | Human research participants |
| [x] | [ ] | Clinical data |
| [x] | [ ] | Dual use research of concern |

## Methods

| n/a | Involved in the study | |
|---|---|---|
| [x] | [ ] | ChIP-seq |
| [x] | [ ] | Flow cytometry |
| [x] | [ ] | MRI-based neuroimaging |

## Antibodies

| | |
|---|---|
| Antibodies used | The primary antibodies against anti-GAPDH (#2118, dilution 1:1000 ), anti-p-JNK (#4668, dilution 1:1000), anti-JNK (#9258, dilution 1:1000), anti-p38 (#8690, dilution 1:1000), anti-p-p38 (#4511, dilution 1:1000), anti-p-IκBα (#2859, dilution 1:1000), anti-IκBα (#4814, dilution 1:1000), anti-p-TBK1 (#5483, dilution 1:1000) and anti-p-NF-κB (#3033, dilution 1:500) were obtained from Cell Signaling Technology Inc (CST, Beverly, USA). Antibodies against anti-IKKα (#ab32041, dilution 1:1000), anti-MEK1/2 (#ab178876, dilution 1:1000), anti-p-MEK1/2 (#ab278564, dilution 1:1000), anti-ERK1/2 (#ab184699, dilution 1:1000), anti-p-ERK1/2 (#ab201015, dilution 1:1000), anti-NF-κB (#ab16502, dilution 1:1000), anti-TGF-β1 (#ab179695, dilution 1:1000), anti-TAK1 (#ab50431, dilution 1:1000), anti-TBK1 (#ab40676, dilution 1:1000), anti-HA (#ab18181, dilution 1:1000), anti-Flag (#ab205606, dilution 1:1000), anti-CD11b (#ab133357, dilution 1:200), anti-DUSP22 (#ab70124, dilution 1:1000), and anti-DUSP3 (#ab248113, dilution 1:1000), anti-EGFP (#ab184601, dilution 1:50), anti-HNF4a (#ab201460, dilution 1:100), anti-CK19 (#ab52625, dilution 1:50) and anti-α-SMA (#ab124964, dilution 1:50) were purchased from Abcam (Cambridge, MA, USA). Antibodies against anti-p-IKKα (#PA5-36652, dilution 1:1000), anti-p-FAKY397 (#44-624G, dilution 1:1000), anti-p-FAKY576+Y577 (#PA5-37706, dilution 1:1000), anti-FAK (#PA5-88093, dilution 1:1000), anti-p-TAK1 (#PA5-99340, dilution 1:1000), anti-HA (#PA1-985, dilution 1:1000), anti-Flag (#MA1-91878, dilution 1:1000), anti-F4/80 (#41-4801-82, dilution 1:200), anti-Ki-67 (#PA5-19462, dilution 1:200) anti-p-ASK1 (#PA5-105027, dilution 1:1000), anti-ASK1 (#PA5-20200, dilution 1:1000), anti-DUSP8 (PA5-18007, dilution 1:1000), anti-DUSP9 (#PA5-106527, dilution 1:1000), anti-DUSP12 (#PA5-89113, dilution 1:1000), anti-DUSP14 (#PA5-15565, dilution 1:1000), anti-DUSP16 (#PA5-23140, dilution 1:1000) and anti-DUSP26 (#PA5-22013, dilution 1:1000), anti-Albumin (#PA5-89332, dilution 1:100) and anti-PECAM (#PA5-32321, dilution 1:100) were purchased from Thermo Fisher Scientific, Inc., Waltham, USA). Antibodies against anti-DUSP22 (#H00056940-B01P, dilution 1:200 and #NBP1-83078, dilution 1:200) were obtained from Novus Biologicals (USA). Secondary antibodies including horseradish peroxidase (HRP)-conjugated anti-rabbit (#ab6721, dilution 1:5000 for WB, dilution 1:200 for IHC), anti-mouse (#ab6789, dilution 1:5000) or anti-goat (#ab6741, dilution 1:5000) were purchased from Abcam (Cambridge, MA, USA). Anti-rabbit IgG H&L |

(Alexa Fluor® 594) (#ab150080, dilution 1:300), anti-mouse IgG H&L (Alexa Fluor® 488) (#ab150113, dilution 1:300) or anti-rat IgG H&L (Alexa Fluor® 488) (#ab150165, dilution 1:300) secondary fluorescent antibodies were purchased from Abcam (Cambridge, MA, USA).

Validation

All antibodies used in our study have been validated and detailed information could be found on the websites from manufactures as listed below:
GAPDH (source: Rabbit; reactivity:H M R Mk B Pg; Filter:WB IHC IF F; https://www.cellsignal.com/products/primary-antibodies/gapdh-14c10-rabbit-mab/2118)
p-JNK (source: Rabbit; reactivity:H M R Dm Sc; Filter:WB IHC IP; https://www.cellsignal.com/products/primary-antibodies/phospho-sapk-jnk-thr183-tyr185-81e11-rabbit-mab/4668)
IκBα (source: Mouse; reactivity:H M R Mk B Pg; Filter:WB IP IHC IF F; https://www.cellsignal.com/products/primary-antibodies/ikba-l35a5-mouse-mab-amino-terminal-antigen/4814)
JNK (source: Rabbit; reactivity:H M R Hm Mk Mi; Filter:WB; https://www.cellsignal.com/products/primary-antibodies/jnk2-56g8-rabbit-mab/9258)
p-IκBα (source: Rabbit; reactivity:H M R Mk; Filter:WB IP; https://www.cellsignal.com/products/primary-antibodies/phospho-ikba-ser32-14d4-rabbit-mab/2859)
p-NF-κB (source: Rabbit; reactivity:H M R Hm Mk Pg; Filter:WB IP IF F; https://www.cellsignal.com/products/primary-antibodies/phospho-nf-kb-p65-ser536-93h1-rabbit-mab/3033)
p-TBK1 (source: Rabbit; reactivity:H M; Filter:WB IP IF F; https://www.cellsignal.com/products/primary-antibodies/phospho-tbk1-nak-ser172-d52c2-xp-rabbit-mab/5483)
p38 (source: Rabbit; reactivity:H M R Hm Mk B Pg; Filter:WB IHC IF F; https://www.cellsignal.com/products/primary-antibodies/p38-mapk-d13e1-xp-rabbit-mab/8690)
p-p38 (source: Rabbit; reactivity:H M R Mk Mi Pg Sc; Filter:WB IP IHC IF F; https://www.cellsignal.com/products/primary-antibodies/phospho-p38-mapk-thr180-tyr182-d3f9-xp-rabbit-mab/4511)
IKKα (source: Rabbit; reactivity:H M R; Filter:Flow-Cyt (Intra) WB IHC-P IP; https://www.abcam.cn/ikk-alpha-antibody-y463-ab32041.html)
MEK1/2 (source: Rabbit; reactivity:H M R; Filter:WB IHC-P ICC/IF IP Flow-Cyt (Intra); https://www.abcam.cn/mek1--mek2-antibody-epr16667-ab178876.html)
p-MEK1/2 (source: Rabbit; reactivity:H M; Filter:WB Flow-Cyt; https://www.abcam.cn/mek1-phospho-s221--mek2-phospho-s221-antibody-mek12s221-d3-ab278564.html)
anti-ERK1/2 (source: Rabbit; reactivity:H M R; Filter:Flow-Cyt (Intra) IP ICC/IF WB; https://www.abcam.cn/erk1--erk2-antibody-epr17526-ab184699.html)
p-ERK1/2 (source: Rabbit; reactivity:H M R; Filter: ICC/IF IP IHC-P WB Dot-blot; https://www.abcam.cn/erk1-phospho-t202--erk2-phospho-t185-antibody-epr19401-ab201015.html)
NF-κB (source: Rabbit; reactivity:H M; Filter:ICC/IF IHC-P WB IP; https://www.abcam.cn/nf-kb-p65-antibody-ab16502.html)
TGF-β1 (source: Rabbit; reactivity:H M R; Filter: WB; https://www.abcam.cn/tgf-beta-1-antibody-epr18163-ab179695.html)
TAK1 (source: Rabbit; reactivity:H M; Filter:WB; https://www.abcam.cn/tak1-antibody-ab50431.html)
TBK1 (source: Rabbit; reactivity:H M R; Filter:ICC/IF WB IHC-P; https://www.abcam.cn/naktbk1-antibody-ep611y-ab40676.html)
HA (source: Mouse; reactivity:Species independent; Filter:WB ICC/IF; https://www.abcam.cn/ha-tag-antibody-hac5-ab18181.html)
Flag (source: Rabbit; reactivity:Species independent; Filter:WB ICC/IF Flow-Cyt IHC-P IP; https://www.abcam.cn/ddddk-tag-binds-to-flag-tag-sequence-antibody-epr20018-251-ab205606.html)
CD11b (source: Rabbit; reactivity:H M R; Filter:WB IHC-P; https://www.abcam.cn/cd11b-antibody-epr1344-ab133357.html)
DUSP22 (source: Rabbit; reactivity:H M; Filter:ICC/IF WB; https://www.abcam.cn/dusp22-antibody-ab70124.html)
DUSP3 (source: Rabbit; reactivity:H M; Filter:IHC-P WB; https://www.abcam.cn/dusp3-antibody-epr5492-bsa-and-azide-free-ab248113.html)
EGFP (source: Mouse; reactivity:Species independent; Filter:WB ICC/IF; https://www.abcam.cn/egfp-antibody-f56-6a123-ab184601.html)
HNF4a (source: Rabbit; reactivity:H M R; Filter:Flow-Cyt (Intra) WB IHC-P ICC/IF; https://www.abcam.cn/hnf-4-alpha-antibody-epr16885-99-ab201460.html)
CK19 (source: Rabbit; reactivity:H M; Filter:Flow-Cyt (Intra) ICC/IF WB IHC-P; https://www.abcam.cn/cytokeratin-19-antibody-ep1580y-cytoskeleton-marker-ab52625.html)
α-SMA (source: Rabbit; reactivity:H M R; Filter:WB IHC-P ICC/IF Flow-Cyt (Intra); https://www.abcam.cn/alpha-smooth-muscle-actin-antibody-epr5368-ab124964.html)
p-IKKα (source: Rabbit; reactivity:H M R; Filter:WB IHC-P; https://www.thermofisher.cn/cn/zh/antibody/product/Phospho-IKK-alpha-Thr23-Antibody-Polyclonal/PA5-36652)
p-FAKY397 (source: Rabbit; reactivity:H M R C FF X; Filter:WB IHC ICC/IF; https://www.thermofisher.cn/cn/zh/antibody/product/Phospho-FAK-Tyr397-Antibody-Polyclonal/44-624G)
p-FAKY576+Y577 (source: Rabbit; reactivity:H M R; Filter:WB ICC/IF; https://www.thermofisher.cn/cn/zh/antibody/product/Phospho-FAK-Tyr576-Tyr577-Antibody-Polyclonal/PA5-37706)
FAK (source: Rabbit; reactivity:H M R; Filter:WB ICC/IF IP; https://www.thermofisher.cn/cn/zh/antibody/product/FAK-Antibody-Polyclonal/PA5-88093)
p-TAK1 (source: Rabbit; reactivity:H M R; Filter:WB ICC/IF IHC-P; https://www.thermofisher.cn/cn/zh/antibody/product/Phospho-TAK1-Thr187-Antibody-Polyclonal/PA5-99340)
HA (source: Rabbit; reactivity:Species independent; Filter:WB ChIP IP; https://www.thermofisher.cn/cn/zh/antibody/product/HA-Tag-Antibody-Polyclonal/PA1-985)
Flag (source: Mouse; reactivity:Species independent; Filter:WB ICC/IF IP; https://www.thermofisher.cn/cn/zh/antibody/product/DYKDDDDK-Tag-Antibody-clone-FG4R-Monoclonal/MA1-91878)
F4/80 (source: Rat; reactivity:H M; Filter:ICC/IF IHC-P; https://www.thermofisher.cn/cn/zh/antibody/product/F4-80-Antibody-clone-BM8-Monoclonal/41-4801-82)
Ki-67 (source: Rabbit; reactivity:H M Dg B Pg R Hr Rb Hm Sh; Filter:ICC/IF IHC-P; https://www.thermofisher.cn/cn/zh/antibody/product/Ki-67-Antibody-Polyclonal/PA5-19462)
p-ASK1 (source: Rabbit; reactivity:H M R; Filter:WB IHC-P ICC/IF; https://www.thermofisher.cn/cn/zh/antibody/product/Phospho-

ASK1-Ser966-Antibody-Polyclonal/PA5-105027)
ASK1 (source: Rabbit; reactivity:H M; Filter:WB ICC/IF; https://www.thermofisher.cn/cn/zh/antibody/product/ASK1-Antibody-Polyclonal/PA5-20200)
DUSP8 (source: Goat; reactivity:H M R; Filter:WB; https://www.thermofisher.cn/cn/zh/antibody/product/DUSP8-Antibody-Polyclonal/PA5-18007)
DUSP9 (source: Rabbit; reactivity:H M R; Filter:WB IHC-P ICC/IF; https://www.thermofisher.cn/cn/zh/antibody/product/DUSP9-Antibody-Polyclonal/PA5-106527)
DUSP12 (source: Rabbit; reactivity:H M; Filter:WB; https://www.thermofisher.cn/cn/zh/antibody/product/DUSP12-Antibody-Polyclonal/PA5-89113)
DUSP14 (source: Rabbit; reactivity:H M; Filter:WB IHC-P; https://www.thermofisher.cn/cn/zh/antibody/product/DUSP14-Antibody-Polyclonal/PA5-15565)
DUSP16 (source: Rabbit; reactivity:H M B; Filter:WB IHC-P; https://www.thermofisher.cn/cn/zh/antibody/product/DUSP16-Antibody-Polyclonal/PA5-23140)
DUSP26 (source: Rabbit; reactivity:H M R; Filter:WB ICC/IF; https://www.thermofisher.cn/cn/zh/antibody/product/DUSP26-Antibody-Polyclonal/PA5-22013)
Albumin (source: Rabbit; reactivity:H M R; Filter:WB IHC-P ICC/IF; https://www.thermofisher.cn/cn/zh/antibody/product/Albumin-Antibody-Polyclonal/PA5-89332)
PECAM (source: Rabbit; reactivity:H M R Pg; Filter:WB IHC-P ICC/IF; https://www.thermofisher.cn/cn/zh/antibody/product/CD31-Antibody-Polyclonal/PA5-32321)
DUSP22 (source: Mouse; reactivity:H; Filter:WB ICC/IF; https://www.novusbio.com/products/dusp22-antibody_h00056940-b01p)
DUSP22 (source: Rabbit; reactivity:H M R; Filter:WB ICC/IF IHC IHC-P; https://www.novusbio.com/products/dusp22-antibody_nbp1-83078)
Goat anti-Rabbit IgG (source: Goat; Conjugate: HRP;Suitable for: IHC-P, WB, ELISA, Immunomicroscopy, Dot blot, ICC, IHC-Fr; https://www.abcam.com/goat-rabbit-igg-hl-hrp-ab6721.html)
Goat anti-Mouse IgG (source: Goat; Conjugate: HRP;Suitable for: ICC, IP, Dot blot, ELISA, IHC-P, IHC-Fr, Immunomicroscopy, WB; https://www.abcam.com/goat-mouse-igg-hl-hrp-ab6789.html)
Rabbit Anti-Goat IgG (source: Rabbit; Conjugate: HRP;Suitable for:Dot blot, ELISA, IHC-P, IHC-Fr, Immunomicroscopy, ICC/IF, WB; https://www.abcam.com/rabbit-goat-igg-hl-hrp-ab6741.html)
Goat anti-Rabbit IgG H&L (source: Goat; Conjugate: Alexa Fluor® 594;Suitable for:IHC-Fr, ICC/IF, ELISA, IHC-P, Flow Cyt; https://www.abcam.com/goat-rabbit-igg-hl-alexa-fluor-594-ab150080.html)
Goat anti-Mouse IgG H&L (source: Goat; Conjugate: Alexa Fluor® 488;Suitable for:IHC-Fr, ICC/IF, ELISA, Flow Cyt, IHC-P; https://www.abcam.com/goat-mouse-igg-hl-alexa-fluor-488-ab150113.html)
Goat anti-Rat IgG H&L (source: Goat; Conjugate: Alexa Fluor® 488;Suitable for:ICC/IF, Flow Cyt, ELISA, IHC-P, IHC-Fr; https://www.abcam.com/goat-rat-igg-hl-alexa-fluor-488-preadsorbed-ab150165.html).
Application Key: WB-Western Blot; IP-Immunoprecipitation; IHC-Immunohistochemistry; ChIP-Chromatin Immunoprecipitation; ICC-Immunocytochemistry; IF-Immunofluorescence; Flow Cyt-Flow Cytometry.
Species Cross-Reactivity Key: H-Human; M-Mouse; R-Rat; Hm-Hamster; Mk-Monkey; C-Chicken; B-Bovine; Dg-Dog; Pg-Pig; Hr-Horse; Rb-Rabbit; Sh-Sheep; FF-fruit fly.
All the antibodies used are produced by trusted producers and validation protocols with WB, IP and IHC/IF were available in the website of the companies as well as in previously published papers.

# Eukaryotic cell lines

Policy information about cell lines

| | |
|---|---|
| Cell line source(s) | Human L02 cells were purchased from the Type Culture Collection of the Chinese Academy of Sciences (Shanghai, China); human hepatic stellate cell (HSC) line LX2 was obtained from Merck Millipore (Shanghai, China); human HCC cell lines (Hep3B and HepG2) and human embryonic kidney 293 T (HEK293T) were obtained from American Type Culture Collection (ATCC; Manassas, USA); and human HCC cell line SMMC-7721 was purchased from the Shanghai Cell Bank Type Culture Collection Committee (CBTCCC, Shanghai, China). All these cell lines have been provided in "Cell Culture" section. |
| Authentication | All cell lines have been verified by morphology check under microscopy and short tandem-repeat DNA profiling before the study. |
| Mycoplasma contamination | Human L02 cell line, HSC line LX2, HEK293T and human HCC cell lines (Hep3B, HepG2 and SMMC-7721) were used immediately after being received from the Type Culture Collection of the Chinese Academy of Sciences (Shanghai, China), Merck Millipore (Shanghai, China), ATCC (Manassas, VA, USA) or CBTCCC (Shanghai, China), and certified as Mycoplasma free. |
| Commonly misidentified lines (See ICLAC register) | No commonly misidentified cell lines were used in this study. |

# Animals and other organisms

Policy information about studies involving animals; ARRIVE guidelines recommended for reporting animal research

| | |
|---|---|
| Laboratory animals | All in vivo experiments were conducted with the age-matched male mice.<br>Strains:<br>DUSP22flox/flox mice with C57BL/6N background were constructed using CRISPR/Cas9-regulated genome engineering system. The exon 3 of DUSP22 was selected as conditional knockout region (cKO). Briefly, the selected exon of DUSP22 were flanked by two loxP sites, and therefore two single guide RNAs (gRNA1# and gRNA2#) targeting DUSP22 introns were designed. The targeting vector containing DUSP22 exon 3 flanked by two loxP sites and the two homology arms was used as the template. The targeting vector, gRNA1# and gRNA2#, and together with Cas9 were co-injected into fertilized eggs for cKO mouse production. The obtained mice, |

which had exon 3 flanked by two loxP sites on one allele, were used to establish DUSP22flox/flox mice. Hepatocyte-specific DUSP22 deletion (DUSP22HepKO) mice were produced by mating DUSP22flox/flox mice with 6-8-weeks old albumin-Cre (Alb-Cre) mice (Jackson Laboratory, Bar Harbor, Maine, USA). A simple schematic diagram has been indicated in Supplementary Fig. S6A.
To obtain mice with conditional knock in of DUSP22, the RosaDUSP22 mice with C57BL/6N background were constructed using DUSP22 conditional knock in at the locus of Rosa26 in mice by CRISPR/Cas-regulated genome engineering system. In brief, the Rosa26-CAG-loxp-STOP-loxp-mDUSP22-pA cassette was cloned into intron 1 of Rosa26. Also, to engineer the targeting vector, homology arms were then constructed by PCR using BAC clone as template. Thereafter, the targeting vector, gRNA, and Cas9 were co-injected into fertilized eggs for RosaDUSP22 mouse production. In indicated experiments, the conditional over-expression of DUSP22 in hepatocyte (DUSP22HepOE) was induced through injection of adenoassociated virus-serotype 8 (AAV8)-thyroxine binding globulin (TBG) promoter-Cre recombinase vector (AAV8-TBG-Cre) via intravenous injection and then determined using immunoblotting analysis. A simple schematic diagram has been indicated in Supplementary Fig. S10A. RosaDUSP22 mice littermates without AAV injection were used as controls for the obtained DUSP22HepOE mice.
The hepatocyte-specific FAK-knockout (FAKHepKO) mice with a deletion in the exon 4 of FAK were produced using protocols similar to the one described for the establishment of the DUSP22flox/flox mice and DUSP22HepKO mice. Briefly, FAKflox/flox mice was designed and constructed by CRISPR/Cas-mediated genome engineering system. The exon 4 of FAK gene was selected as conditional knockout region. To engineer the targeting vector, homology arms and cKO region were generated by PCR using BAC clone from the C57BL/6N library as template. Then Cas9 and gRNA was co-injected into fertilized eggs with targeting vector for mice production. Then, the FAKflox/flox mice were crossed with Alb-Cre mice to produce hepatocyte-specific FAK deficiency mice (FAKHepKO). A simple schematic diagram has been indicated in Supplementary Fig. S21A. FAK-floxed mice littermates were used as controls for the obtained FAKHepKO mice.
The hepatocyte-specific DUSP22 (DUSP22HepKO) and FAK (FAKHepKO) double deletion (HepDKO) mice were generated through crossing DUSP22flox/flox mice with FAKHepKO mice.
Additionally, all the other normal wild-type (WT) C57BL/6N mice (6- to 8-week-old; 22-25 g body weight) used in the current study were purchased from Beijing Vital River Laboratory Animal Technology Co., Ltd. (Beijing, China). The age-matched (6- to 8-week-old) male ob/ob mice (#N000103) were purchased from Nanjing Biomedical Research Institute of Nanjing University).

Prior to all experiments proper starts, the mice were subjected to adapt to the living environment for 7 days. The mice were housed in a constant temperature, humidity (controlled by GREE central air-conditioner, #GMV-Pd250W/NaB-N1, China) and pathogen-free-controlled environment (25 ± 2°C, 50%-60%) cage with a standard 12 h light/12 h dark cycle, plenty of water and food (pathogen-free) in their cages. The 6–8-week-old WT male mice were fed with high-fat plus high-cholesterol diet (HFHC) (containing 42% saturated-fat, 14% protein, 44% carbohydrates and 0.2% cholesterol w/w) for 24 weeks to induce NASH. The mice fed with a normal chow diet (20% protein, 10% fat and 70% carbohydrate, #D12450H; Research Diets, New Brunswick, NJ, USA) for 24 weeks were defined as controls (NCD).

Wild animals

Our study did not involve wild animals.

Field-collected samples

No field-collected samples were included and used.

Ethics oversight

All mouse experiments and procedures were reviewed and approved by the Institutional Animal Care and Use Committee in Chongqing Key Laboratory of Medicinal Resources in the Three Gorges Reservoir Region, School of Biological and Chemical Engineering, Chongqing University of Education.

Note that full information on the approval of the study protocol must also be provided in the manuscript.

## Human research participants

Policy information about studies involving human research participants

Population characteristics

Human liver tissue samples were collected painlessly from adult patients with nonalcoholic fatty liver disease who underwent liver transplantation or liver biopsy. The corresponding control liver tissue was harvested from the donor who could not be used for liver transplantation because of non-hepatic reasons. Non-steatosis, and NASH liver samples were collected and included in this study. Briefly, specimens with a NASH activity score (NAS) of 0 were classified as nonsteatotic. Samples with a NAS ≥5 or a NAS of 3-4 but showing fibrosis were included in the NASH group. Steatotic liver samples from patients meeting any of the following criteria were excluded from the study: excessive alcohol consumption (>140 g for male or >70 g for female, per week), drug abuse, or viral infection (including infection with hepatitis B virus or hepatitis C virus). Human NAFLD-associated HCC tumor tissues and adjacent normal tissues were obtained from patients with biopsy-proven NAFLD-HCC. The characteristics, liver injury-associated serology and NAS for NASH patients were listed in Supplementary table S1 and S2, and clinical information of NAFLD-HCC subjects were displayed in Supplementary table S3.

Recruitment

All human samples and clinical information were obtained from the Shandong Cancer Hospital and Institute, Shandong First Medical University & Shandong Academy of Medical Sciences. Informed and written consent were obtained from all subjects or their family members before participating in this study. There was no self-selection bias or other biases that may be present and impact results.

Ethics oversight

All procedures associating with human subjects used in this study were based on Declaration of Helsinki, and completely permitted by the Academic Research Ethics Committee in Shandong Cancer Hospital and Institute, Shandong First Medical University & Shandong Academy of Medical Sciences and Chongqing Key Laboratory of Medicinal Resources in the Three Gorges Reservoir Region.

Note that full information on the approval of the study protocol must also be provided in the manuscript.
